# Urea Synthesis from Isocyanides and *O*-Benzoyl Hydroxylamines Catalyzed by a Copper Salt

**DOI:** 10.3390/molecules27238219

**Published:** 2022-11-25

**Authors:** Ning Yu, Jing-Fang Lv, Shi-Mei He, Yanyan Cui, Ye Wei, Kun Jiang

**Affiliations:** 1 School of Chemistry and Chemical Engineering, Southwest University, Chongqing 400715, China; 2 Department of Cell Biology and Genetics, Chongqing Medical University, Chongqing 400016, China

**Keywords:** copper catalysis, isocyanides, hydroxylamines, ureas, insertion

## Abstract

In the presence of CuOAc, a series of unsymmetric ureas can be generated in moderate to good yields under mild reaction conditions (10 mol% of CuOAc, 2 equiv *t*-BuONa or PhONa, 30 °C), using aryl isocyanides and *O*-benzoyl hydroxylamines as the readily accessible starting materials. The reactions might undergo a cascade process involving isocyanide insertion into the N-O bond and Mumm-type rearrangement. This work represents a rare example of isocyanide insertion into N-O bonds, which would extend isocyanide insertion chemistry.

## 1. Introduction

**Scheme 1:**
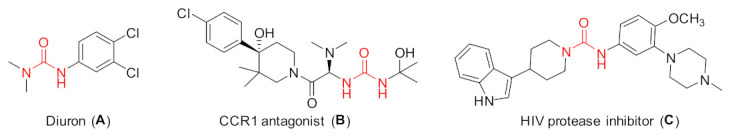
Biologically active compounds containing the urea unit.

**Scheme 2:**
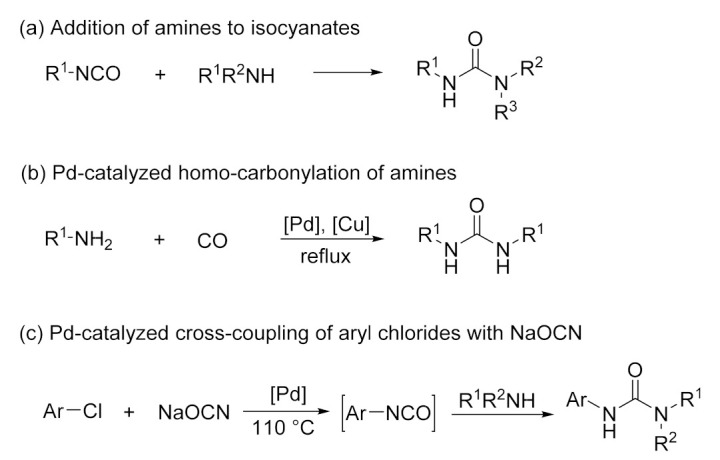
Some methods for urea synthesis.

Ureas are common core structures of a wide variety of pharmaceuticals, agrochemicals, and functional materials. For example, compounds containing the urea substructure include an effective herbicide (Diuron, **A**) [1], CCR1 antagonist (**B**) [2], and HIV protease inhibitor (**C**) (Scheme 1) [3]. Ureas also provide useful synthons for the construction of a vast majority of valuable compounds [4,5]. Moreover, they are potential organocatalysts by serving as hydrogen-bond donors [6] and ligands for transition metals [7]. Therefore, numerous methods have successfully been explored to synthesize ureas, such as the addition of amines to isocyanates or to carbonyldiimidazole (Scheme 2a) [8,9], direct carbonylation of primary amines with carbon monoxide (Scheme 2b) [10], and transition metal-catalyzed cross-coupling of aryl chlorides with sodium cyanate (Scheme 2c) [11]. Despite these achievements, these approaches have several limitations, including the use of toxic reagents and/or the requirement of high temperature and pressure, which results in limited reaction types and substrate scope, poor functional group tolerance, and/or structural diversity of the obtained ureas. Consequently, the development of a concise and new method for the preparation of ureas is highly desirable.

**Scheme 3:**
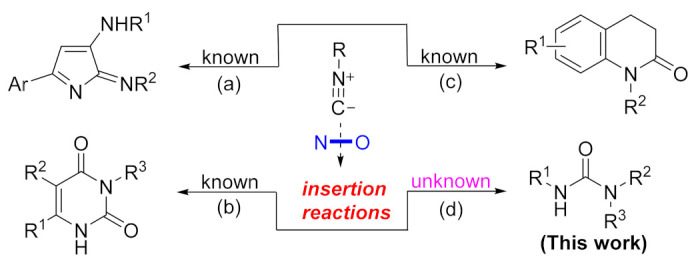
Some examples of isocyanide insertion into N-O bonds. (**a**) Pd-catalyzed reactions of oximes of isocyanides. (**b**) Ag-catalyzed difunctionalization of the isocyano group. (**c**) Cu-catalyzed difunctionalization of the isocyano group. (**d**) Cu-catalyzed urea synthesis.

Isocyanides are a class of synthetically useful synthons [12] that can participate in a series of transformations, such as multicomponent reactions [13], cycloaddition reactions [14,15], and insertion reactions [16]. Among insertion reactions, it is known that isocyanides can insert into heteroatom/C-H and C-heteroatom/metal bonds for the preparation of valuable compounds. Compared to these reactions, examples of isocyanide insertion into N-O bonds are rare. It is noteworthy that N-O bonds exist in a large number of organic molecules. As such, the application of N-O bond-containing molecules in isocyanide insertion reactions would provide many opportunities for the construction of valuable compounds, particularly N-containing molecules. For instance, Jiang and Wang independently developed Pd-catalyzed reactions of oximes of isocyanides for the synthesis of N-heterocycles (Scheme 3a) [17,18]. In addition, we recently reported Ag- and Cu-catalyzed difunctionalization of the isocyano group, involving isocyanide insertion into the N-O bond, for the rapid generation of pyrimidinediones [19] and dihydroquinolinones [20], respectively (Scheme 3b,c). Based on these studies and our continuous interest in N-O bond transformations [21,22,23], we wished to develop more methods regarding isocyanide insertion into N-O bonds for the synthesis of N-containing compounds. Herein, we describe a Cu-catalyzed method for the rapid assembly of ureas using aryl isocyanides and O-benzoyl hydroxylamines as readily accessible starting materials (Scheme 3d). The reactions occur under mild reaction conditions and generated various new structures of unsymmetric ureas.

## 2. Results and Discussion

### 2.1. Optimization of Reaction Conditions

We initially chose isocyanide (**1a**) and 4-benzoyloxymopholine (**2a**) as the model substrates to optimize the reaction (Table 1). We examined several inorganic bases, including NaOAc, K_2_CO_3_, NaHCO_3_, PhCO_2_Na, and PivONa, which could not promote the reaction at all (entries 1–5). Pleasingly, we found that the reaction could generate urea (**3a**) in the presence of PhONa or *t-*BuONa as the base, and the latter promoted the transformation with 86% yield (entries 6 and 7). These results indicated that the bases were significant for the reaction. CuI and CuBr_2_ also catalyzed the reaction, albeit lower yields were obtained (entries 8 and 9). In addition, the reaction failed with FeCl_2_ as the catalyst (entry 10). Copper salt was essential for the reaction, as no product was formed in the absence of CuOAc (entry 11). Transformation **3a** delivered very low yields with DMSO or MeCN as the solvent (entries 12 and 13). 

### 2.2. Substrate Scope

**Scheme 4:**
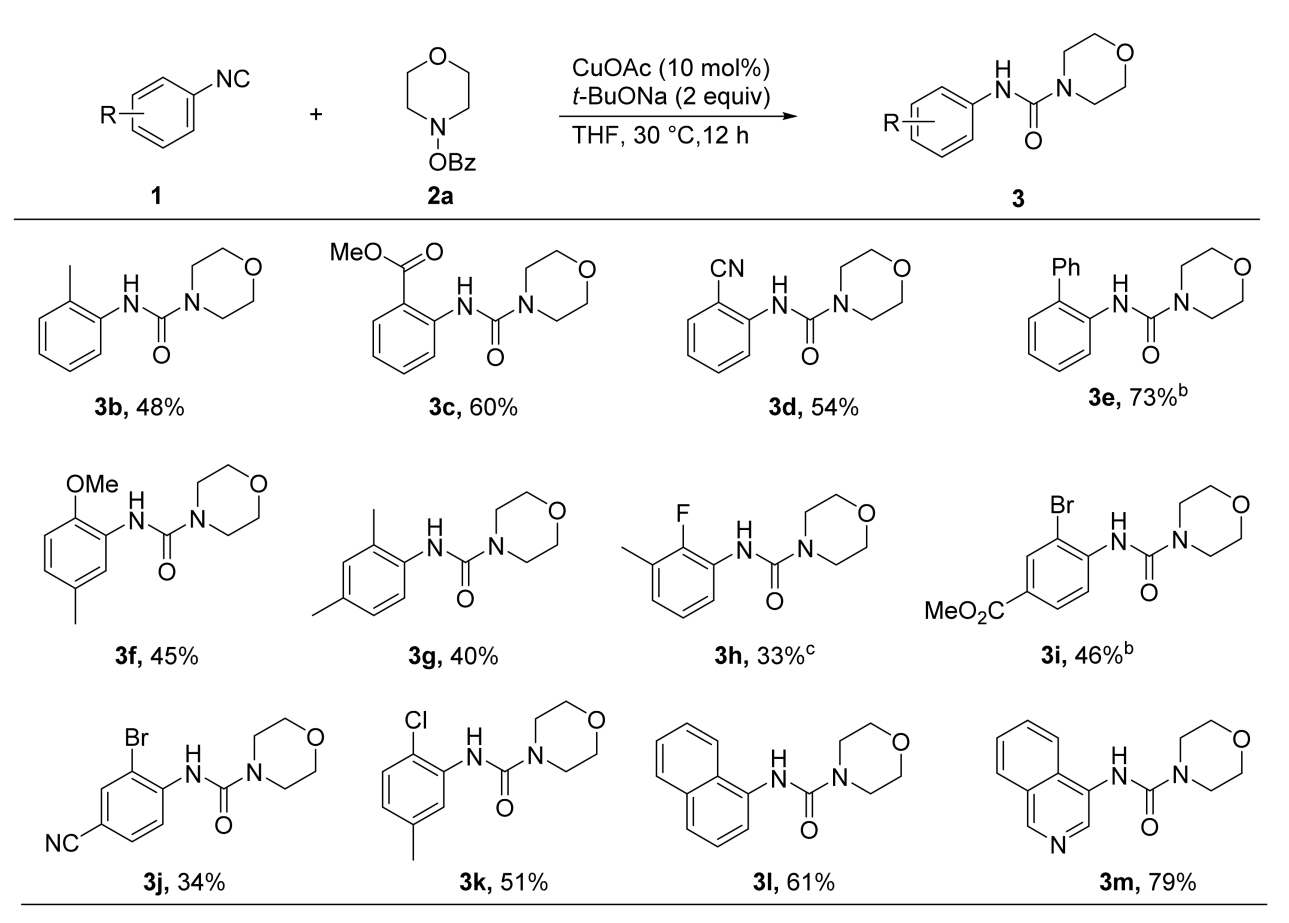
Scope of isocyanides ^a^. ^a^ Reaction conditions: **1** (0.2 mmol), **2a** (1.5 equiv), CuOAc (10 mol%), *t*-BuONa (2 equiv), THF (2 mL), 30 °C, 12 h, under N_2_. Yields are those of the isolated products. ^b^ PhONa (2 equiv) was used instead of *t*-BuONa. ^c^ Reaction was run at 50 °C.

With the optimized reaction conditions in hand, we next probed the substrate scope with regard to the isocyanides (Scheme 4). A wide range of aryl isocyanides were amenable to this method, and a series of functional groups were tolerated, including methyl (**3b**, **3g**, **3h**, and **3k**), ester (**3c** and **3i**), cyano (**3d** and **3j**), phenyl (**3e**), methoxy (**3f**), fluoro (**3h**), bromo (**3i** and **3j**), and chloro groups (**3k**). Moreover, isocyanides bearing naphthyl (**3l**) and isoquinoline (**3m**) moieties also took part in the reaction to deliver the corresponding products with 61% and 79% yield, respectively. For reactions producing ureas in moderate yields, anilines were detected as the main by-products.

**Scheme 5:**
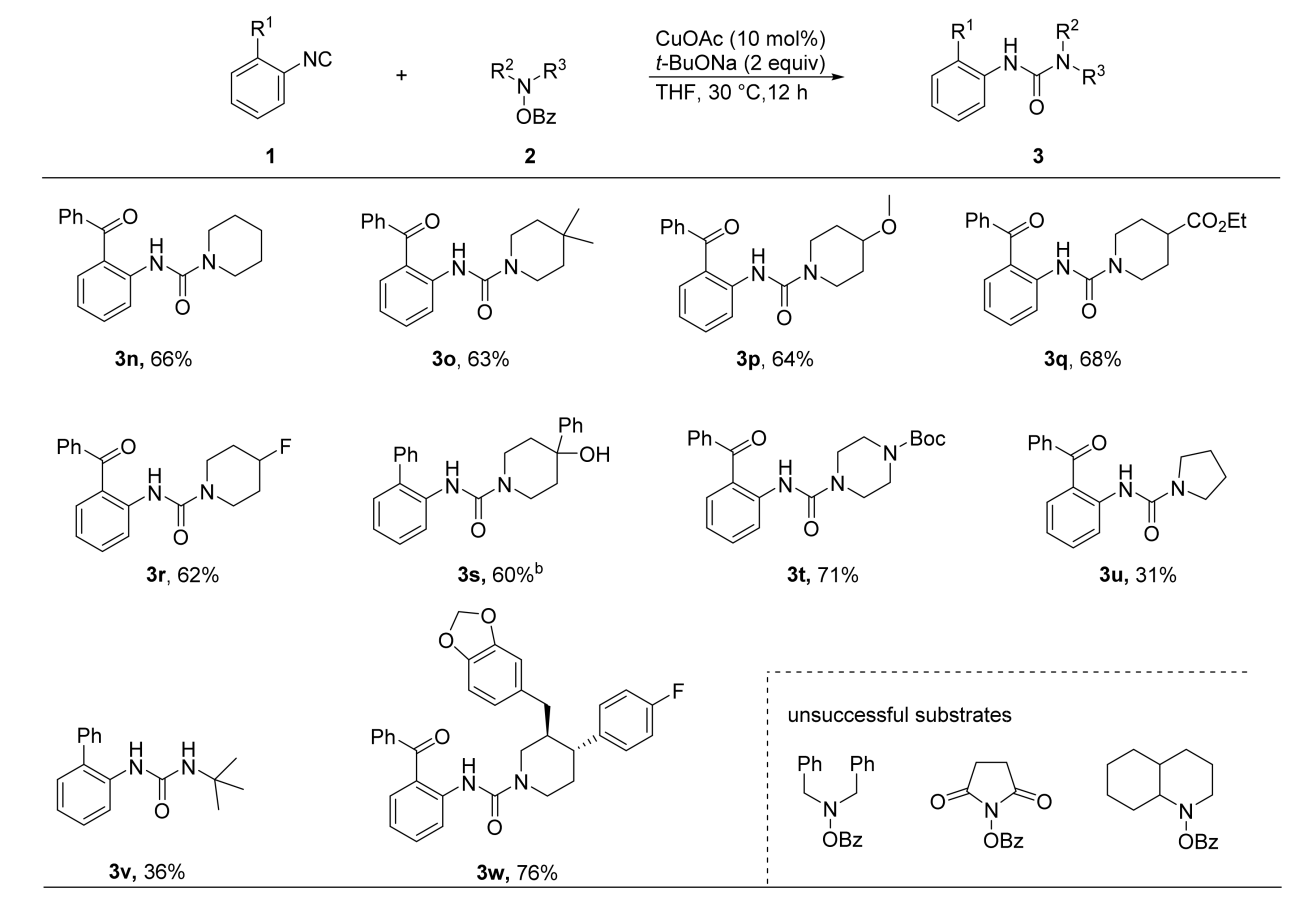
Scope of O-benzoyl hydroxylamines ^a^. ^a^ Reaction conditions: **1** (0.2 mmol), **2** (1.5 equiv), CuOAc (10 mol%), *t*-BuONa (2 equiv), THF (2 mL), 30 °C, 12 h, under N_2_. Yields are those of the isolated products. ^b^ PhONa (2 equiv) was used instead of *t*-BuONa.

Subsequently, we evaluated the scope of the *O*-benzoyl hydroxylamines. As depicted in Scheme 5, *O*-benzoyl hydroxylamines derived from piperidine (**3n**) and substituted piperidines with methyl (**3o**), methoxy (**3p**), ester (**3q**), fluoro (**3r**), and hydroxyl (**3s**) functionalities reacted with isocyanides to produce the target products in moderate yields. In addition, *O*-benzoyl hydroxylamines containing piperazine (**3t**) and pyrrolidine (**3u**) units were also suitable for this transformation. In addition to cyclic secondary amine-derived *O*-benzoyl hydroxylamines, primary amine-derived *O*-benzoyl hydroxylamines participated in the reaction, affording **3v** in a synthetically useful yield. Notably, this method was suitable for hydroxylamine derived from paroxetine (**3w**), a drug used to treat major depressive disorder and obsessive-compulsive disorder. Nevertheless, hydroxylamines derived from other secondary amines, such as dibenzylamine, succinimide, and decahydroquinoline, failed to participate in the transformation.

**Scheme 6:**
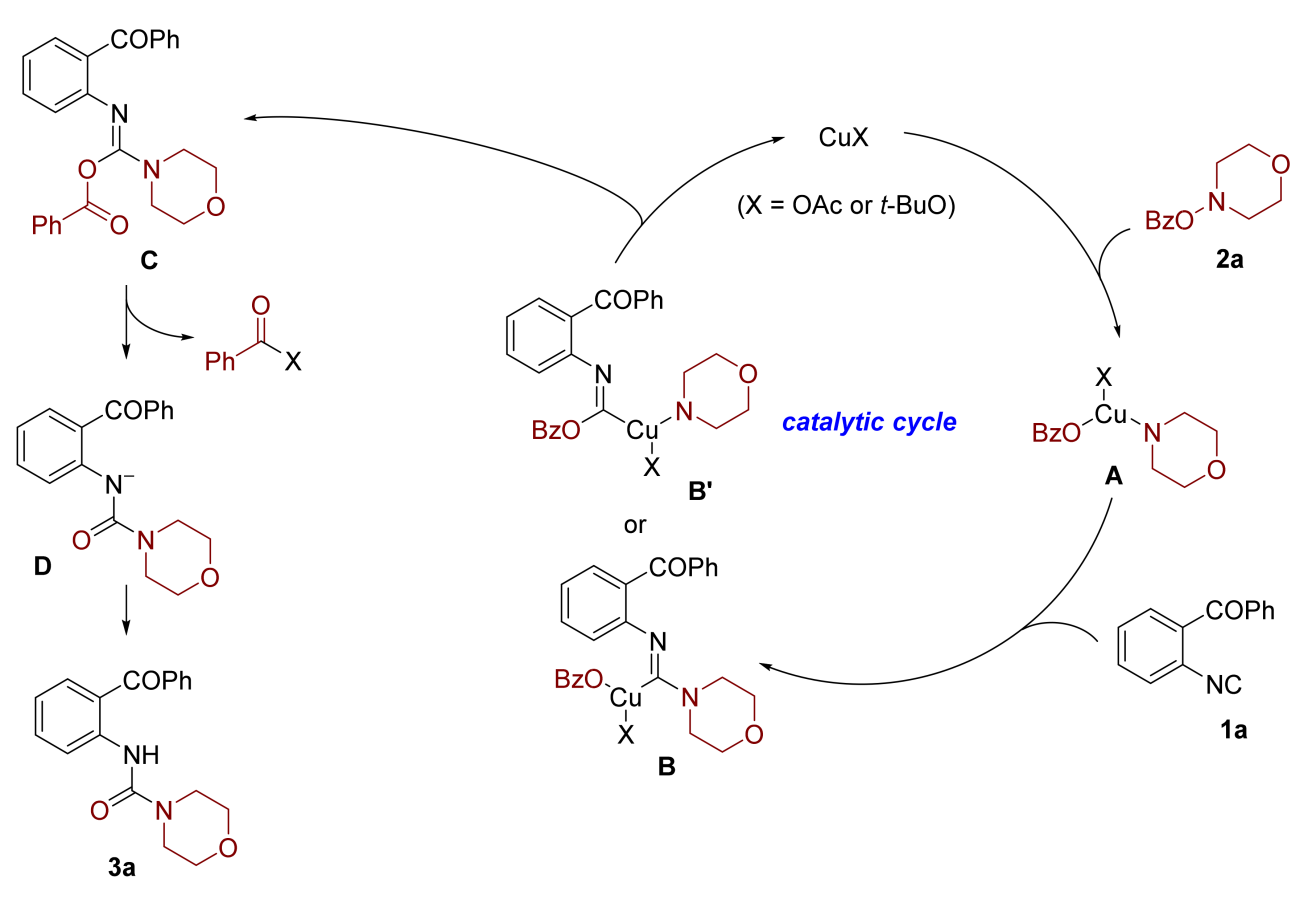
Hypothesis for Cu-catalyzed urea synthesis.

We proposed a reaction mechanism for the reaction of **1a** with **2a**, based on the experimental results and some previous work related to Cu-mediated amination reactions with *O*-benzoyl hydroxylamines as the aminating source [24,25,26,27,28]. As displayed in Scheme 6, first, Cu(I) salt reacted with **2a** to form the aminocopper intermediate **A** through oxidative addition, which then reacted with **1a** to produce copper species **B** or **B’** via isocyanide insertion into the Cu-N or Cu-O bond, respectively. Subsequently, C-O or C-N reductive elimination delivered imidate **C**, which would undergo Mumm-type rearrangement to generate species **D** and an acyl cation [29]. The final product **3a** was formed after protonation during quenching of the reaction. Notably, the acyl cation could be trapped by AcO^−^ or *t*-BuO^−^ to yield the corresponding anhydride or ester, which was observed by GC-MS analysis.

## 3. Materials and Methods

All reactions dealing with air- and moisture-sensitive compounds were carried out in dry reaction vessels under a nitrogen atmosphere. ^1^H and ^13^C nuclear magnetic resonance (NMR) spectra were recorded on a Bruker 600 MHz NMR spectrometer. ^1^H and ^13^C NMR spectra are reported in parts per million (ppm) downfield from an internal standard (Supplementary Materials), tetramethylsilane (0 ppm) and CHCl_3_ (77.0 ppm), respectively. HRMS (*m/z*) was recorded using ESI (Q-TOF) mode. Melting points were determined using a capillary melting point apparatus and are uncorrected. Unless otherwise noted, materials were purchased from commercial suppliers and were used as received. Anhydrous tetrahydrofuran was distilled over Na and stored under N_2_.

### 3.1. Preparation of Substrates

All *O*-benzoyl hydroxylamines and isocyanides were synthesized according to the literature [20].

### 3.2. Preparation of Isocyanides (**1**)

*N*-Formylation

Acetyl formyl anhydride (prepared by stirring 2.5 equiv acetic anhydride and 2.5 equiv formic acid at 55 °C for 2 h) was added dropwise at 0 °C to a stirred solution of aniline (9 mmol) in THF (15 mL), and the mixture was stirred for 2 h at room temperature. Then, the saturated solution of NaHCO_3_ was added, and the aqueous phase was extracted with ethyl acetate. The organic layer was dried over mgSO_4_ and concentrated by rotary evaporation. The residue was purified by column chromatography (petroleum ether/ethyl acetate = 3:1) to give the N-formylated products.

Dehydration

To a solution of the N-formylated products (5 mmol) and Et_3_N (2.1 mL, 15 mmol) in CH_2_Cl_2_ (10 mL) at 0 °C was added triphosgene (0.74 g, 2.5 mmol) in CH_2_Cl_2_ (10 mL). The solution was stirred at 0 °C for 3 h. Then, methanol was added to the suspension solution, and the solution was concentrated by rotary evaporation. The residue was purified by column chromatography (petroleum ether) to give the isocyanides.

### 3.3. Preparation of O-Benzoyl Hydroxylamines (**2**)

A 100 mL flask was charged with benzoyl peroxide (2 mmol), dipotassium hydrogen phosphate (3 mmol), and N,N′-dimethylformamide. The amine starting material (3 mmol) was added dropwise at room temperature. The suspension was stirred at ambient temperature for the indicated reaction time. The reaction was quenched with water (10 mL), and the contents were vigorously stirred for several minutes until all solids were dissolved. The reaction mixture was extracted with ethyl acetate (3 × 30 mL). The organic phase was collected and washed with two 25 mL portions of saturated aq. NaHCO_3_ solution, followed by 25 mL of brine, and then dried over anhydrous Na_2_SO_4_, filtered, and concentrated under vacuum. The residue was purified by column chromatography on silica gel to give the desired product.

### 3.4. General Procedures for Ureas

A 10 mL Schlenk tube equipped with a stir bar was charged with isocyanides (0.2 mmol), *O*-benzoyl hydroxylamines (0.3 mmol), CuOAc (10 mol%), and *t*-BuONa (0.4 mmol). Then, the Schlenk tube was quickly evacuated and refilled with N_2_ three times, followed by the addition of THF (2 mL). The Schlenk tube was sealed with a Teflon screwcap under N_2_ flow, and the reaction mixture was stirred at 30 °C for 12 h. Upon cooling to room temperature, the reaction mixture was diluted with 10 mL of ethyl acetate and filtered through a pad of silica gel, followed by washing the silica gel pad with ethyl acetate (20 mL). Subsequently, the filtrate was concentrated under reduced pressure. The residue was purified by flash chromatography on silica gel to afford the desired products.

*N*-(2-benzoylphenyl)morpholine-4-carboxamide (**3a**)

According to the general procedure, a mixture containing isocyanides (0.2 mmol, 41.4 mg), *O*-benzoyl hydroxylamines (0.3 mmol, 62.1 mg), CuOAc (0.1 mmol, 2.5 mg), *t*-BuONa (0.4 mmol, 38.4 mg), and THF (2 mL) under a nitrogen atmosphere was stirred at 30 °C for 12 h to afford **3a** (53.3 mg). Yellow oil (86% yield, eluent = pentane/ethyl acetate = 3:1); **^1^H NMR** (600 MHz, CDCl_3_): δ 10.91 (s, 1H), 8.56–8.54 (m, 1H), 7.67–7.64 (m, 2H), 7.60–7.54 (m, 3H), 7.50–7.47 (m, 2H), 7.00–6.96 (m, 1H), 3.76 (t, *J* = 6.0 Hz, 4H), 3.60 (t, *J* = 6.0 Hz, 4H); **^13^C NMR** (151 MHz, CDCl_3_): δ 199.7, 153.8, 142.0, 138.1, 133.8, 133.2, 131.1, 128.6, 127.3, 120.9, 119.5, 119.4, 65.6, 43.0; **HRMS** (ESI): calculated for C_18_H_19_N_2_O_3_ [M + H]^+^ 311.1390 found 311.1388.

*N*-(o-tolyl)morpholine-4-carboxamide (**3b**)

According to the general procedure, a mixture containing isocyanides (0.2 mmol, 23.4 mg), *O*-benzoyl hydroxylamines (0.3 mmol, 62.1 mg), CuOAc (0.1 mmol, 2.5 mg), *t*-BuONa (0.4 mmol, 38.4 mg), and THF (2 mL) under a nitrogen atmosphere was stirred at 30 °C for 12 h to afford **3b** (21.1 mg). White solid (48% yield, eluent = pentane/ethyl acetate = 3:1); Mp = 152–153 °C; **^1^H NMR** (600 MHz, CDCl_3_): δ 7.55 (t, *J* = 8.5 Hz, 1H), 7.17 (dd, *J* = 13.1, 7.3 Hz, 2H), 7.03 (t, *J* = 7.4 Hz, 1H), 6.18 (s, 1H), 3.72–3.70 (m, 4H), 3.47–3.41 (m, 4H), 2.23 (s, 3H); **^13^C NMR** (151 MHz, CDCl_3_): δ 155.6, 136.8, 130.4, 129.5, 126.7, 124.5, 123.3, 66.5, 44.4, 17.7; **HRMS** (ESI): calculated for C_12_H_17_N_2_O_2_ [M + H]^+^ 221.1285 found 221.1285.

Methyl 2-(morpholine-4-carboxamido)benzoate (**3c**)

According to the general procedure, a mixture containing isocyanides (0.2 mmol, 32.2 mg), *O*-benzoyl hydroxylamines (0.3 mmol, 62.1 mg), CuOAc (0.1 mmol, 2.5 mg), *t*-BuONa (0.4 mmol, 38.4 mg), and THF (2 mL) under a nitrogen atmosphere was stirred at 30 °C for 12 h to afford **3c** (31.7 mg). White solid (60% yield, eluent = pentane/ethyl acetate = 1:3); Mp = 121–123 °C; **^1^H NMR** (600 MHz, CDCl_3_)**:** δ 8.81 (s, 1H), 8.18–8.14 (m, 1H), 7.39 (t, *J* = 7.5 Hz, 1H), 7.18 (d, *J* = 7.4 Hz, 1H), 7.00 (t, *J* = 7.4 Hz, 1H), 3.74–3.70 (m, 7H), 3.48–3.47 (m, 4H); **^13^C NMR** (151 MHz, CDCl_3_)**:**δ 170.2, 154.7, 139.3, 131.3, 127.6, 122.2, 121.6, 121.5, 66.9, 66.5, 44.1; **HRMS** (ESI): calculated for C_13_H_17_N_2_O_4_ [M + H]^+^ 265.1183 found 265.1183.

*N*-(2-cyanophenyl)morpholine-4-carboxamide (**3d**)

According to the general procedure, a mixture containing isocyanides (0.2 mmol, 25.6 mg), *O*-benzoyl hydroxylamines (0.3 mmol, 62.1 mg), CuOAc (0.1 mmol, 2.5 mg), *t*-BuONa (0.4 mmol, 38.4 mg), and THF (2 mL) under a nitrogen atmosphere was stirred at 30 °C for 12 h to afford **3d** (24.9 mg). Yellow solid (54% yield, eluent = pentane/ethyl acetate = 3:1); Mp = 162–163 °C; **^1^H NMR** (600 MHz, CDCl_3_): δ 8.25 (d, *J* = 8.5 Hz, 1H), 7.58–7.50 (m, 2H), 7.09 (t, *J* = 7.6 Hz, 1H), 6.98 (s, 1H), 3.77–3.76 (m, 4H), 3.54–3.53 (m, 4H); **^13^C NMR** (151 MHz, CDCl_3_): δ 153.7, 142.0, 134.2, 131.8, 122.8, 120.5, 116.9, 101.2, 66.4, 44.3; **HRMS** (ESI): calculated for C_12_H_14_N_3_O_2_ [M + H]^+^ 232.1081 found 232.1082.

*N*-([1,1′-biphenyl]-2-yl)morpholine-4-carboxamide (**3e**)

According to the general procedure, a mixture containing isocyanides (0.2 mmol, 35.8 mg), *O*-benzoyl hydroxylamines (0.3 mmol, 62.1 mg), CuOAc (0.1 mmol, 2.5 mg), PhONa (0.4 mmol, 46.4 mg), and THF (2 mL) under a nitrogen atmosphere was stirred at 30 °C for 12 h to afford **3e** (41.2 mg). White solid (73% yield, eluent = pentane/ethyl acetate = 3:1); Mp = 118–119 °C;**^1^H NMR** (600 MHz, CDCl_3_): δ 8.10 (d, *J* = 8.2 Hz, 1H), 7.48 (t, *J* = 7.5 Hz, 2H), 7.42 –7.33 (m, 4H), 7.22 (dd, *J* = 7.5, 1.1 Hz, 1H), 7.11 (t, *J* = 7.5 Hz, 1H), 6.47 (s, 1H), 3.61 (t, *J* = 6.0 Hz, 4H), 3.22 (t, *J* = 6.0 Hz, 4H); **^13^C NMR** (151 MHz, CDCl_3_): δ 154.8, 138.6, 135.8, 131.9, 129.7, 129.3, 129.1, 128.5, 128.0, 123.1, 121.0, 66.4, 44.1; **HRMS** (ESI): calculated for C_17_H_19_N_2_O_2_ [M + H]^+^ 283.1441 found 283.1439.

*N*-(2-methoxy-5-methylphenyl)morpholine-4-carboxamide (**3f**)

According to the general procedure, a mixture containing isocyanides (0.2 mmol, 29.4 mg), *O*-benzoyl hydroxylamines (0.3 mmol, 62.1 mg), CuOAc (0.1 mmol, 2.5 mg), *t*-BuONa (0.4 mmol, 38.4 mg), and THF (2 mL) under a nitrogen atmosphere was stirred at 30 °C for 12 h to afford **3f** (22.5 mg). Yellow oil, (45% yield, eluent = pentane/ethyl acetate = 3:1); **^1^H NMR** (600 MHz, CDCl_3_): δ 7.99 (s, 1H), 7.05 (s, 1H), 6.75 (m, 2H), 3.84 (s, 3H), 3.74 (t, *J* = 6.0 Hz, 4H), 3.49 (t, *J* = 6.0 Hz, 4H), 2.29 (s, 3H); **^13^C NMR** (151 MHz, CDCl_3_): δ 154.8, 145.7, 130.7, 128.2, 122.4, 119.8, 109.7, 66.5, 55.9, 44.2, 21.0; **HRMS** (ESI): calculated for C_13_H_19_N_2_O_3_ [M + H]^+^ 251.1390 found 251.1389.

*N*-(2,4-dimethylphenyl)morpholine-4-carboxamide (**3g**)

According to the general procedure, a mixture containing isocyanides (0.2 mmol, 26.2 mg), *O*-benzoyl hydroxylamines (0.3 mmol, 62.1 mg), CuOAc (0.1 mmol, 2.5 mg), *t*-BuONa (0.4 mmol, 38.4 mg), and THF (2 mL) under a nitrogen atmosphere was stirred at 30 °C for 12 h to afford **3g** (18.7 mg). Yellow solid (40% yield, eluent = pentane/ethyl acetate = 3:1); Mp = 137–138 °C; **^1^H NMR** (600 MHz, CDCl_3_): δ 7.37 (d, *J* = 8.5 Hz, 1H), 7.00–6.95 (m, 2H), 6.09 (s, 1H), 3.71 (t, *J* = 6.0 Hz, 4H), 3.43 (t, *J* = 6.0 Hz, 4H), 2.28 (s, 3H), 2.19 (s, 3H); **^13^C NMR** (151 MHz, CDCl_3_): δ 155.9, 134.3, 134.0, 131.1, 129.9, 127.3, 123.8, 66.5, 44.4, 20.8, 17.7; **HRMS** (ESI): calculated for C_13_H_19_N_2_O_2_ [M + H]^+^ 235.1441 found 235.1448.

*N*-(2-fluoro-3-methylphenyl)morpholine-4-carboxamide (**3h**)

According to the general procedure, a mixture containing isocyanides (0.2 mmol, 27.0 mg), *O*-benzoyl hydroxylamines (0.3 mmol, 62.1 mg), CuOAc (0.1 mmol, 2.5 mg), *t*-BuONa (0.4 mmol, 38.4 mg), and THF (2 mL) under a nitrogen atmosphere was stirred at 50 °C for 12 h to afford **3h** (15.7 mg). Yellow solid (33% yield, eluent = pentane/ethyl acetate = 3:1); Mp = 109–110 °C; **^1^H NMR** (600 MHz, CDCl_3_): δ 7.88 (t, *J* = 7.8 Hz, 1H), 6.98 (t, *J* = 7.9 Hz, 1H), 6.83 (t, *J* = 7.4 Hz, 1H), 6.57 (s, 1H), 3.75 (t, *J* = 4.8 Hz, 4H), 3.50 (t, *J* = 4.8 Hz, 4H), 2.26 (d, *J* = 1.8 Hz, 3H); **^13^C NMR** (151 MHz, CDCl_3_): δ 154.5, 151.3(d, ^1^*J_C-F_* = 238.6Hz), 127.0(d, ^2^*J_C-F_* = 10.6Hz), 124.8(d, ^3^*J_C-F_* = 6.0Hz), 124.2(d, ^2^*J_C-F_* = 16.6Hz), 123.8(d, ^3^*J_C-F_* = 4.5Hz), 118.9, 66.5, 44.3, 14.4(d, ^3^*J_C-F_* = 4.5Hz); **^19^F NMR** (565 MHz, CDCl_3_): δ -137.2; **HRMS** (ESI): calculated for C_12_H_16_FN_2_O_2_ [M + H]^+^ 239.1190 found 239.1191.

Methyl 3-bromo-4-(piperidine-1-carboxamido)benzoate (**3i**)

According to the general procedure, a mixture containing isocyanides (0.2 mmol, 47.8 mg), *O*-benzoyl hydroxylamines (0.3 mmol, 62.1 mg), CuOAc (0.1 mmol, 2.5 mg), PhONa (0.4 mmol, 46.4 mg), and THF (2 mL) under a nitrogen atmosphere was stirred at 30 °C for 12 h to afford **3i** (31.5 mg). White solid (46% yield, eluent = pentane/ethyl acetate = 3:1); Mp = 121–122 °C; **^1^H NMR** (600 MHz, CDCl_3_): δ 8.33 (dd, *J* = 11.8, 6.9 Hz, 1H), 8.21–8.15 (m, 1H), 7.95 (dd, *J* = 12.2, 4.8 Hz, 1H), 7.25 (s, 1H), 3.88 (s, 3H), 3.79–3.72 (m, 4H), 3.55–3.49 (m, 4H); **^13^C NMR** (151 MHz, CDCl_3_): δ 165.5, 153.6, 140.7, 133.5, 130.0, 125.2, 119.4, 112.1, 66.4, 52.1, 44.3; **HRMS** (ESI): calculated for C_13_H_16_BrN_2_O_4_ [M + H]^+^ 343.0288 found 343.0287.

*N*-(2-bromo-4-cyanophenyl)morpholine-4-carboxamide (**3j**)

According to the general procedure, a mixture containing isocyanides (0.2 mmol, 41.2 mg), *O*-benzoyl hydroxylamines (0.3 mmol, 62.1 mg), CuOAc (0.1 mmol, 2.5 mg), *t*-BuONa (0.4 mmol, 38.4 mg), and THF (2 mL) under a nitrogen atmosphere was stirred at 30 °C for 12 h to afford **3j** (21.0 mg). White solid (34% yield, eluent = pentane/ethyl acetate = 3:1); Mp = 121–122 °C; **^1^H NMR** (600 MHz, CDCl_3_): δ 8.42 (d, *J* = 8.7 Hz, 1H), 7.79 (d, *J* = 1.8 Hz, 1H), 7.57 (dd, *J* = 8.7, 1.7 Hz, 1H), 7.27 (s, 1H), 3.78 (t, *J* = 6.0 Hz, 4H), 3.54 (t, *J* = 6.0 Hz, 4H); **^13^C NMR** (151 MHz, CDCl_3_): δ 153.2, 141.0, 135.4, 132.5, 120.1, 117.6, 112.1, 106.6, 66.3, 44.3; **HRMS** (ESI): [M + K]^+^ calculated for C_12_H_12_BrKN_3_O_2_ 347.9744 found 347.9741.

*N*-(2-chloro-5-methylphenyl)morpholine-4-carboxamide (**3k**)

According to the general procedure, a mixture containing isocyanides (0.2 mmol, 30.2 mg), *O*-benzoyl hydroxylamines (0.3 mmol, 62.1 mg), CuOAc (0.1 mmol, 2.5 mg), *t*-BuONa (0.4 mmol, 38.4 mg), and THF (2 mL) under a nitrogen atmosphere was stirred at 30 °C for 12 h to afford **3k** (25.9 mg). Yellow solid (51% yield, eluent = pentane/ethyl acetate = 3:1); Mp = 106–107 °C; **^1^H NMR** (600 MHz, CDCl_3_): δ 8.02 (d, *J* = 1.2 Hz, 1H), 7.20 (d, *J* = 8.2 Hz, 1H), 6.93 (s, 1H), 6.78 (dd, *J* = 8.1, 1.4 Hz, 1H), 3.76 (t, *J* = 6.0 Hz, 4H), 3.51 (t, *J* = 6.0 Hz, 4H), 2.32 (s, 3H); **^13^C NMR** (151 MHz, CDCl_3_): δ 154.3, 137.9, 135.1, 128.3, 124.2, 121.4, 119.4, 66.4, 44.2, 21.3; **HRMS** (ESI): calculated for C_12_H_16_ClN_2_O_2_ [M + H]^+^ 255.0895 found 255.0893.

*N*-(naphthalen-1-yl)morpholine-4-carboxamide (**3l**)

According to the general procedure, a mixture containing isocyanides (0.2 mmol, 30.6 mg), *O*-benzoyl hydroxylamines (0.3 mmol, 62.1 mg), CuOAc (0.1 mmol, 2.5 mg), *t*-BuONa (0.4 mmol, 38.4 mg), and THF (2 mL) under a nitrogen atmosphere was stirred at 30 °C for 12 h to afford **3l** (31.2 mg). Yellow solid (61% yield, eluent = pentane/ethyl acetate = 3:1); Mp = 192–193 °C; **^1^H NMR** (600 MHz, CDCl_3_): δ 7.91–7.80 (m, 2H), 7.68 (d, *J* = 8.2 Hz, 1H), 7.60 (d, *J* = 7.3 Hz, 1H), 7.53–7.47 (m, 2H), 7.44 (t, *J* = 7.8 Hz, 4H), 6.69 (s, 1H), 3.68 (t, *J* = 6.0 Hz, 4H), 3.45 (t, *J* = 6.0 Hz, 4H); **^13^C NMR** (151 MHz, CDCl_3_): δ 156.2, 134.3, 133.8, 128.7, 128.3, 126.1, 125.9, 125.7, 125.4, 121.3, 121.2, 66.5, 44.5; **HRMS** (ESI): calculated for C_15_H_17_N_2_O_3_ [M + H]^+^ 257.1285 found 257.1283.

*N*-(isoquinolin-4-yl)morpholine-4-carboxamide (**3m**)

According to the general procedure, a mixture containing isocyanides (0.2 mmol, 30.8 mg), *O*-benzoyl hydroxylamines (0.3 mmol, 62.1 mg), CuOAc (0.1 mmol, 2.5 mg), *t*-BuONa (0.4 mmol, 38.4 mg), and THF (2 mL) under a nitrogen atmosphere was stirred at 30 °C for 12 h to afford **3m** (40.6 mg). Yellow solid (79% yield, eluent = pentane/ethyl acetate = 1:6); Mp = 176–177 °C; **^1^H NMR** (600 MHz, CDCl_3_): δ 8.99 (s, 1H), 8.43 (s, 1H), 7.91 (d, *J* = 8.1 Hz, 1H), 7.74 (d, *J* = 8.4 Hz, 1H), 7.64 (t, *J* = 7.6 Hz, 1H), 7.56 (t, *J* = 7.5 Hz, 1H), 7.12 (s, 1H), 3.63 (t, *J* = 6.0 Hz, 4H), 3.42 (t, *J* = 6.0 Hz, 4H); **^13^C NMR** (151 MHz, CDCl_3_): δ 156.1, 149.8, 139.0, 131.8, 130.3, 129.4, 128.8, 127.8, 127.3, 121.5, 66.5, 44.4; **HRMS** (ESI): calculated for C_14_H_16_N_3_O_2_ [M + H]^+^ 258.1237 found 258.1237.

*N*-(2-benzoylphenyl)piperidine-1-carboxamide (**3n**)

According to the general procedure, a mixture containing isocyanides (0.2 mmol, 41.4 mg), *O*-benzoyl hydroxylamines (0.3 mmol, 61.5), CuOAc (0.1 mmol, 2.5 mg), *t*-BuONa (0.4 mmol, 38.4 mg), and THF (2 mL) under a nitrogen atmosphere was stirred at 30 °C for 12 h to afford **3n** (40.7 mg). Yellow oil (66% yield, eluent = pentane/ethyl acetate = 3:1); **^1^H NMR** (600 MHz, CDCl_3_): δ 10.82 (s, 1H), 8.53 (d, *J* = 8.9 Hz, 1H), 7.66 (d, *J* = 7.6 Hz, 2H), 7.58–7.52 (m, 3H), 7.47 (t, *J* = 7.6 Hz, 2H), 6.93 (t, *J* = 7.6 Hz, 1H), 3.59–3.53 (m, 4H), 1.70–1.60 (m, 6H); **^13^C NMR** (151 MHz, CDCl_3_): δ 199.5, 153.6, 142.5, 138.4, 133.6, 133.1, 130.9, 128.6, 127.2, 120.8, 119.6, 118.9, 44.1, 24.8, 23.5; **HRMS** (ESI): calculated for C_19_H_21_N_2_O_2_ [M + H]^+^ 309.1598 found 309.1595.

*N*-(2-benzoylphenyl)-4,4-dimethylpiperidine-1-carboxamide (**3o**)

According to the general procedure, a mixture containing isocyanides (0.2 mmol, 41.4 mg), *O*-benzoyl hydroxylamines (0.3 mmol, 70.0 mg), CuOAc (0.1 mmol, 2.5 mg), *t*-BuONa (0.4 mmol, 38.4 mg), and THF (2 mL) under a nitrogen atmosphere was stirred at 30 °C for 12 h to afford **3o** (42.3 mg). Yellow oil (63% yield, eluent = pentane/ethyl acetate = 3:1); **^1^H NMR** (600 MHz, CDCl_3_): δ 10.84 (s, 1H), 8.53 (d, *J* = 9.0 Hz, 1H), 7.66 (d, *J* = 7.4 Hz, 2H), 7.58 (t, *J* = 7.4 Hz, 1H), 7.54 (dd, *J* = 7.4, 5.8 Hz, 2H), 7.48 (t, *J* = 7.6 Hz, 2H), 6.94 (t, *J* = 7.6 Hz, 1H), 3.57 (t, *J* = 5.7 Hz, 4H), 1.45 (t, *J* = 5.8Hz, 4H), 0.99 (s, 6H); **^13^C NMR** (151 MHz, CDCl_3_): δ 200.5, 154.7, 143.5, 139.3, 134.6, 134.1, 132.0, 129.6, 128.2, 121.8, 120.6, 120.0, 40.7, 38.4, 28.9, 27.7.

*N*-(2-benzoylphenyl)-4-methoxypiperidine-1-carboxamide (**3p**)

According to the general procedure, a mixture containing isocyanides (0.2 mmol, 41.4 mg), *O*-benzoyl hydroxylamines (0.3 mmol, 70.5 mg), CuOAc (0.1 mmol, 2.5 mg), *t*-BuONa (0.4 mmol, 38.4 mg), and THF (2 mL) under a nitrogen atmosphere was stirred at 30 °C for 12 h to afford **3p** (43.3 mg). Yellow oil (64% yield, eluent = pentane/ethyl acetate = 3:1); **^1^H NMR** (600 MHz, CDCl_3_): δ 10.89 (s, 1H), 8.52 (d, *J* = 8.9 Hz, 1H), 7.66 (d, *J* = 7.3 Hz, 2H), 7.58 (t, *J* = 7.4 Hz, 1H), 7.55–7.52 (m, 2H), 7.48 (t, *J* = 7.7 Hz, 2H), 6.95 (t, *J* = 7.6 Hz, 1H), 3.90–3.85 (m, 2H), 3.47–3.43 (m, 1H), 3.38–3.34 (m, 5H), 1.94 (ddd, *J* = 12.6, 6.9, 3.4 Hz, 2H), 1.69–1.67 (m, 1H), 1.66–1.63 (m, 1H); **^13^C NMR** (151 MHz, CDCl_3_): δ 200.6, 154.5, 143.4, 139.3, 134.7, 134.1, 132.0, 129.6, 128.3, 121.8, 120.6, 120.1, 75.5, 55.7, 41.3, 30.5; **HRMS** (ESI): calculated for C_20_H_22_N_2_NaO_3_ [M + Na]^+^ 361.1523 found 361.1529.

Ethyl 1-((2-benzoylphenyl)carbamoyl)piperidine-4-carboxylate (**3q**)

According to the general procedure, a mixture containing isocyanides (0.2 mmol, 41.4 mg), *O*-benzoyl hydroxylamines (0.3 mmol, 78.9 mg), CuOAc (0.1 mmol, 2.5 mg), *t*-BuONa (0.4 mmol, 38.4 mg), and THF (2 mL) under a nitrogen atmosphere was stirred at 30 °C for 12 h to afford **3q** (51.7 mg). Yellow oil (68% yield, eluent = pentane/ethyl acetate = 3:1); **^1^H NMR** (600 MHz, CDCl_3_): δ 10.89 (s, 1H), 8.52 (dd, *J* = 8.9, 0.8 Hz, 1H), 7.69–7.62 (m, 2H), 7.60–7.51 (m, 3H), 7.48 (t, *J* = 7.7 Hz, 2H), 6.99–6.92 (m, 1H), 4.20–4.11 (m, 4H), 3.13–3.05 (m, 2H), 2.53 (tt, *J* = 10.7, 4.0 Hz, 1H), 2.02–1.99 (m, 2H), 1.81–1.75 (m, 2H), 1.25 (t, *J* = 7.1 Hz, 3H); **^13^C NMR** (151 MHz, CDCl_3_): δ 199.6, 173.2, 153.5, 142.3, 138.2, 133.7, 133.1, 131.0, 128.6, 127.3, 120.8, 119.6, 119.2, 59.6, 42.4, 40.0, 27.0, 13.2; **HRMS** (ESI): calculated for C_22_H_25_N_2_O_4_ [M + H]^+^ 381.1809 found 381.1806.

*N*-(2-benzoylphenyl)-4-fluoropiperidine-1-carboxamide (**3r**)

According to the general procedure, a mixture containing isocyanides (0.2 mmol, 41.4 mg), *O*-benzoyl hydroxylamines (0.3 mmol, 61.6 mg), CuOAc (0.1 mmol, 2.5 mg), *t*-BuONa (0.4 mmol, 38.4 mg), and THF (2 mL) under a nitrogen atmosphere was stirred at 30 °C for 12 h to afford **3r** (40.4 mg). Yellow oil (62% yield, eluent = pentane/ethyl acetate = 3:1); **^1^H NMR** (600 MHz, CDCl_3_): δ 10.94 (s, 1H), 8.52 (d, *J* = 8.9 Hz, 1H), 7.66 (d, *J* = 7.2 Hz, 2H), 7.60–7.54 (m, 3H), 7.48 (t, *J* = 7.7 Hz, 2H), 6.97 (t, *J* = 7.6 Hz, 1H), 4.95–4.82 (m, 1H), 3.73–3.64 (m, 4H), 1.98–1.91 (m, 4H); **^13^C NMR** (151 MHz, CDCl_3_): δ 200.6, 154.4, 143.2, 139.2, 134.7, 134.2, 132.1, 129.6, 128.3, 121.8, 120.6, 120.3, 87.8 (d, ^1^*J_C-F_* = 172.1Hz), 40.1 (d, ^3^*J_C-F_* = 6.0Hz), 31.1 (d, ^2^*J_C-F_* = 21.1Hz).

*N*-([1,1′-biphenyl]-2-yl)-4-hydroxy-4-phenylpiperidine-1-carboxamide (**3s**)

According to the general procedure, a mixture containing isocyanides (0.2 mmol, 35.8 mg), *O*-benzoyl hydroxylamines (0.3 mmol, 89.1 mg), CuOAc (0.1 mmol, 2.5 mg), PhONa (0.4 mmol, 46.4 mg), and THF (2 mL) under a nitrogen atmosphere was stirred at 30 °C for 12 h to afford **3s** (44.6 mg). White solid (60% yield, eluent = pentane/ethyl acetate = 3:1); Mp = 108–109 °C;**^1^H NMR** (600 MHz, CDCl_3_): δ 8.11 (d, *J* = 8.1 Hz, 1H), 7.48–7.42 (m, 4H), 7.39–7.35 (m, 6H), 7.28 (t, *J* = 7.3 Hz, 1H), 7.22 (d, *J* = 7.5 Hz, 1H), 7.10 (t, *J* = 7.5 Hz, 1H), 6.58 (s, 1H), 3.68 (d, *J* = 13.0 Hz, 2H), 3.27 (td, *J* = 13.1, 2.1 Hz, 2H), 1.94 (td, *J* = 13.4, 4.6 Hz, 2H), 1.79–1.75 (m, 1H), 1.69 (d, *J* = 12.7 Hz, 2H); **^13^C NMR** (151 MHz, CDCl_3_): δ 154.7, 147.6, 138.7, 136.2, 131.7, 129.6, 129.3, 129.1, 128.5(2), 127.9, 127.4, 124.4, 122.8, 120.9, 71.4, 40.5, 38.0; **HRMS** (ESI): calculated for C_24_H_25_N_2_O_2_ [M + H]^+^ 373.1911 found 373.1910.

Tert-butyl 4-((2-benzoylphenyl)carbamoyl)piperazine-1-carboxylate (**3t**)

According to the general procedure, a mixture containing isocyanides (0.2 mmol, 41.4 mg), *O*-benzoyl hydroxylamines (0.3 mmol, 91.8 mg), CuOAc (0.1 mmol, 2.5 mg), *t*-BuONa (0.4 mmol, 38.4 mg), and THF (2 mL) under a nitrogen atmosphere was stirred at 30 °C for 12 h to afford **3t** (58.1 mg). Yellow solid (71% yield, eluent = pentane/ethyl acetate = 3:1); Mp = 106–107 °C; **^1^H NMR** (600 MHz, CDCl_3_): δ 10.92 (s, 1H), 8.56–8.51 (m, 1H), 7.67–7.65 (m, 2H), 7.58–7.54 (m, 3H), 7.48 (t, *J* = 7.7 Hz, 2H), 7.00–6.95 (m, 1H), 3.62–3.58 (m, 4H), 3.54–3.50 (m, 4H), 1.47 (s, 9H); **^13^C NMR** (151 MHz, CDCl_3_): δ 199.7, 153.6(2), 142.0, 138.1, 133.7, 133.2, 131.1, 128.6, 127.3, 120.9, 119.6, 119.4, 79.2, 42.6, 27.4; **HRMS** (ESI): calculated for C_23_H_28_N_3_O_4_ [M + H]^+^ 410.2074 found 410.2074.

*N*-(2-benzoylphenyl)pyrrolidine-1-carboxamide (**3u**)

According to the general procedure, a mixture containing isocyanides (0.2 mmol, 41.4 mg), *O*-benzoyl hydroxylamines (0.3 mmol, 57.3 mg), CuOAc (0.1 mmol, 2.5 mg), *t*-BuONa (0.4 mmol, 38.4 mg), and THF (2 mL) under a nitrogen atmosphere was stirred at 30 °C for 12 h to afford **3u** (18.2 mg). White solid (31% yield, eluent = pentane/ethyl acetate = 3:1); Mp = 111–112 °C; **^1^H NMR** (600 MHz, CDCl_3_): δ 10.61 (s, 1H), 8.63 (d, *J* = 8.8 Hz, 1H), 7.66 (d, *J* = 7.5 Hz, 2H), 7.60–7.50 (m, 3H), 7.48 (t, *J* = 7.6 Hz, 2H), 6.95 (t, *J* = 7.5 Hz, 1H), 3.57 (t, *J* = 6.6 Hz, 4H), 2.03–1.92 (m, 4H). **^13^C NMR** (151 MHz, CDCl_3_): δ 200.4, 153.9, 143.3, 139.4, 134.6, 134.0, 131.9, 129.6, 128.2, 121.6, 120.4, 119.9, 45.8, 25.5. **HRMS** (ESI): calculated for C_18_H_19_N_2_O_2_ [M + H]^+^ 295.1441 found 295.1439.

1-([1,1′-biphenyl]-2-yl)-3-(tert-butyl)urea (**3v**)

According to the general procedure, a mixture containing isocyanides (0.2 mmol, 35.8 mg), *O*-benzoyl hydroxylamines (0.3 mmol,57.9 mg), CuOAc (0.1 mmol, 2.5 mg), *t*-BuONa (0.4 mmol, 38.4 mg), and THF (2 mL) under a nitrogen atmosphere was stirred at 30 ℃ for 12 h to afford **3v** (19.3 mg). White solid (36% yield, eluent = pentane/ethyl acetate = 3:1); Mp = 151–152 °C; **^1^H NMR** (600 MHz, CDCl_3_) δ 7.79 (d, *J* = 7.9 Hz, 1H), 7.45 (t, *J* = 7.5 Hz, 2H), 7.37 (dd, *J* = 11.7, 4.3 Hz, 3H), 7.34–7.30 (m, 1H), 7.26–7.23 (m, 1H), 7.15–7.12 (m, 1H), 5.95 (s, 1H), 4.39 (s, 1H), 1.26 (s, 9H); **^13^C NMR** (151 MHz,CDCl_3_): δ 154.6, 138.8, 135.8, 133.5, 130.5, 129.2, 128.9, 128.5, 127.7, 123.9, 122.8, 50.7, 29.2; **HRMS** (ESI): calculated for C_17_H_21_N_2_O [M + H]^+^ 269.1648 found 269.1647.

(3S,4R)-3-(benzo[d][1,3]dioxol-5-ylmethyl)-N-(2-benzoylphenyl)-4-(4-fluorophenyl)piperidine-1-carboxamide (**3w**)

According to the general procedure, a mixture containing isocyanides (0.2 mmol, 41.4 mg), *O*-benzoyl hydroxylamines (0.3 mmol, 129.9 mg), CuOAc (0.1 mmol, 2.5 mg), *t*-BuONa (0.4 mmol, 38.4 mg), and THF (2 mL) under a nitrogen atmosphere was stirred at 30 ℃ for 12 h to afford **3w** (81.5 mg). Yellow oil (76% yield, eluent = pentane/ethyl acetate = 3:1);**^1^H NMR** (600 MHz, CDCl_3_): δ 11.04 (s, 1H), 8.61–8.58 (m, 1H), 7.70–7.67 (m, 2H), 7.60–7.55 (m, 3H), 7.49 (t, *J* = 7.7 Hz, 2H), 7.15 (dd, *J* = 8.6 Hz, 2H), 6.99–6.96 (m, 3H), 6.61 (d, *J* = 8.5 Hz, 1H), 6.44 (d, *J* = 2.4 Hz, 1H), 6.18 (dd, *J* = 8.5, 2.5 H, 1H), 5.88 (s, 2H), 4.63 (d, *J* = 12.2 Hz, 1H), 4.43 (d, *J* = 13.2 Hz, 1H), 3.70 (dd, *J* = 9.4, 2.7 Hz, 1H), 3.51 (dd, *J* = 9.3, 6.6 Hz, 1H), 3.09–3.01 (m, 2H), 2.79 (td, J = 11.9, 3.8 Hz, 1H), 2.18–2.10 (m, 1H), 1.95–1.93 (m, 1H), 1.87–1.80 (m, 1H); **^13^C NMR** (151 MHz, CDCl_3_): δ 199.5, 160.7(d, ^1^*J_C-F_* = 244.6 Hz), 153.4(d, ^2^*J_C-F_* = 18.1 Hz), 147.2, 142.3, 140.7, 138.2, 137.8(2), 133.6, 133.1, 131.0, 128.7, 127.8(d, ^3^*J_C-F_* = 7.6 Hz), 127.2, 120.8, 119.5, 119.2, 114.5(d, ^2^*J_C-F_* = 21.1 Hz), 106.8, 104.6, 100.1, 97.1, 67.6, 46.7, 43.8, 43.2, 41.2, 32.9.

## 4. Conclusions

In summary, we have developed a new Cu-catalyzed method that enables the synthesis of a series of unsymmetric ureas using aryl isocyanides and *O*-benzoyl hydroxylamines as the readily accessible starting materials. With operational simplicity and good functional group compatibility, this approach has substantially expanded the scope of urea synthesis. The exploration of more insertion reactions involving N-O cleavage is ongoing in our group.

## Figures and Tables

**Table 1:** Optimization for the Cu-catalyzed urea synthesis ^a^.

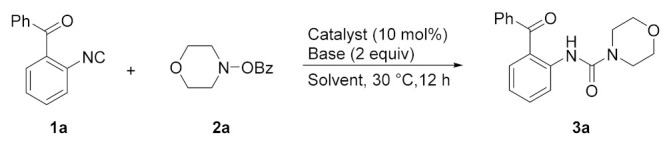				
Entry	Catalyst	Base	Solvent	Yield (%) ^b^
1	CuOAc	NaOAc	THF	0
2	CuOAc	K_2_CO_3_	THF	0
3	CuOAc	NaHCO_3_	THF	0
4	CuOAc	PhCO_2_Na	THF	0
5	CuOAc	PivONa	THF	0
6	CuOAc	PhONa	THF	23
7	CuOAc	*t-*BuONa	THF	86
8	CuI	*t-*BuONa	THF	74
9	CuBr_2_	*t-*BuONa	THF	65
10	FeCl_2_	*t-*BuONa	THF	trace
11	-	*t-*BuONa	THF	0
12	CuOAc	*t-*BuONa	DMSO	messy
13	CuOAc	*t-*BuONa	MeCN	<5

^a^ Reaction conditions unless otherwise indicated: **1a** (0.2 mmol), **2a** (1.5 equiv), catalyst (10 mol%), base (2 equiv), solvent (2 mL), 30 °C, 12 h, under N_2_. ^b^ Isolated yields.

## Data Availability

The data obtained by this study are included in this article and in the Supplementary Materials.

## Supplementary Materials

The following supporting information can be downloaded at: https://www.mdpi.com/article/10.3390/molecules27238219/s1. All experimental data, detailed experimental procedures, and ^1^H NMR, ^13^C NMR, ^19^F NMR spectral are available online.

## Sample Availability

Samples of the compounds are not available from the authors.
