# Supplementary material for: Urea Synthesis from Isocyanides and O-Benzoyl Hydroxylamines Catalyzed by a Copper Salt
Source: Molecules. 2022 Nov 25;27(23):8219. doi: 10.3390/molecules27238219 (PMC9740992; doi:10.3390/molecules27238219)
Supplement: Supplementary file 1 [file molecules-27-08219-s001.zip › molecules-2054849-supplementary.pdf]

molecules

Supplementary Materials

Urea Synthesis from Isocyanides and *O*-Benzoyl Hydroxylamines Catalyzed by a Copper Salt

Ning Yu [1], Jing-Fang Lv [1], Shi-Mei He [1], Yanyan Cui [2,*], Ye Wei [1,*] and Kun Jiang [1,*]

[1] School of Chemistry and Chemical Engineering, Southwest University, Chongqing 400715, China
[2] Department of Cell Biology and Genetics, Chongqing Medical University, Chongqing 400016, China
* Correspondence: congjinzhang@163.com (Y.C.); weiye712@swu.edu.cn (Y.W.); kjiang@swu.edu.cn (K.J.)

Table of Contents

Materials and Methods S2

Preparation of Substrates S3

General Procedures Toward Ureas S4

^{1}H, ^{13}C and ^{19}F NMR Spectra S13

1. Meterials and Methods

General. All reactions dealing with air- and moisture-sensitive compounds were carried out in dry reaction vessels under a nitrogen atmosphere. ^{1}H and ^{13}C nuclear magnetic resonance (NMR) spectra were recorded on Bruker 600 MHz NMR spectrometer. ^{1}H and ^{13}C NMR spectra are reported in parts per million (ppm) downfield from an internal standard, tetramethylsilane (0 ppm) and $CHCl_3$ (77.0 ppm), respectively. HRMS (m/z) was recorded using ESI (Q-TOF) mode. Melting points were determined using a capillary melting point apparatus and are uncorrected.

Materials. Unless otherwise noted, materials were purchased from commercial suppliers and were used as received. Anhydrous tetrahydrofuran was distilled over Na and stored under N_2.

2. Preparation of Substrates

Preparation of Isocyanides

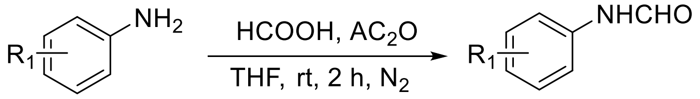

***N*-Formylation.** Acetyl formyl anhydride (prepared by stirring 2.5 equiv of acetic anhydride and 2.5 equiv of formic acid at 55 °C for 2h) was added dropwise at 0 °C to a stirred solution of the aniline (9 mmol) in THF (15 mL), and the mixture was stirred for 2 h at room temperature. Then the saturated solution of $NaHCO_3$ was added, and the aqueous phase was extracted with ethyl acetate. The organic layer was dried over $MgSO_4$ and concentrated by rotary evaporation. The residue was purified by column chromatography (petroleum ether/ethyl acetate = 3:1) to give N-formylated products.

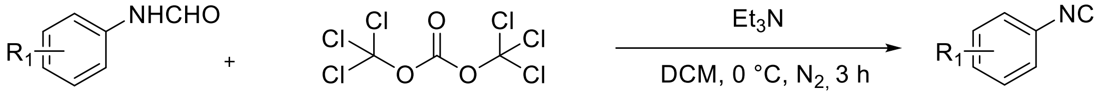

Dehydration. To a solution of the N-formylated products (5 mmol) and Et_3N (2.1 mL, 15 mmol) in CH_2Cl_2 (10 mL) at 0 °C was added triphosgene (0.74 g, 2.5 mmol) in CH_2Cl_2 (10 mL). The solution was stirred at 0 °C for 3 h. Then methanol was added to the suspension solution, and the solution was concentrated by rotary evaporation. The residue was purified by column chromatography (petroleum ether) to give the isocyanides.

Preparation of *O*-Benzoyl Hydroxylamines

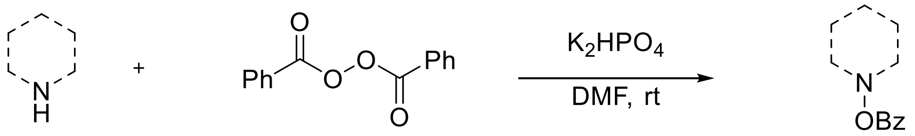

To a 100 mL flask charged with benzoyl peroxide (2 mmol), dipotassium hydrogen phosphate (3 mmol), and N,N′-dimethylformamide. Amine starting material (3 mmol) was added dropwise at room temperature. The suspension was stirred at ambient temperature for the indicated reaction time. The reaction was quenched with water (10 mL), and the contents were stirred vigorously for several minutes until all solids dissolved. The reaction mixture was extracted with ethyl acetate (3 × 30 mL). The organic phase was collected and washed with two 25 mL portions of saturated aq. $NaHCO_3$ solution, 25 mL of brine, dried over anhydrous Na_2SO_4, filtered, and concentrated under vacuum. The residue was purified by column chromatography on silica gel to give desired product *O*-benzoyl hydroxylamines.

3. General Procedures Toward Ureas

A 10 mL of Schlenk tube equipped with a stirrer bar was charged with isocyanides (0.2 mmol), *O*-benzoyl hydroxylamines (0.3 mmol), CuOAc (10 mol%), and *t*-BuONa (0.4 mmol). Then, the Schlenk tube was quickly evacuated and refilled with N_2 three times, followed by the addition of THF (2 mL). The Schlenk tube was sealed with a Teflon screwcap under an N_2 flow, and the reaction mixture was stirred at 30 °C for 12 h. Upon cooling to room temperature, the reaction mixture was diluted with 10 mL of ethyl acetate and filtered through a pad of silica gel, followed by washing the pad of the silica gel with ethyl acetate (20 mL). Subsequently, the filtrate was concentrated under reduced pressure. The residue was purified by flash chromatography on silica gel to afford the desired products.

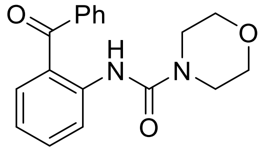

N-(2-benzoylphenyl)morpholine-4-carboxamide **(3a)**

According to the general procedure, a mixture consisting isocyanides (0.2 mmol，41.4mg), *O*-benzoyl hydroxylamines (0.3 mmol, 62.1mg), CuOAc (0.1 mmol, 2.5mg), *t*-BuONa (0.4 mmol, 38.4mg) and THF (2 mL) under a nitrogen atmosphere was stirred at 30 °C for 12 h to afford **3a** (53.3mg)**.** Yellow oil (86% yield, eluent = pentane/ethyl acetate = 3:1); ^{1}**H NMR** (600 MHz, $CDCl_3$): δ 10.91 (s, 1H), 8.56 – 8.54 (m, 1H), 7.67 – 7.64 (m, 2H), 7.60 – 7.54 (m, 3H), 7.50 – 7.47 (m, 2H), 7.00 – 6.96 (m, 1H), 3.76 (t, *J* = 6.0 Hz, 4H), 3.60 (t, *J* = 6.0 Hz, 4H); ^{13}**C NMR** (151 MHz, $CDCl_3$): δ 199.7, 153.8, 142.0, 138.1, 133.8, 133.2, 131.1, 128.6, 127.3, 120.9, 119.5, 119.4, 65.6, 43.0; **HRMS** (ESI): calcd for $C_{18}H_{19}N_2O_3$ $[M + H]^+$ 311.1390 found 311.1388.

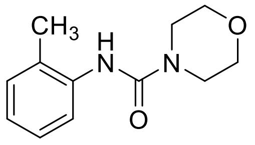

N-(o-tolyl)morpholine-4-carboxamide **(3b)**

According to the general procedure, a mixture consisting isocyanides (0.2 mmol，23.4mg), *O*-benzoyl hydroxylamines (0.3 mmol, 62.1mg), CuOAc (0.1 mmol, 2.5mg), *t*-BuONa (0.4 mmol, 38.4mg) and THF (2 mL) under a nitrogen atmosphere was stirred at 30 °C for 12 h to afford **3b** (21.1mg)**.** White solid (48% yield, eluent = pentane/ethyl acetate = 3:1); Mp = 152 − 153 °C; ^{1}**H NMR** (600 MHz, $CDCl_3$): δ 7.55 (t, *J* = 8.5 Hz, 1H), 7.17 (dd, *J* = 13.1, 7.3 Hz, 2H), 7.03 (t, *J* = 7.4 Hz, 1H), 6.18 (s, 1H), 3.72 – 3.70 (m, 4H), 3.47 – 3.41 (m, 4H), 2.23 (s, 3H); ^{13}**C NMR** (151 MHz, $CDCl_3$): δ 155.6, 136.8, 130.4, 129.5, 126.7, 124.5, 123.3, 66.5, 44.4, 17.7; **HRMS** (ESI): calcd for $C_{12}H_{17}N_2O_2$ $[M + H]^+$ 221.1285 found 221.1285.

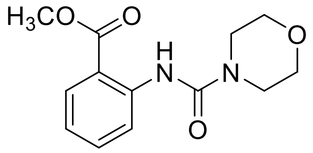

methyl 2-(morpholine-4-carboxamido)benzoate **(3c)**

According to the general procedure, a mixture consisting isocyanides (0.2 mmol，32.2mg), *O*-benzoyl hydroxylamines (0.3 mmol, 62.1mg), CuOAc (0.1 mmol, 2.5mg), *t*-BuONa (0.4 mmol, 38.4mg) and THF (2 mL) under a nitrogen atmosphere was stirred at 30 ℃ for 12 h to afford **3c** (31.7mg)**.** White solid (60% yield, eluent = pentane/ethyl acetate = 1:3); Mp = 121 − 123 °C; **^{1}H NMR** (600 MHz, $CDCl_3$)**:** δ 8.81 (s, 1H), 8.18 – 8.14 (m, 1H), 7.39 (t, *J* = 7.5 Hz, 1H), 7.18 (d, *J* = 7.4 Hz, 1H), 7.00 (t, *J* = 7.4 Hz, 1H), 3.74 – 3.70 (m, 7H), 3.48 – 3.47 (m, 4H); **^{13}C NMR** (151 MHz, $CDCl_3$)**:**δ 170.2, 154.7, 139.3, 131.3, 127.6, 122.2, 121.6, 121.5, 66.9, 66.5, 44.1; **HRMS** (ESI): calcd for $C_{13}H_{17}N_2O_4$ $[M + H]^+$ 265.1183 found 265.1183.

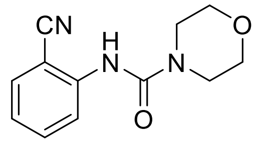

N-(2-cyanophenyl)morpholine-4-carboxamide **(3d)**

According to the general procedure, a mixture consisting isocyanides (0.2 mmol，25.6mg), *O*-benzoyl hydroxylamines (0.3 mmol, 62.1mg), CuOAc (0.1 mmol, 2.5mg), *t*-BuONa (0.4 mmol, 38.4mg) and THF (2 mL) under a nitrogen atmosphere was stirred at 30 ℃ for 12 h to afford **3d** (24.9mg)**.** Yellow solid (54% yield, eluent = pentane/ethyl acetate = 3:1); Mp = 162 − 163 °C; **^{1}H NMR** (600 MHz, $CDCl_3$): δ 8.25 (d, *J* = 8.5 Hz, 1H), 7.58 – 7.50 (m, 2H), 7.09 (t, *J* = 7.6 Hz, 1H), 6.98 (s, 1H), 3.77 – 3.76 (m, 4H), 3.54 – 3.53 (m, 4H); **^{13}C NMR** (151 MHz, $CDCl_3$): δ 153.7, 142.0, 134.2, 131.8, 122.8, 120.5, 116.9, 101.2, 66.4, 44.3; **HRMS** (ESI): calcd for $C_{12}H_{14}N_3O_2$ $[M + H]^+$ 232.1081 found 232.1082.

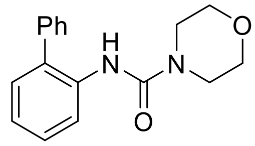

N-([1,1'-biphenyl]-2-yl)morpholine-4-carboxamide **(3e)**

According to the general procedure, a mixture consisting isocyanides (0.2 mmol，35.8mg), *O*-benzoyl hydroxylamines (0.3 mmol, 62.1mg), CuOAc (0.1 mmol, 2.5mg), PhONa (0.4 mmol, 46.4mg) and THF (2 mL) under a nitrogen atmosphere was stirred at 30 ℃ for 12 h to afford **3e** (41.2mg)**.** White solid (73% yield, eluent = pentane/ethyl acetate = 3:1); Mp = 118 − 119 °C; **^{1}H NMR** (600 MHz, $CDCl_3$): δ 8.10 (d, *J* = 8.2 Hz, 1H), 7.48 (t, *J* = 7.5 Hz, 2H), 7.42 –7.33 (m, 4H), 7.22 (dd, *J* = 7.5, 1.1 Hz, 1H), 7.11 (t, *J* = 7.5 Hz, 1H), 6.47 (s, 1H), 3.61 (t, *J* = 6.0 Hz, 4H), 3.22 (t, *J* = 6.0 Hz, 4H); **^{13}C NMR** (151 MHz, $CDCl_3$): δ 154.8, 138.6, 135.8, 131.9, 129.7, 129.3, 129.1, 128.5, 128.0, 123.1, 121.0, 66.4, 44.1; **HRMS** (ESI): calcd for $C_{17}H_{19}N_2O_2$ $[M + H]^+$ 283.1441 found 283.1439.

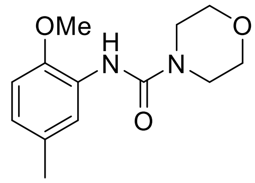

N-(2-methoxy-5-methylphenyl)morpholine-4-carboxamide **(3f)**

According to the general procedure, a mixture consisting isocyanides (0.2 mmol，29.4mg), *O*-benzoyl hydroxylamines (0.3 mmol, 62.1mg), CuOAc (0.1 mmol, 2.5mg), *t*-BuONa (0.4 mmol, 38.4mg) and THF (2 mL) under a nitrogen atmosphere was stirred at 30 °C for 12 h to afford **3f** (22.5mg). Yellow oil, (45% yield, eluent = pentane/ethyl acetate = 3:1); ^{1}**H NMR** (600 MHz, $CDCl_3$): δ 7.99 (s, 1H), 7.05 (s, 1H), 6.75 (m, 2H), 3.84 (s, 3H), 3.74 (t, *J* = 6.0 Hz, 4H), 3.49 (t, *J* = 6.0 Hz, 4H), 2.29 (s, 3H); ^{13}**C NMR** (151 MHz, $CDCl_3$): δ 154.8, 145.7, 130.7, 128.2, 122.4, 119.8, 109.7, 66.5, 55.9, 44.2, 21.0; **HRMS** (ESI): calcd for $C_{13}H_{19}N_2O_3$ $[M + H]^+$ 251.1390 found 251.1389.

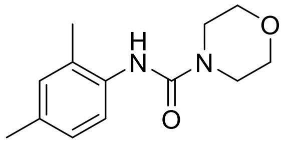

N-(2,4-dimethylphenyl)morpholine-4-carboxamide **(3g)**

According to the general procedure, a mixture consisting isocyanides (0.2 mmol，26.2mg), *O*-benzoyl hydroxylamines (0.3 mmol, 62.1mg), CuOAc (0.1 mmol, 2.5mg), *t*-BuONa (0.4 mmol, 38.4mg) and THF (2 mL) under a nitrogen atmosphere was stirred at 30 °C for 12 h to afford **3g** (18.7mg). Yellow solid (40% yield, eluent = pentane/ethyl acetate = 3:1); Mp = 137 − 138 °C; ^{1}**H NMR** (600 MHz, $CDCl_3$): δ 7.37 (d, *J* = 8.5 Hz, 1H), 7.00 – 6.95 (m, 2H), 6.09 (s, 1H), 3.71 (t, *J* = 6.0 Hz, 4H), 3.43 (t, *J* = 6.0 Hz, 4H), 2.28 (s, 3H), 2.19 (s, 3H); ^{13}**C NMR** (151 MHz, $CDCl_3$): δ 155.9, 134.3, 134.0, 131.1, 129.9, 127.3, 123.8, 66.5, 44.4, 20.8, 17.7; **HRMS** (ESI): calcd for $C_{13}H_{19}N_2O_2$ $[M + H]^+$ 235.1441 found 235.1448.

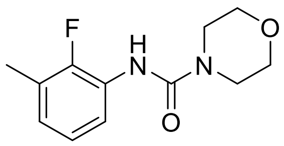

N-(2-fluoro-3-methylphenyl)morpholine-4-carboxamide **(3h)**

According to the general procedure, a mixture consisting isocyanides (0.2 mmol，27.0mg), *O*-benzoyl hydroxylamines (0.3 mmol, 62.1mg), CuOAc (0.1 mmol, 2.5mg), *t*-BuONa (0.4 mmol, 38.4mg) and THF (2 mL) under a nitrogen atmosphere was stirred at 50 °C for 12 h to afford **3h** (15.7mg). Yellow solid (33% yield, eluent = pentane/ethyl acetate = 3:1); Mp = 109 − 110 °C; ^{1}**H NMR** (600 MHz, $CDCl_3$): δ 7.88 (t, *J* = 7.8 Hz, 1H), 6.98 (t, *J* = 7.9 Hz, 1H), 6.83 (t, *J* = 7.4 Hz, 1H), 6.57 (s, 1H), 3.75 (t, *J* = 4.8 Hz, 4H), 3.50 (t, *J* = 4.8 Hz, 4H), 2.26 (d, *J* = 1.8 Hz, 3H); ^{13}**C NMR** (151 MHz, $CDCl_3$): δ 154.5, 151.3(d, $^{1}J_{C-F}$ = 238.6Hz), 127.0(d, $^{2}J_{C-F}$ = 10.6Hz), 124.8(d, $^{3}J_{C-F}$ = 6.0Hz), 124.2(d, $^{2}J_{C-F}$ = 16.6Hz), 123.8(d, $^{3}J_{C-F}$ = 4.5Hz), 118.9, 66.5, 44.3, 14.4(d, $^{3}J_{C-F}$ = 4.5Hz); ^{19}**F NMR** (565 MHz, $CDCl_3$): δ -137.2; **HRMS** (ESI): calcd for $C_{12}H_{16}FN_2O_2$ $[M + H]^+$ 239.1190 found 239.1191.

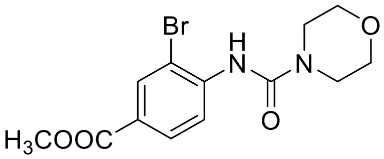

methyl 3-bromo-4-(piperidine-1-carboxamido)benzoate **(3i)**

According to the general procedure, a mixture consisting isocyanides (0.2 mmol，47.8mg), *O*-benzoyl hydroxylamines (0.3 mmol, 62.1mg), CuOAc (0.1 mmol, 2.5mg), PhONa (0.4 mmol, 46.4mg) and THF (2 mL) under a nitrogen atmosphere was stirred at 30 °C for 12 h to afford **3i** (31.5mg)**.** White solid (46% yield, eluent = pentane/ethyl acetate = 3:1); Mp = 121 − 122 °C; **^{1}H NMR** (600 MHz, $CDCl_3$): δ 8.33 (dd, *J* = 11.8, 6.9 Hz, 1H), 8.21 – 8.15 (m, 1H), 7.95 (dd, *J* = 12.2 , 4.8 Hz, 1H), 7.25 (s, 1H), 3.88 (s, 3H), 3.79 – 3.72 (m, 4H), 3.55 – 3.49 (m, 4H); **^{13}C NMR** (151 MHz, $CDCl_3$): δ 165.5, 153.6, 140.7, 133.5, 130.0, 125.2, 119.4, 112.1, 66.4, 52.1, 44.3; **HRMS** (ESI): calcd for $C_{13}H_{16}BrN_2O_4$ $[M + H]^+$ 343.0288 found 343.0287.

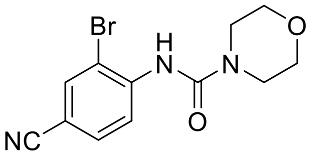

N-(2-bromo-4-cyanophenyl)morpholine-4-carboxamide **(3j)**

According to the general procedure, a mixture consisting isocyanides (0.2 mmol，41.2mg), *O*-benzoyl hydroxylamines (0.3 mmol, 62.1mg), CuOAc (0.1 mmol, 2.5mg), *t*-BuONa (0.4 mmol, 38.4mg) and THF (2 mL) under a nitrogen atmosphere was stirred at 30 °C for 12 h to afford **3j** (21.0mg)**.** White solid (34% yield, eluent = pentane/ethyl acetate = 3:1); Mp = 121 − 122 °C; **^{1}H NMR** (600 MHz, $CDCl_3$): δ 8.42 (d, *J* = 8.7 Hz, 1H), 7.79 (d, *J* = 1.8 Hz, 1H), 7.57 (dd, *J* = 8.7, 1.7 Hz, 1H), 7.27 (s, 1H), 3.78 (t, *J* = 6.0 Hz, 4H), 3.54 (t, *J* = 6.0 Hz, 4H); **^{13}C NMR** (151 MHz, $CDCl_3$): δ 153.2, 141.0, 135.4, 132.5, 120.1, 117.6, 112.1, 106.6, 66.3, 44.3; **HRMS** (ESI): $[M + K]^+$ calcd for $C_{12}H_{12}BrKN_3O_2$ 347.9744 found 347.9741.

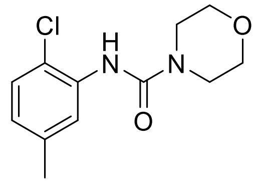

N-(2-chloro-5-methylphenyl)morpholine-4-carboxamide **(3k)**

According to the general procedure, a mixture consisting isocyanides (0.2 mmol，30.2mg), *O*-benzoyl hydroxylamines (0.3 mmol, 62.1mg), CuOAc (0.1 mmol, 2.5mg), *t*-BuONa (0.4 mmol, 38.4mg) and THF (2 mL) under a nitrogen atmosphere was stirred at 30 °C for 12 h to afford **3k** (25.9mg)**.** Yellow solid (51% yield, eluent = pentane/ethyl acetate = 3:1); Mp = 106 − 107 °C; **^{1}H NMR** (600 MHz, $CDCl_3$): δ 8.02 (d, *J* = 1.2 Hz, 1H), 7.20 (d, *J* = 8.2 Hz, 1H), 6.93 (s, 1H), 6.78 (dd, *J* = 8.1, 1.4 Hz, 1H), 3.76 (t, *J* = 6.0 Hz, 4H), 3.51 (t, *J* = 6.0 Hz, 4H), 2.32 (s, 3H); **^{13}C NMR** (151 MHz, $CDCl_3$): δ 154.3, 137.9, 135.1, 128.3, 124.2, 121.4, 119.4, 66.4, 44.2, 21.3; **HRMS** (ESI): calcd for $C_{12}H_{16}ClN_2O_2$ $[M + H]^+$ 255.0895 found 255.0893.

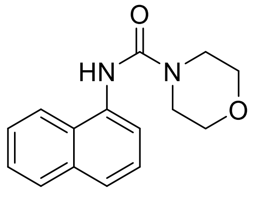

N-(naphthalen-1-yl)morpholine-4-carboxamide **(3l)**

According to the general procedure, a mixture consisting isocyanides (0.2 mmol，30.6mg), *O*-benzoyl hydroxylamines (0.3 mmol, 62.1mg), CuOAc (0.1 mmol, 2.5mg), *t*-BuONa (0.4 mmol, 38.4mg) and THF (2 mL) under a nitrogen atmosphere was stirred at 30 °C for 12 h to afford **3l** (31.2mg). Yellow solid (61% yield, eluent = pentane/ethyl acetate = 3:1); Mp = 192 − 193 °C; **^{1}H NMR** (600 MHz, $CDCl_3$): δ 7.91 – 7.80 (m, 2H), 7.68 (d, *J* = 8.2 Hz, 1H), 7.60 (d, *J* = 7.3 Hz, 1H), 7.53 – 7.47 (m, 2H), 7.44 (t, *J* = 7.8 Hz, 4H), 6.69 (s, 1H), 3.68 (t, *J* = 6.0 Hz, 4H), 3.45 (t, *J* = 6.0 Hz, 4H); **^{13}C NMR** (151 MHz, $CDCl_3$): δ 156.2, 134.3, 133.8, 128.7, 128.3, 126.1, 125.9, 125.7, 125.4, 121.3, 121.2, 66.5, 44.5; **HRMS** (ESI): calcd for $C_{15}H_{17}N_2O_3$ $[M + H]^+$ 257.1285 found 257.1283.

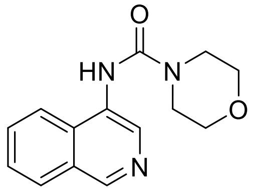

N-(isoquinolin-4-yl)morpholine-4-carboxamide **(3m)**

According to the general procedure, a mixture consisting isocyanides (0.2 mmol，30.8mg), *O*-benzoyl hydroxylamines (0.3 mmol, 62.1mg), CuOAc (0.1 mmol, 2.5mg), *t*-BuONa (0.4 mmol, 38.4mg) and THF (2 mL) under a nitrogen atmosphere was stirred at 30 °C for 12 h to afford **3m** (40.6mg). Yellow solid (79% yield, eluent = pentane/ethyl acetate = 1:6); Mp = 176 − 177 °C; **^{1}H NMR** (600 MHz, $CDCl_3$): δ 8.99 (s, 1H), 8.43 (s, 1H), 7.91 (d, *J* = 8.1 Hz, 1H), 7.74 (d, *J* = 8.4 Hz, 1H), 7.64 (t, *J* = 7.6 Hz, 1H), 7.56 (t, *J* = 7.5 Hz, 1H), 7.12 (s, 1H), 3.63 (t, *J* = 6.0 Hz, 4H), 3.42 (t, *J* = 6.0 Hz, 4H); **^{13}C NMR** (151 MHz, $CDCl_3$): δ 156.1, 149.8, 139.0, 131.8, 130.3, 129.4, 128.8, 127.8, 127.3, 121.5, 66.5, 44.4; **HRMS** (ESI): calcd for $C_{14}H_{16}N_3O_2$ $[M + H]^+$ 258.1237 found 258.1237.

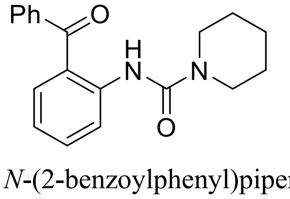

N-(2-benzoylphenyl)piperidine-1-carboxamide **(3n)**

According to the general procedure, a mixture consisting isocyanides (0.2 mmol，41.4mg), *O*-benzoyl hydroxylamines (0.3 mmol, 61.5), CuOAc (0.1 mmol, 2.5mg), *t*-BuONa (0.4 mmol, 38.4mg) and THF (2 mL) under a nitrogen atmosphere was stirred at 30 °C for 12 h to afford **3n** (40.7mg). Yellow oil (66% yield, eluent = pentane/ethyl acetate = 3:1); **^{1}H NMR** (600 MHz, $CDCl_3$): δ 10.82 (s, 1H), 8.53 (d, *J* = 8.9 Hz, 1H), 7.66 (d, *J* = 7.6 Hz, 2H), 7.58 – 7.52 (m, 3H), 7.47 (t, *J* = 7.6 Hz, 2H), 6.93 (t, *J* = 7.6 Hz, 1H), 3.59 – 3.53 (m, 4H), 1.70 – 1.60 (m, 6H); **^{13}C NMR** (151 MHz, $CDCl_3$): δ 199.5, 153.6, 142.5, 138.4, 133.6, 133.1, 130.9, 128.6, 127.2, 120.8,

119.6, 118.9, 44.1, 24.8, 23.5; **HRMS** (ESI): calcd for $C_{19}H_{21}N_2O_2$ $[M + H]^+$ 309.1598 found 309.1595.

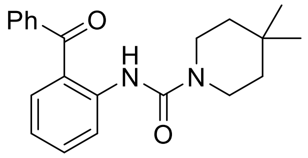

N-(2-benzoylphenyl)-4,4-dimethylpiperidine-1-carboxamide **(3o)**

According to the general procedure, a mixture consisting isocyanides (0.2 mmol, 41.4mg), *O*-benzoyl hydroxylamines (0.3 mmol, 70.0mg), CuOAc (0.1 mmol, 2.5mg), *t*-BuONa (0.4 mmol, 38.4mg) and THF (2 mL) under a nitrogen atmosphere was stirred at 30 °C for 12 h to afford **3o** (42.3mg)**.** Yellow oil (63% yield, eluent = pentane/ethyl acetate = 3:1); **^{1}H NMR** (600 MHz, $CDCl_3$): δ 10.84 (s, 1H), 8.53 (d, *J* = 9.0 Hz, 1H), 7.66 (d, *J* = 7.4 Hz, 2H), 7.58 (t, *J* = 7.4 Hz, 1H), 7.54 (dd, *J* = 7.4, 5.8 Hz, 2H), 7.48 (t, *J* = 7.6 Hz, 2H), 6.94 (t, *J* = 7.6 Hz, 1H), 3.57 (t, *J* = 5.7 Hz, 4H), 1.45 (t, *J* = 5.8Hz, 4H), 0.99 (s, 6H); **^{13}C NMR** (151 MHz, $CDCl_3$): δ 200.5, 154.7, 143.5, 139.3, 134.6, 134.1, 132.0, 129.6, 128.2, 121.8, 120.6, 120.0, 40.7, 38.4, 28.9, 27.7.

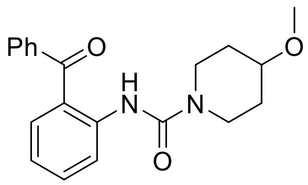

N-(2-benzoylphenyl)-4-methoxypiperidine-1-carboxamide **(3p)**

According to the general procedure, a mixture consisting isocyanides (0.2 mmol, 41.4mg), *O*-benzoyl hydroxylamines (0.3 mmol, 70.5mg), CuOAc (0.1 mmol, 2.5mg), *t*-BuONa (0.4 mmol, 38.4mg) and THF (2 mL) under a nitrogen atmosphere was stirred at 30 °C for 12 h to afford **3p** (43.3mg)**.** Yellow oil (64% yield, eluent = pentane/ethyl acetate = 3:1); **^{1}H NMR** (600 MHz, $CDCl_3$): δ 10.89 (s, 1H), 8.52 (d, *J* = 8.9 Hz, 1H), 7.66 (d, *J* = 7.3 Hz, 2H), 7.58 (t, *J* = 7.4 Hz, 1H), 7.55 – 7.52 (m, 2H), 7.48 (t, *J* = 7.7 Hz, 2H), 6.95 (t, *J* = 7.6 Hz, 1H), 3.90 – 3.85 (m, 2H), 3.47 – 3.43 (m, 1H), 3.38 – 3.34 (m, 5H), 1.94 (ddd, *J* = 12.6, 6.9, 3.4 Hz, 2H), 1.69 – 1.67 (m, 1H), 1.66 – 1.63 (m, 1H); **^{13}C NMR** (151 MHz, $CDCl_3$): δ 200.6, 154.5, 143.4, 139.3, 134.7, 134.1, 132.0, 129.6, 128.3, 121.8, 120.6, 120.1, 75.5, 55.7, 41.3, 30.5; **HRMS** (ESI): calcd for $C_{20}H_{22}N_2NaO_3$ $[M + Na]^+$ 361.1523 found 361.1529.

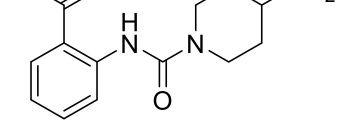

ethyl 1-((2-benzoylphenyl)carbamoyl)piperidine-4-carboxylate **(3q)**

According to the general procedure, a mixture consisting isocyanides (0.2 mmol, 41.4mg), *O*-benzoyl hydroxylamines (0.3 mmol, 78.9mg), CuOAc (0.1 mmol, 2.5mg), *t*-BuONa (0.4 mmol, 38.4mg) and THF (2 mL) under a nitrogen atmosphere was stirred at 30 °C for 12 h to afford **3q** (51.7mg)**.** Yellow oil (68% yield, eluent = pentane/ethyl acetate = 3:1); **^{1}H NMR** (600 MHz, $CDCl_3$): δ 10.89 (s, 1H), 8.52 (dd, *J* = 8.9, 0.8 Hz, 1H), 7.69 – 7.62 (m, 2H), 7.60 – 7.51 (m, 3H),

7.48 (t, J = 7.7 Hz, 2H), 6.99 – 6.92 (m, 1H), 4.20 – 4.11 (m, 4H), 3.13 – 3.05 (m, 2H), 2.53 (tt, J = 10.7, 4.0 Hz, 1H), 2.02 – 1.99 (m, 2H), 1.81 – 1.75 (m, 2H), 1.25 (t, J = 7.1 Hz, 3H); **^{13}C NMR** (151 MHz, $CDCl_3$): δ 199.6, 173.2, 153.5, 142.3, 138.2, 133.7, 133.1, 131.0, 128.6, 127.3, 120.8, 119.6, 119.2, 59.6, 42.4, 40.0, 27.0, 13.2; **HRMS** (ESI): calcd for $C_{22}H_{25}N_2O_4$ [M + H]$^+$ 381.1809 found 381.1806.

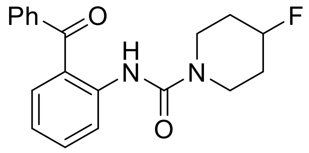

N-(2-benzoylphenyl)-4-fluoropiperidine-1-carboxamide **(3r)**

According to the general procedure, a mixture consisting isocyanides (0.2 mmol，41.4mg), *O*-benzoyl hydroxylamines (0.3 mmol, 61.6mg), CuOAc (0.1 mmol, 2.5mg), *t*-BuONa (0.4 mmol, 38.4mg) and THF (2 mL) under a nitrogen atmosphere was stirred at 30 °C for 12 h to afford **3r** (40.4mg)**.** Yellow oil (62% yield, eluent = pentane/ethyl acetate = 3:1); **^{1}H NMR** (600 MHz, $CDCl_3$): δ 10.94 (s, 1H), 8.52 (d, J = 8.9 Hz, 1H), 7.66 (d, J = 7.2 Hz, 2H), 7.60 – 7.54 (m, 3H), 7.48 (t, J = 7.7 Hz, 2H), 6.97 (t, J = 7.6 Hz, 1H), 4.95 – 4.82 (m, 1H), 3.73 – 3.64 (m, 4H), 1.98 – 1.91 (m, 4H); **^{13}C NMR** (151 MHz, $CDCl_3$): δ 200.6, 154.4, 143.2, 139.2, 134.7, 134.2, 132.1, 129.6, 128.3, 121.8, 120.6, 120.3 , 87.8 (d, $^1J_{C\text{-}F}$ = 172.1Hz), 40.1 (d, $^3J_{C\text{-}F}$ = 6.0Hz), 31.1 (d, $^2J_{C\text{-}F}$ = 21.1Hz).

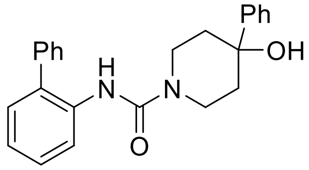

N-([1,1'-biphenyl]-2-yl)-4-hydroxy-4-phenylpiperidine-1-carboxamide **(3s)**

According to the general procedure, a mixture consisting isocyanides (0.2 mmol，35.8mg), *O*-benzoyl hydroxylamines (0.3 mmol, 89.1mg), CuOAc (0.1 mmol, 2.5mg), PhONa (0.4 mmol, 46.4mg) and THF (2 mL) under a nitrogen atmosphere was stirred at 30 °C for 12 h to afford **3s** (44.6mg)**.** White solid (60% yield, eluent = pentane/ethyl acetate = 3:1); Mp = 108 − 109 °C; **^{1}H NMR** (600 MHz, $CDCl_3$): δ 8.11 (d, J = 8.1 Hz, 1H), 7.48 – 7.42 (m, 4H), 7.39 – 7.35 (m, 6H), 7.28 (t, J = 7.3 Hz, 1H), 7.22 (d, J = 7.5 Hz, 1H), 7.10 (t, J = 7.5 Hz, 1H), 6.58 (s, 1H), 3.68 (d, J = 13.0 Hz, 2H), 3.27 (td, J = 13.1, 2.1 Hz, 2H), 1.94 (td, J = 13.4, 4.6 Hz, 2H), 1.79 – 1.75 (m, 1H), 1.69 (d, J = 12.7 Hz, 2H); **^{13}C NMR** (151 MHz, $CDCl_3$): δ 154.7, 147.6, 138.7, 136.2, 131.7, 129.6, 129.3, 129.1, 128.5(2), 127.9, 127.4, 124.4, 122.8, 120.9, 71.4, 40.5, 38.0; **HRMS** (ESI): calcd for $C_{24}H_{25}N_2O_2$ [M + H]$^+$ 373.1911 found 373.1910.

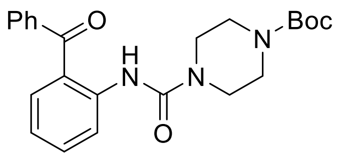

tert-butyl 4-((2-benzoylphenyl)carbamoyl)piperazine-1-carboxylate **(3t)**

According to the general procedure, a mixture consisting isocyanides (0.2 mmol, 41.4mg), *O*-benzoyl hydroxylamines (0.3 mmol, 91.8mg), CuOAc (0.1 mmol, 2.5mg), *t*-BuONa (0.4 mmol, 38.4mg) and THF (2 mL) under a nitrogen atmosphere was stirred at 30 °C for 12 h to afford **3t** (58.1mg). Yellow solid (71% yield, eluent = pentane/ethyl acetate = 3:1); Mp = 106 − 107 °C ; **^{1}H NMR** (600 MHz, $CDCl_3$): δ 10.92 (s, 1H), 8.56 – 8.51 (m, 1H), 7.67 – 7.65 (m, 2H), 7.58 – 7.54 (m, 3H), 7.48 (t, *J* = 7.7 Hz, 2H), 7.00 – 6.95 (m, 1H), 3.62 – 3.58 (m, 4H), 3.54 – 3.50 (m, 4H), 1.47 (s, 9H); **^{13}C NMR** (151 MHz, $CDCl_3$): δ 199.7, 153.6(2), 142.0, 138.1, 133.7, 133.2, 131.1, 128.6, 127.3, 120.9, 119.6, 119.4, 79.2, 42.6, 27.4; **HRMS** (ESI): calcd for $C_{23}H_{28}N_3O_4$ $[M + H]^+$ 410.2074 found 410.2074.

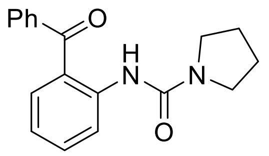

N-(2-benzoylphenyl)pyrrolidine-1-carboxamide **(3u)**

According to the general procedure, a mixture consisting isocyanides (0.2 mmol, 41.4mg), *O*-benzoyl hydroxylamines (0.3 mmol, 57.3mg), CuOAc (0.1 mmol, 2.5mg), *t*-BuONa (0.4 mmol, 38.4mg) and THF (2 mL) under a nitrogen atmosphere was stirred at 30 °C for 12 h to afford **3u** (18.2mg). White solid (31% yield, eluent = pentane/ethyl acetate = 3:1); Mp = 111 − 112 °C ; **^{1}H NMR** (600 MHz, $CDCl_3$): δ 10.61 (s, 1H), 8.63 (d, *J* = 8.8 Hz, 1H), 7.66 (d, *J* = 7.5 Hz, 2H), 7.60 – 7.50 (m, 3H), 7.48 (t, *J* = 7.6 Hz, 2H), 6.95 (t, *J* = 7.5 Hz, 1H), 3.57 (t, *J* = 6.6 Hz, 4H), 2.03 – 1.92 (m, 4H). **^{13}C NMR** (151 MHz, $CDCl_3$): δ 200.4, 153.9, 143.3, 139.4, 134.6, 134.0, 131.9, 129.6, 128.2, 121.6, 120.4, 119.9, 45.8, 25.5. **HRMS** (ESI): calcd for $C_{18}H_{19}N_2O_2$ $[M + H]^+$ 295.1441 found 295.1439.

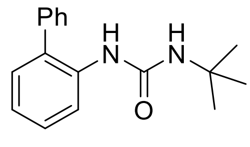

1-([1,1'-biphenyl]-2-yl)-3-(tert-butyl)urea **(3v)**

According to the general procedure, a mixture consisting isocyanides (0.2 mmol, 35.8mg), *O*-benzoyl hydroxylamines (0.3 mmol,57.9mg), CuOAc (0.1 mmol, 2.5mg), *t*-BuONa (0.4 mmol, 38.4mg) and THF (2 mL) under a nitrogen atmosphere was stirred at 30 °C for 12 h to afford **3v** (19.3mg). White solid (36% yield, eluent = pentane/ethyl acetate = 3:1); Mp = 151 − 152 °C; **^{1}H NMR** (600 MHz, $CDCl_3$) δ 7.79 (d, *J* = 7.9 Hz, 1H), 7.45 (t, *J* = 7.5 Hz, 2H), 7.37 (dd, *J* = 11.7, 4.3 Hz, 3H), 7.34 – 7.30 (m, 1H), 7.26 – 7.23 (m, 1H), 7.15 – 7.12 (m, 1H), 5.95 (s, 1H), 4.39 (s, 1H), 1.26 (s, 9H); **^{13}C NMR** (151 MHz,$CDCl_3$): δ 154.6, 138.8, 135.8, 133.5, 130.5, 129.2, 128.9, 128.5, 127.7, 123.9, 122.8, 50.7, 29.2; **HRMS** (ESI): calcd for $C_{17}H_{21}N_2O$ $[M + H]^+$ 269.1648 found 269.1647.

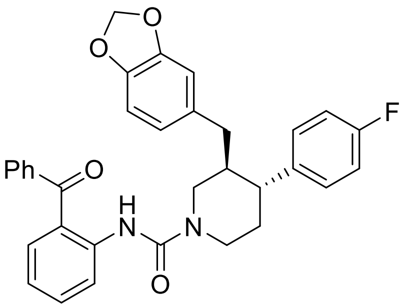

(3S,4R)-3-(benzo[d][1,3]dioxol-5-ylmethyl)-*N*-(2-benzoylphenyl)-4-(4-fluorophenyl)piperidine-1-carboxamide **(3w)**

According to the general procedure, a mixture consisting isocyanides (0.2 mmol，41.4mg), *O*-benzoyl hydroxylamines (0.3 mmol, 129.9mg), CuOAc (0.1 mmol, 2.5mg), *t*-BuONa (0.4 mmol, 38.4mg) and THF (2 mL) under a nitrogen atmosphere was stirred at 30 °C for 12 h to afford **3w** (81.5mg)**.** Yellow oil (76% yield, eluent = pentane/ethyl acetate = 3:1); **^{1}H NMR** (600 MHz, $CDCl_3$): δ 11.04 (s, 1H), 8.61 – 8.58 (m, 1H), 7.70 – 7.67 (m, 2H), 7.60 – 7.55 (m, 3H), 7.49 (t, *J* = 7.7 Hz, 2H), 7.15 (dd, *J* = 8.6 Hz, 2H), 6.99 – 6.96 (m, 3H), 6.61 (d, *J* = 8.5 Hz, 1H), 6.44 (d, *J* = 2.4 Hz, 1H), 6.18 (dd, *J* = 8.5, 2.5 H, 1H), 5.88 (s, 2H), 4.63 (d, *J* = 12.2 Hz, 1H), 4.43 (d, *J* = 13.2 Hz, 1H), 3.70 (dd, *J* = 9.4, 2.7 Hz, 1H), 3.51 (dd, *J* = 9.3, 6.6 Hz, 1H), 3.09 – 3.01 (m, 2H), 2.79 (td, J = 11.9, 3.8 Hz, 1H), 2.18 – 2.10 (m, 1H), 1.95 – 1.93 (m, 1H), 1.87 – 1.80 (m, 1H); **^{13}C NMR** (151 MHz, $CDCl_3$): δ 199.5, 160.7(d, $^1J_{C\text{-}F}$ = 244.6 Hz), 153.4(d, $^2J_{C\text{-}F}$ = 18.1 Hz), 147.2, 142.3, 140.7, 138.2, 137.8(2), 133.6, 133.1, 131.0, 128.7, 127.8(d, $^3J_{C\text{-}F}$ = 7.6 Hz), 127.2, 120.8, 119.5, 119.2, 114.5(d, $^2J_{C\text{-}F}$ = 21.1 Hz), 106.8, 104.6, 100.1, 97.1, 67.6, 46.7, 43.8, 43.2, 41.2, 32.9.

^{1}H, ^{13}C and ^{19}F NMR Spectra

^{1}H NMR of 3a

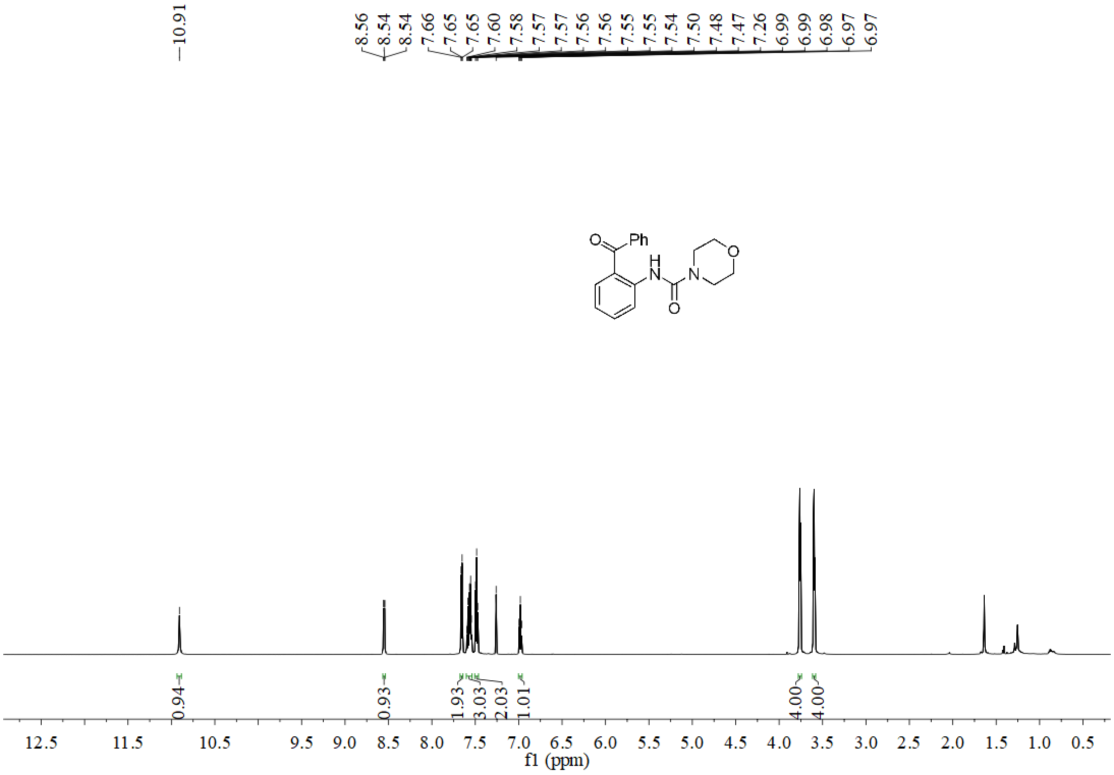

^{13}C NMR of 3a

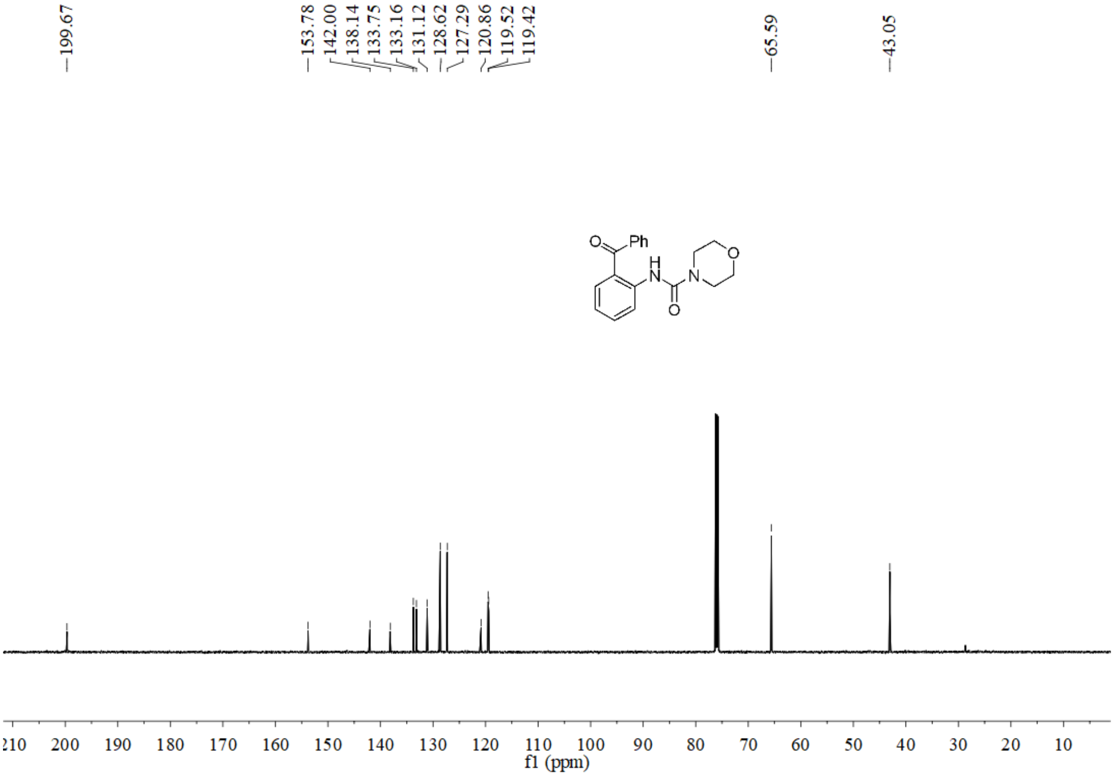

^{1}H NMR of 3b

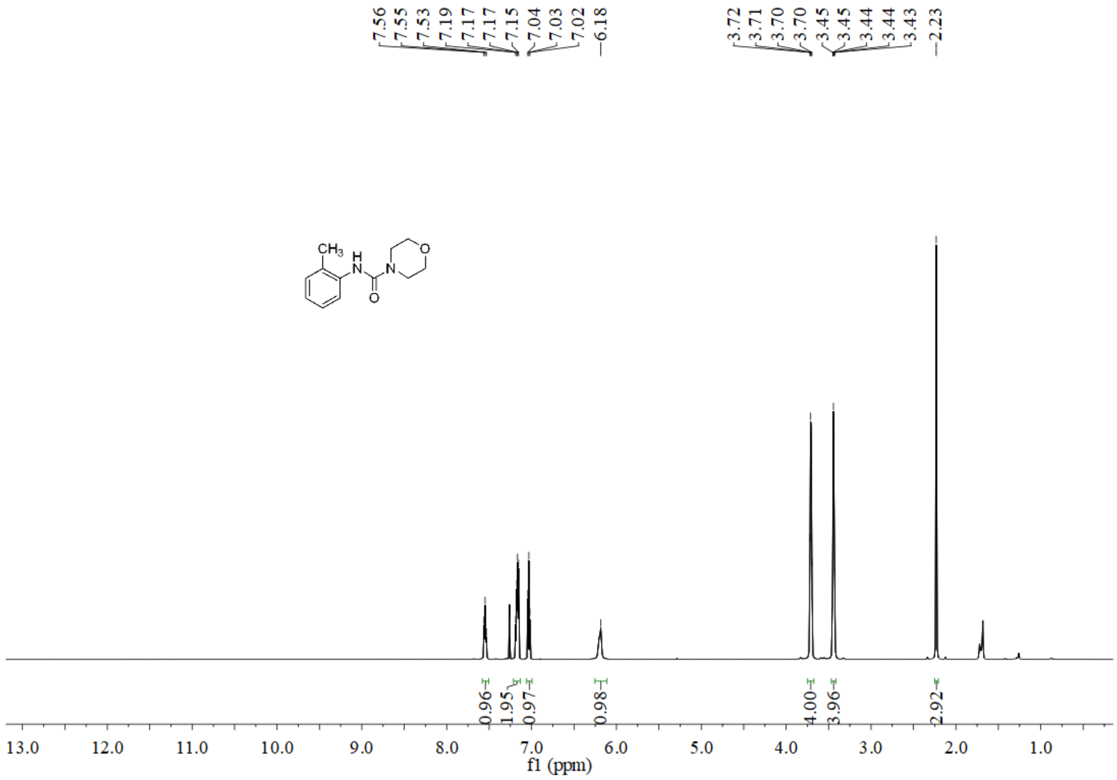

^{13}C NMR of 3b

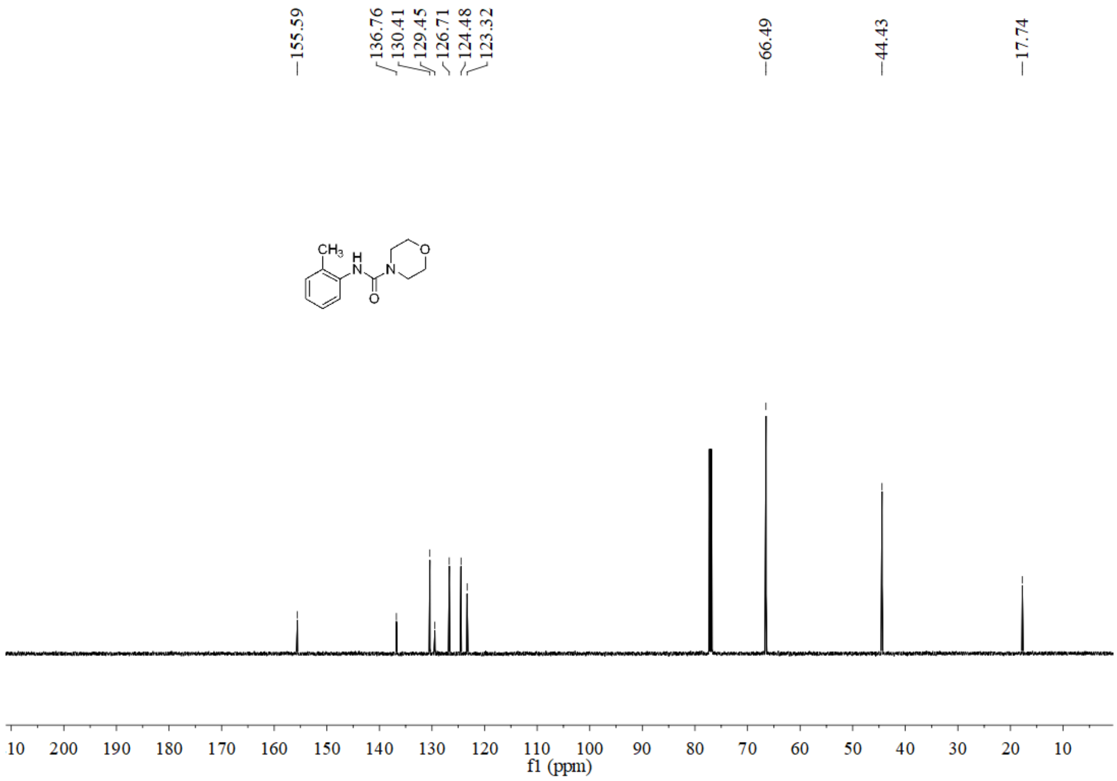

^{1}H NMR of 3c

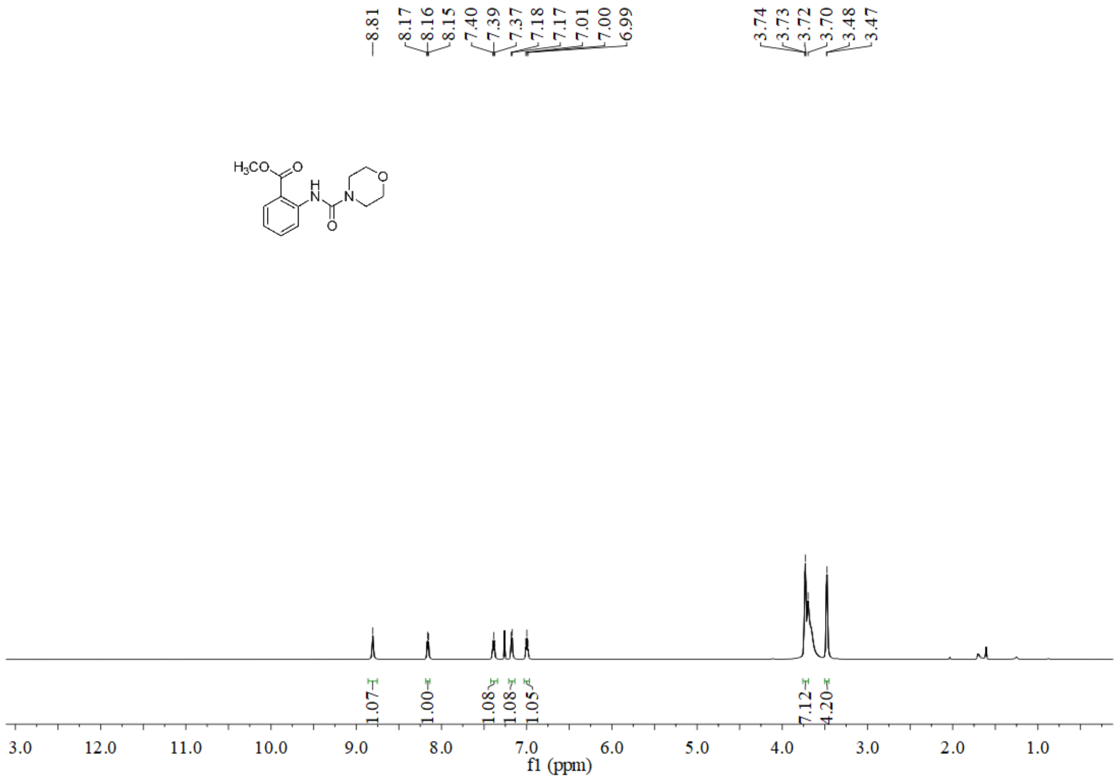

^{13}C NMR of 3c

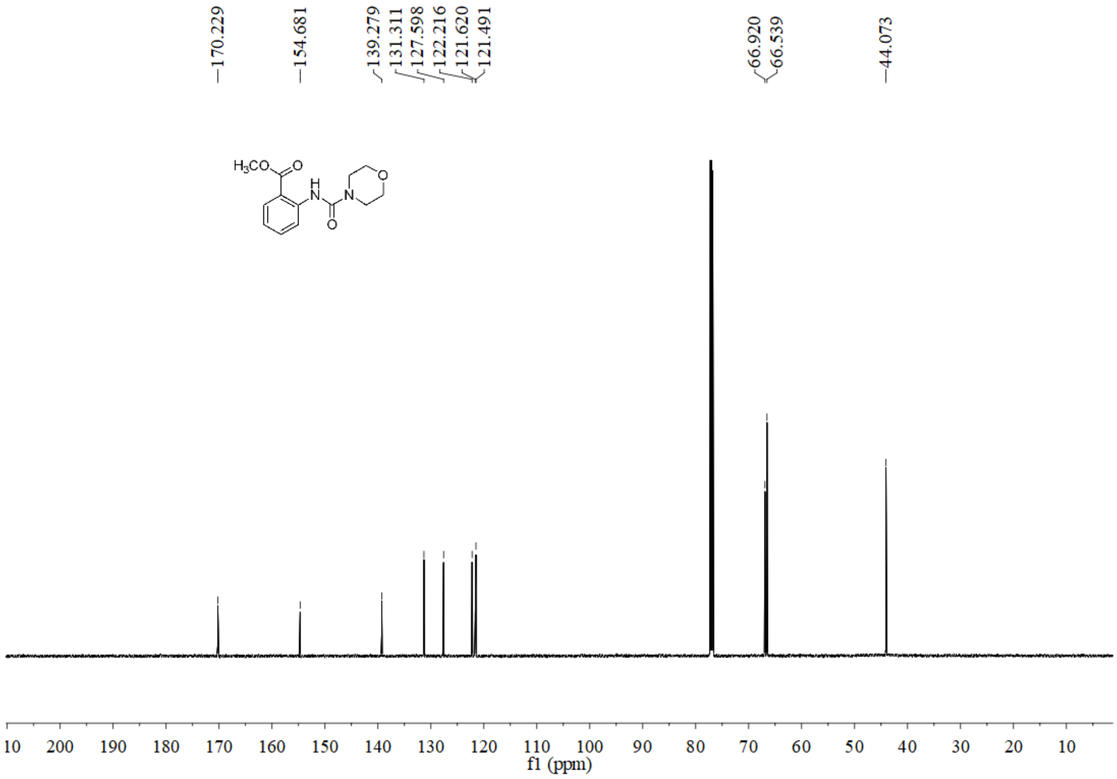

^{1}H NMR of 3d

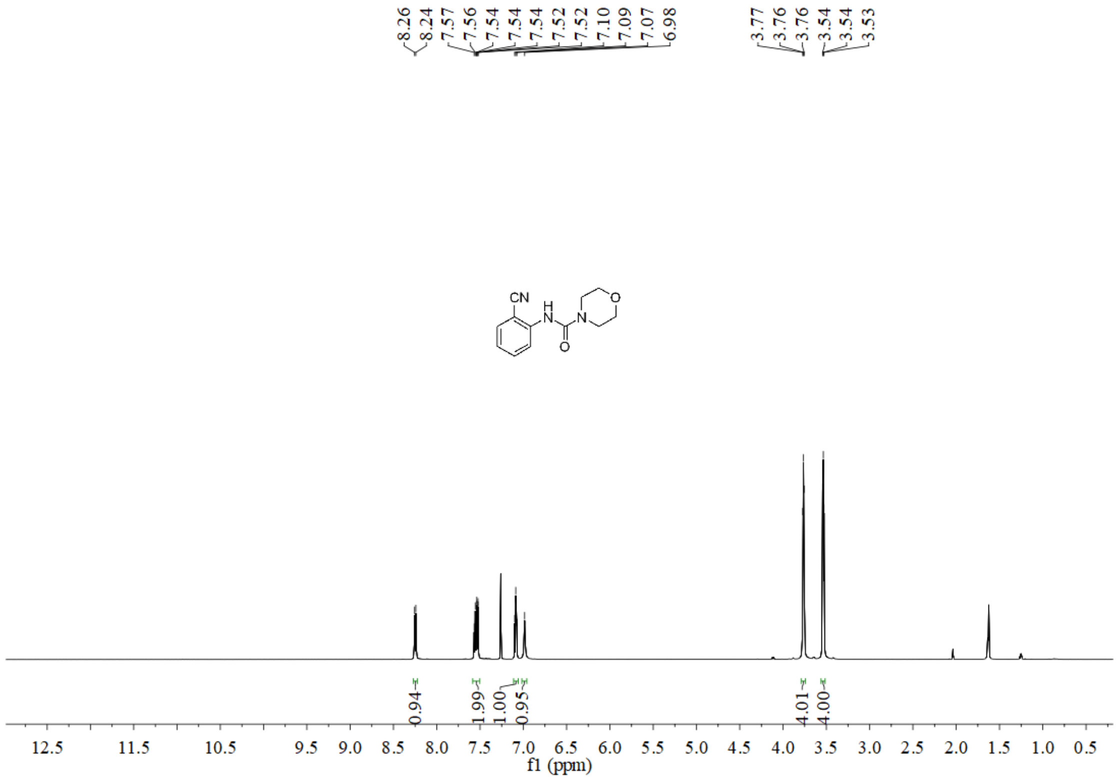

^{13}C NMR of 3d

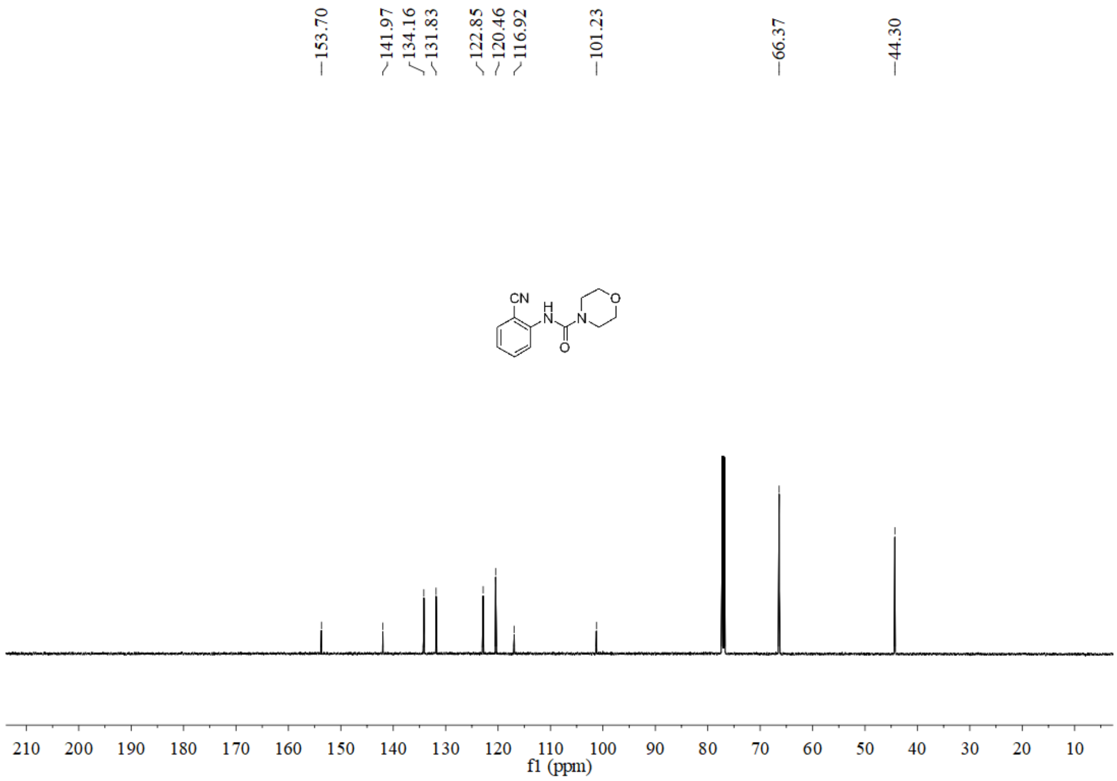

^{1}H NMR of 3e

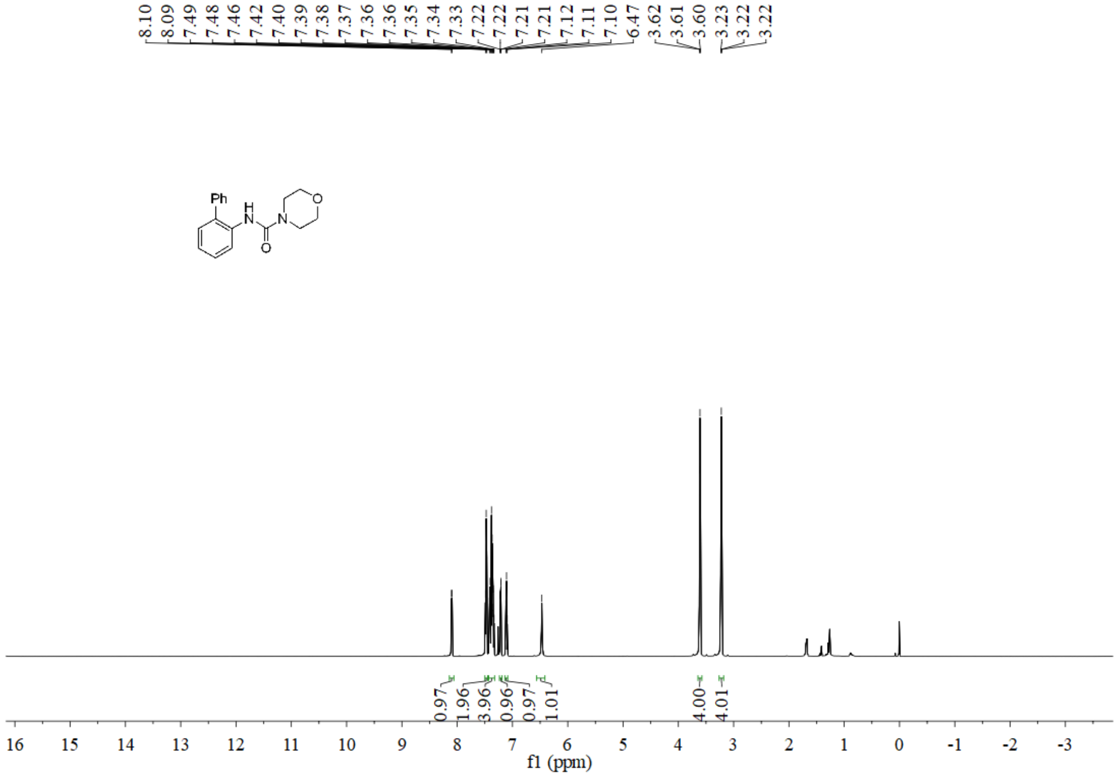

^{13}C NMR of 3e

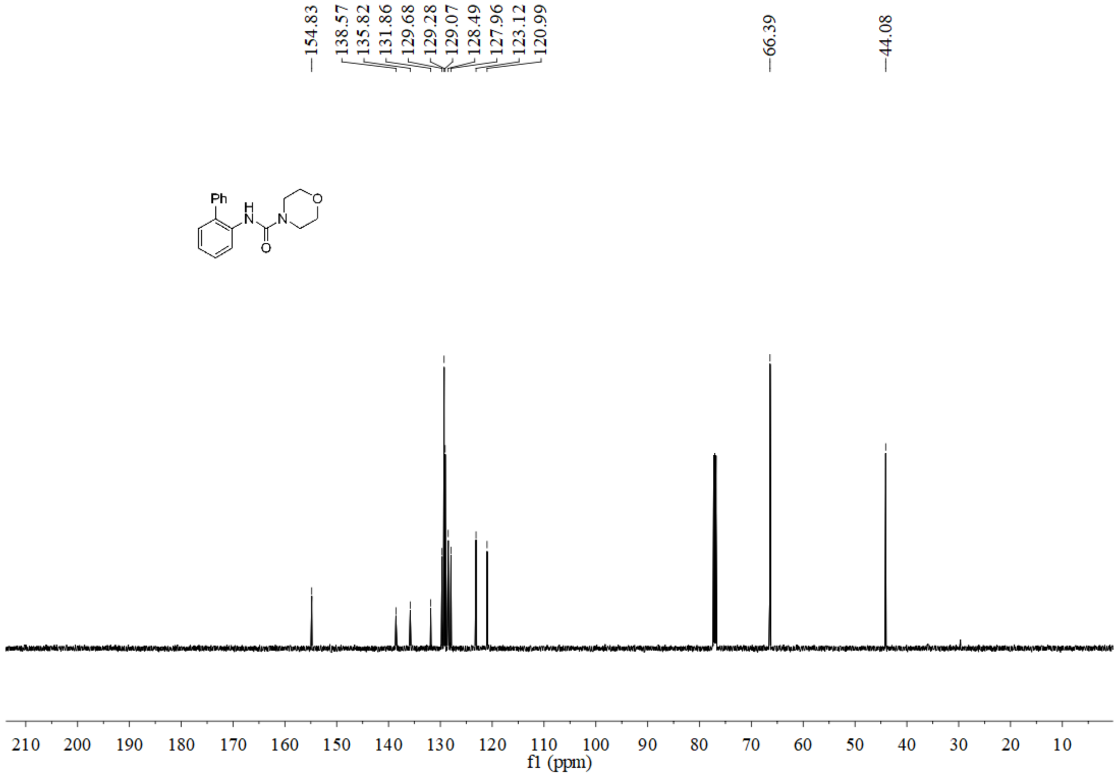

^{1}H NMR of 3f

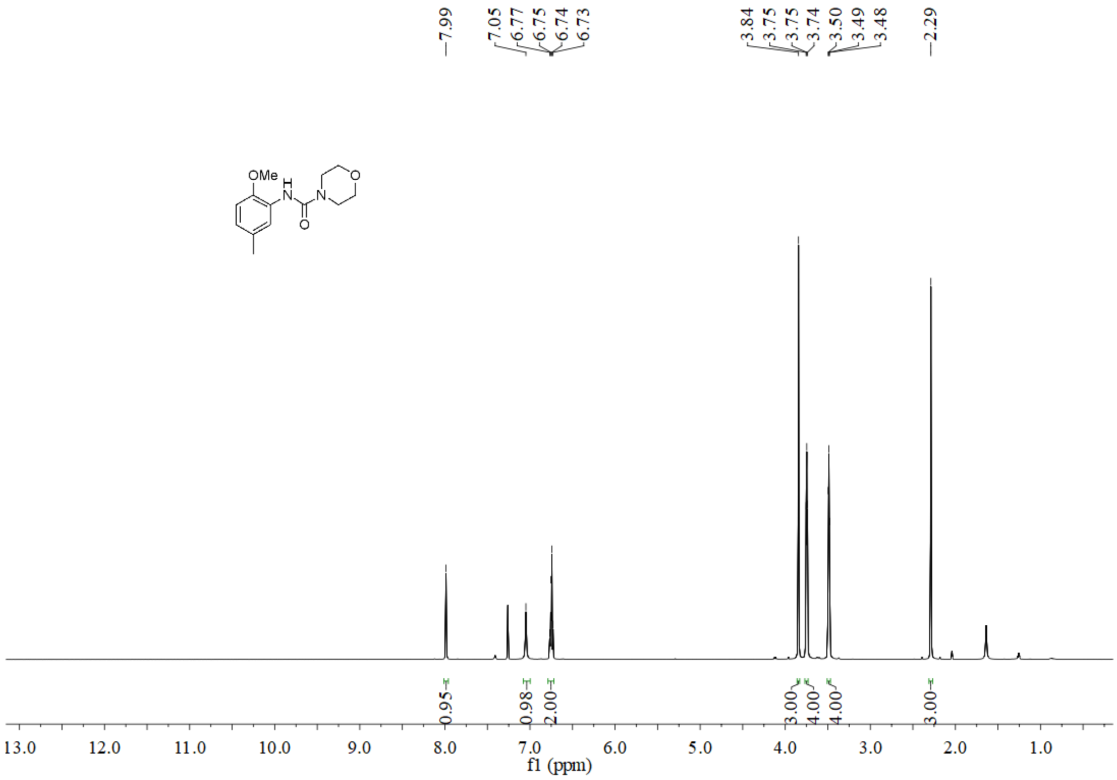

^{13}C NMR of 3f

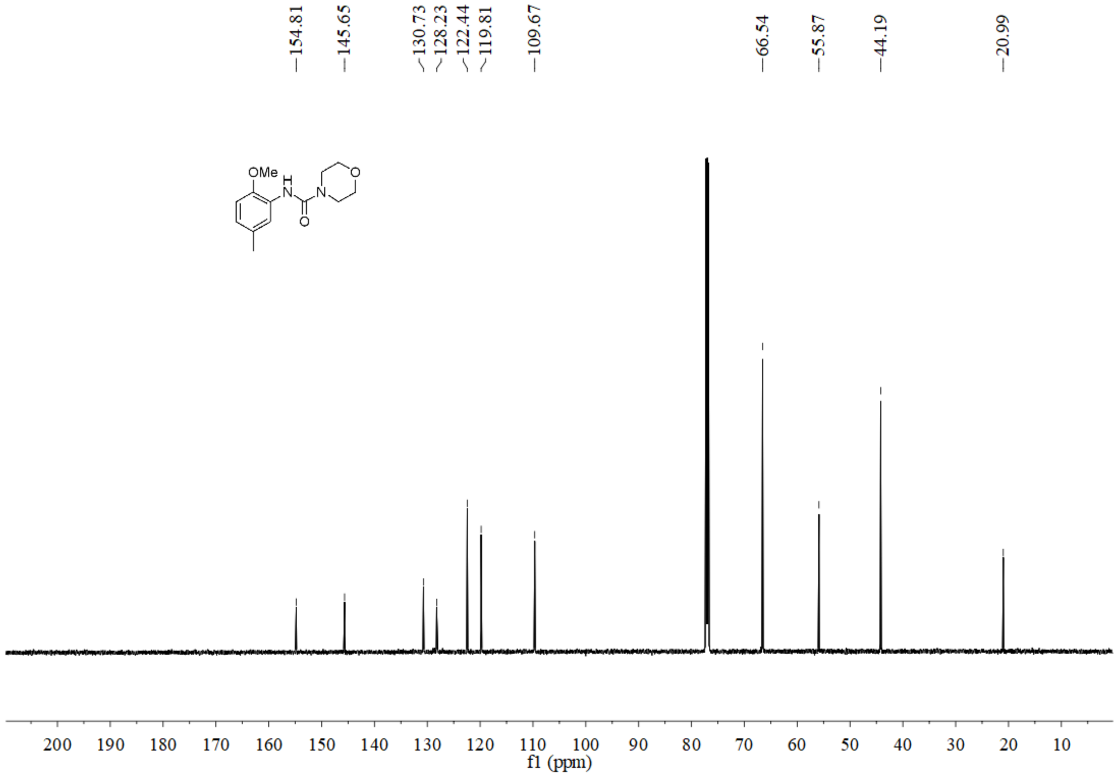

^{1}H NMR of 3g

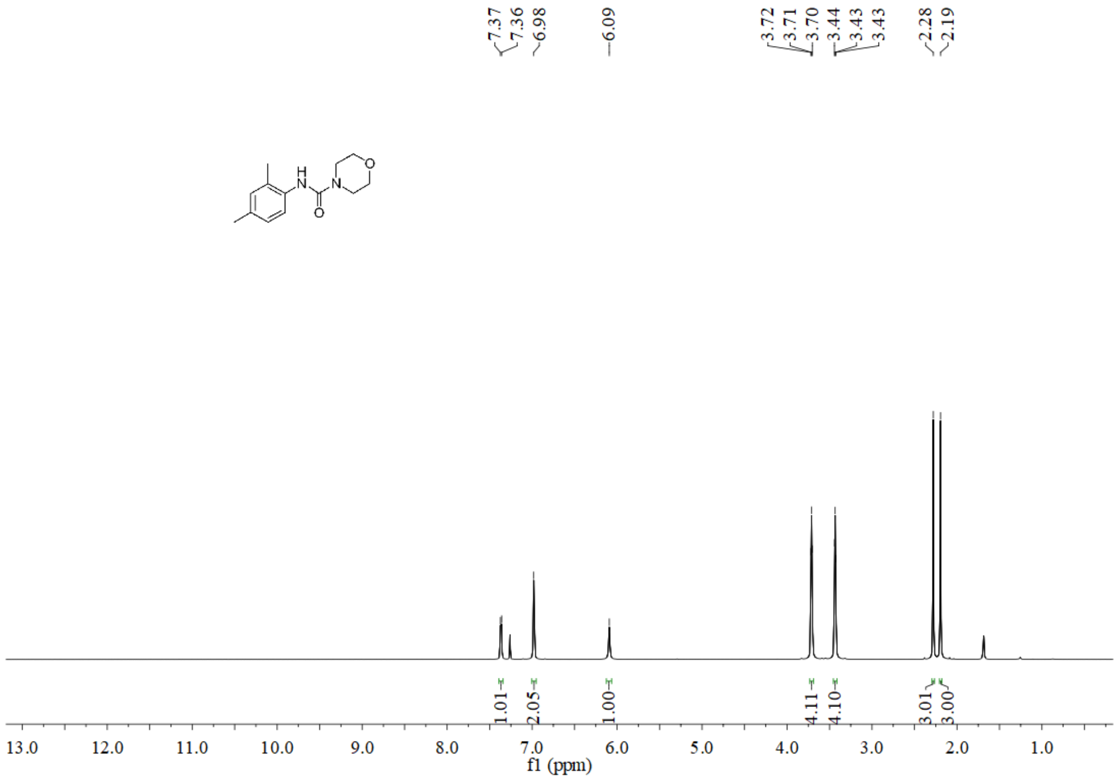

^{13}C NMR of 3g

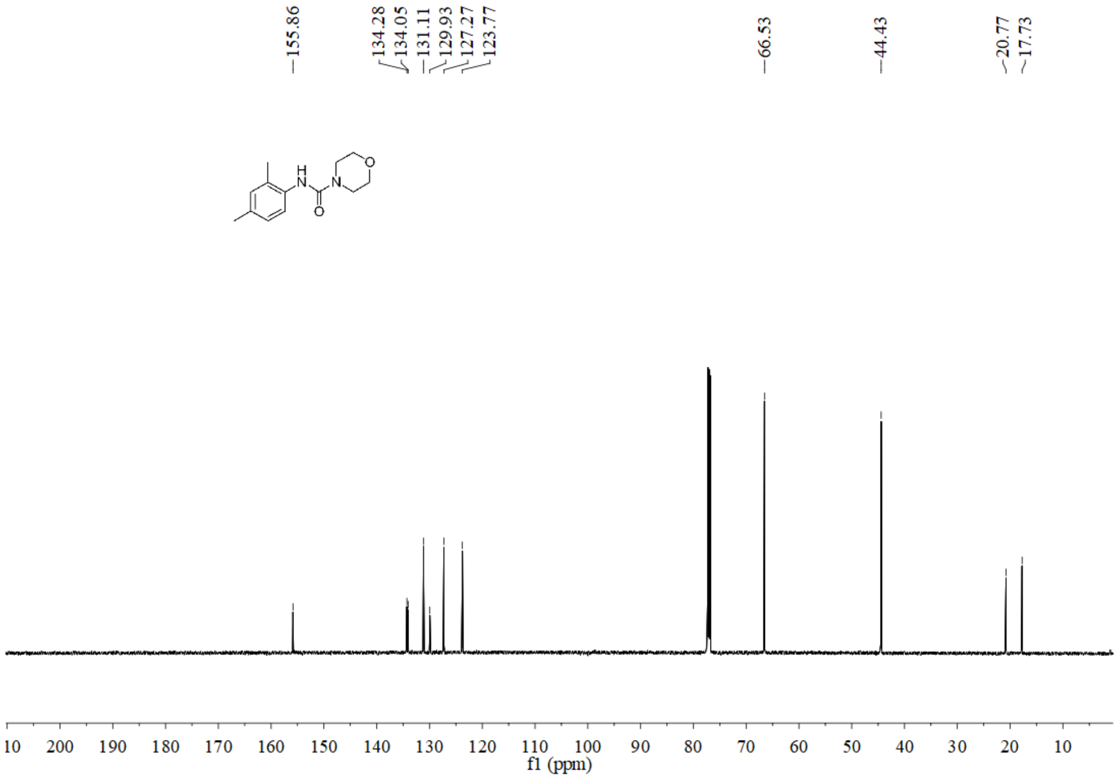

^{1}H NMR of 3h

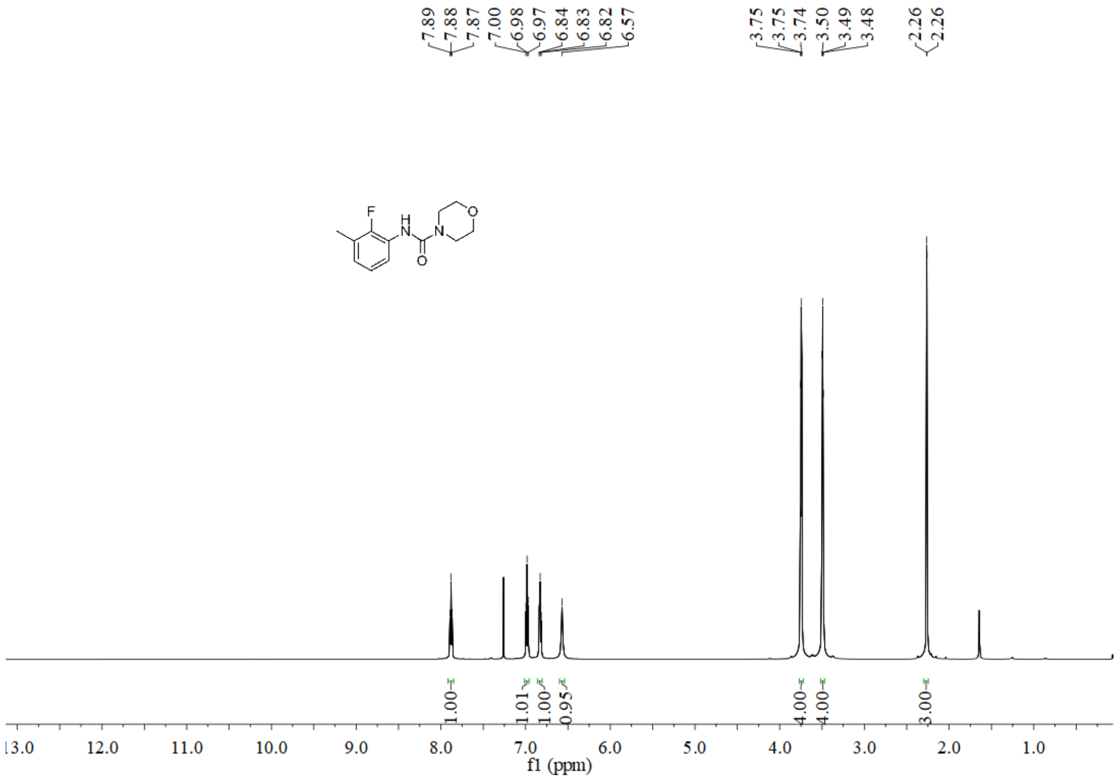

^{13}C NMR of 3h

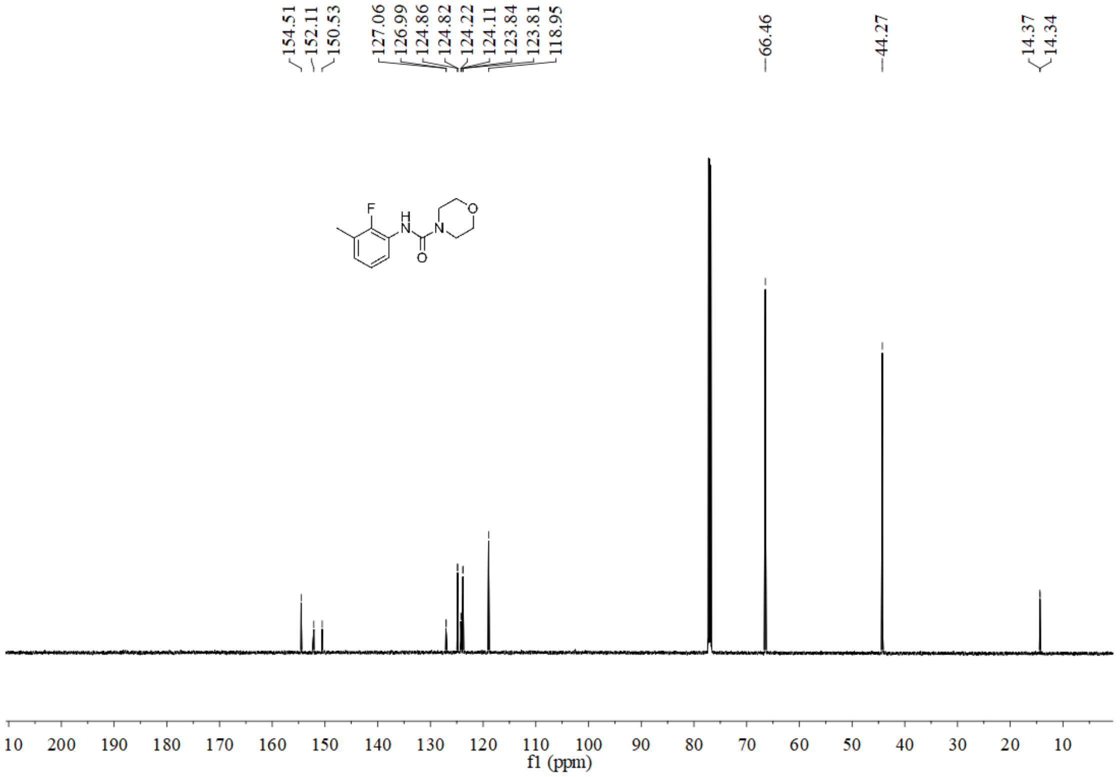

^{19}F NMR of 3h

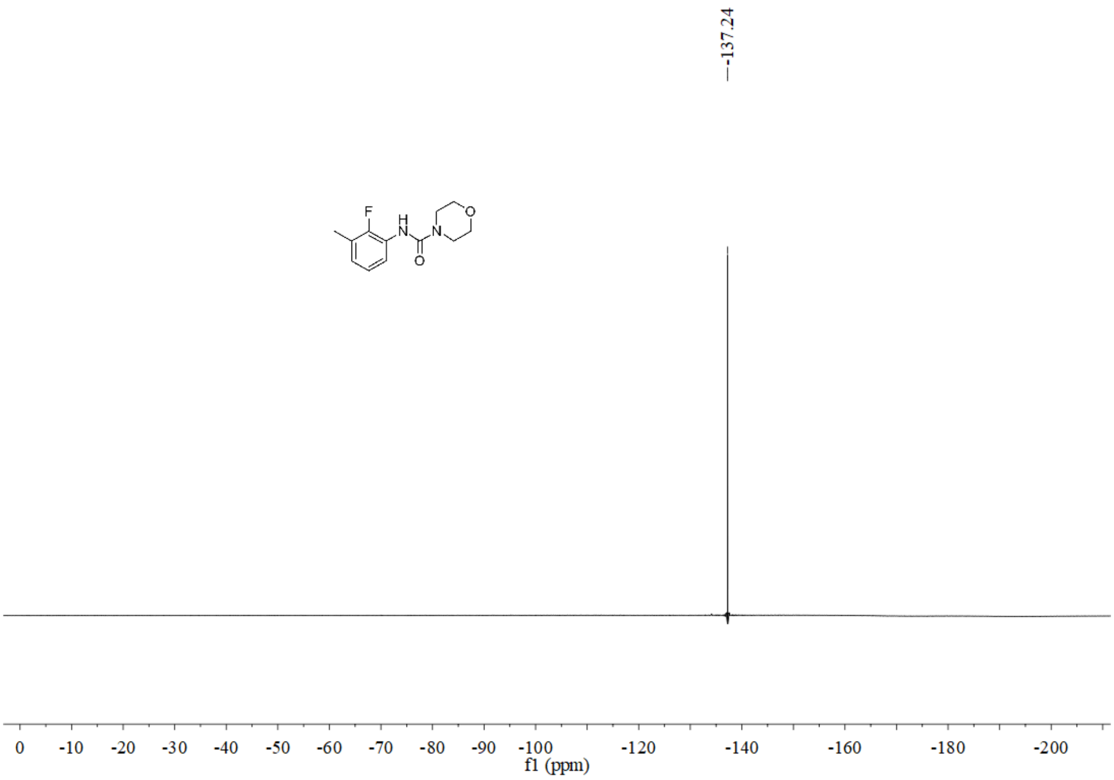

^{1}H NMR of 3i

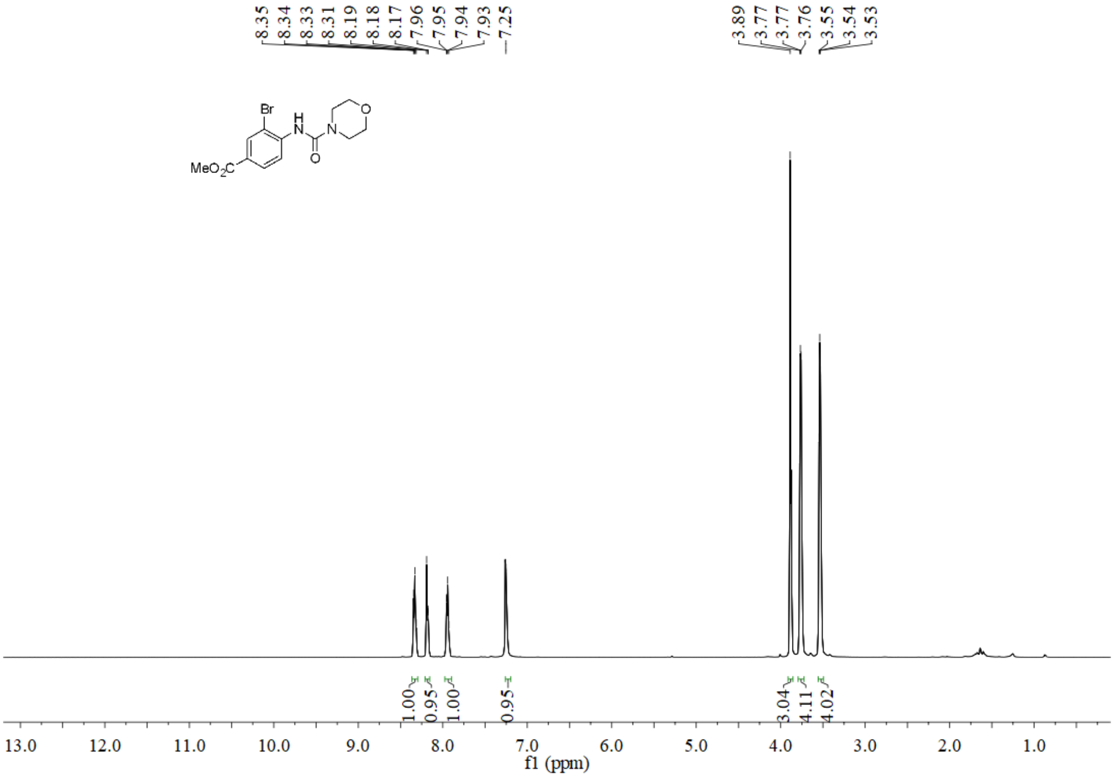

^{13}C NMR of 3i

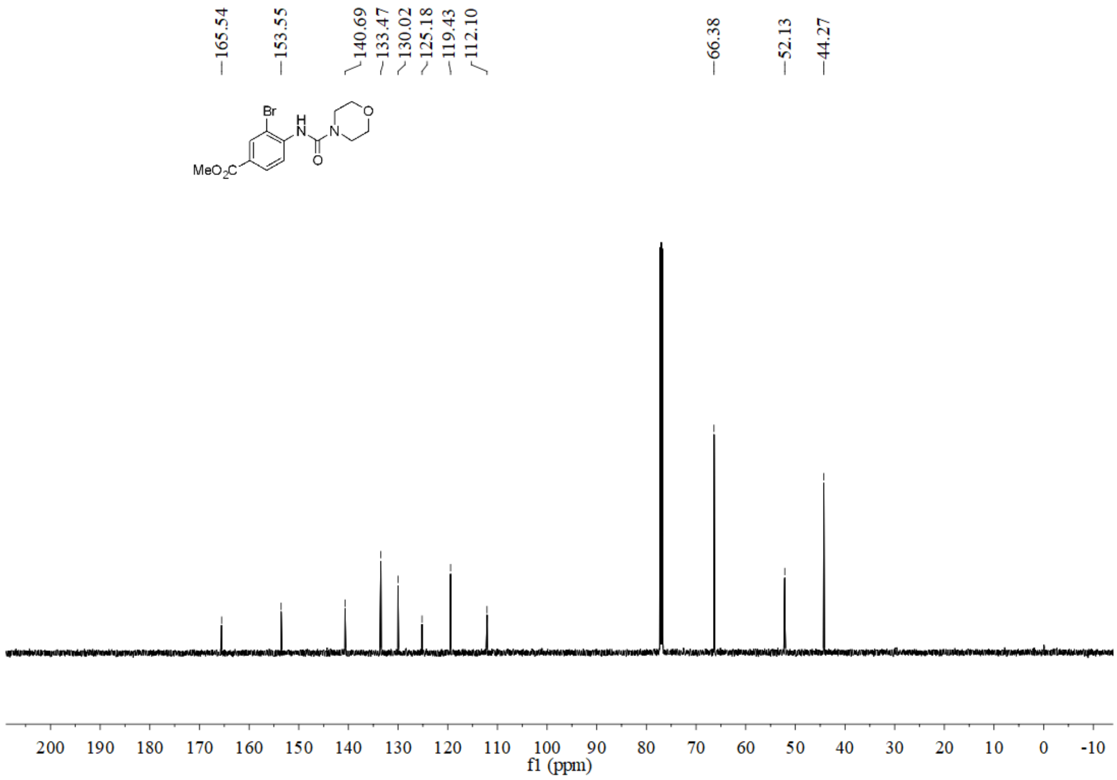

^{1}H NMR of 3j

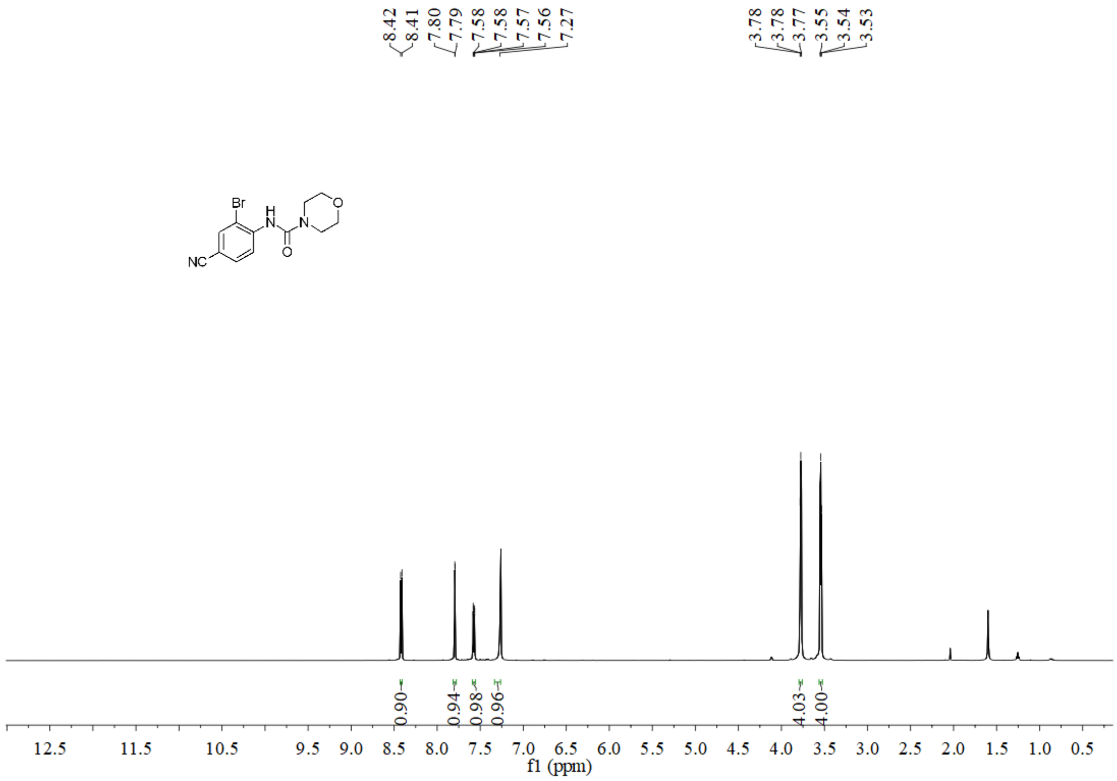

^{13}C NMR of 3j

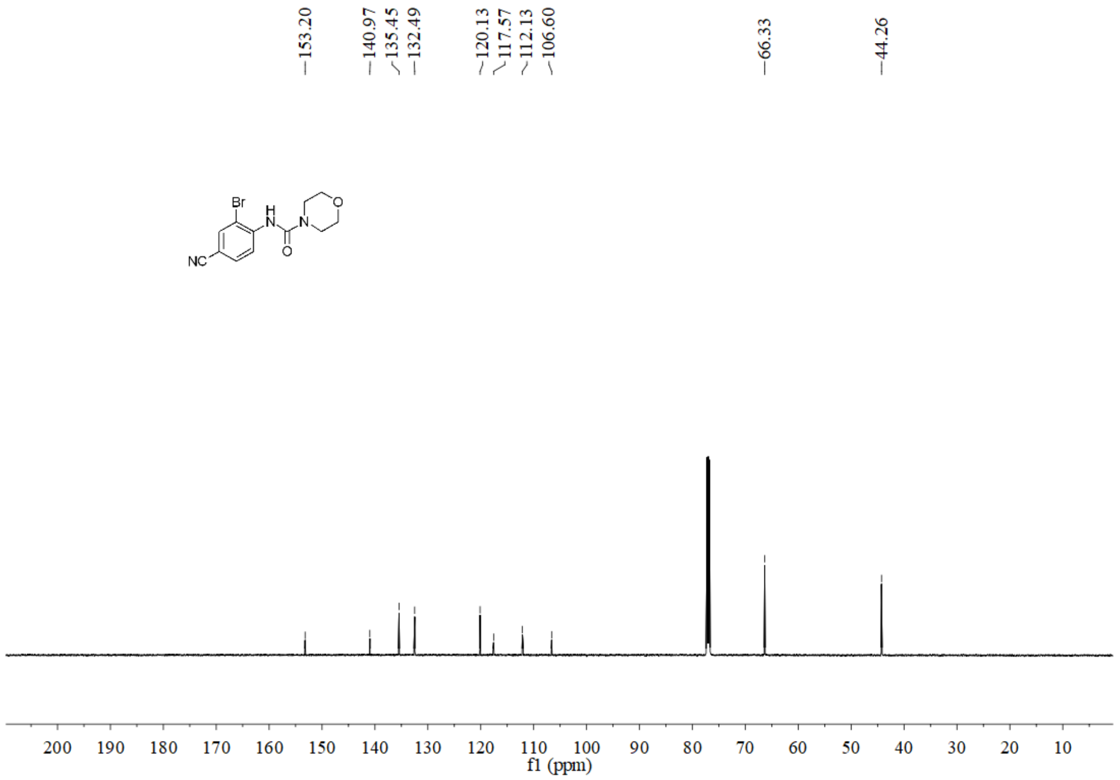

^{1}H NMR of 3k

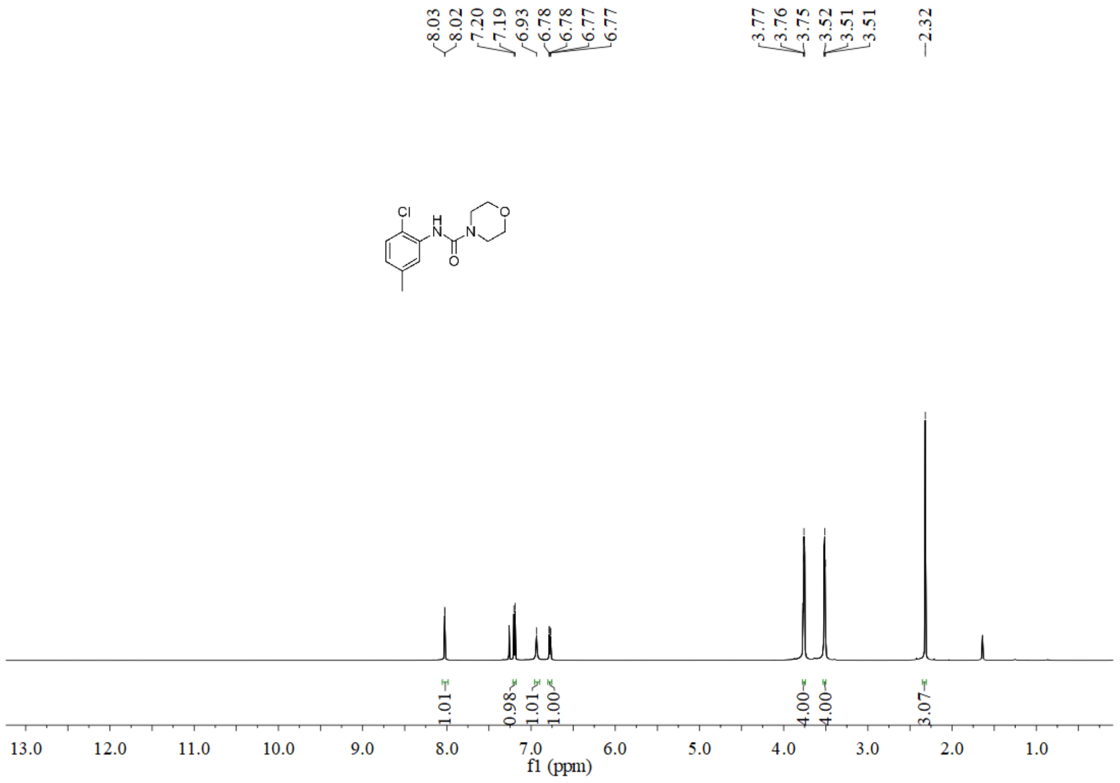

^{13}C NMR of 3k

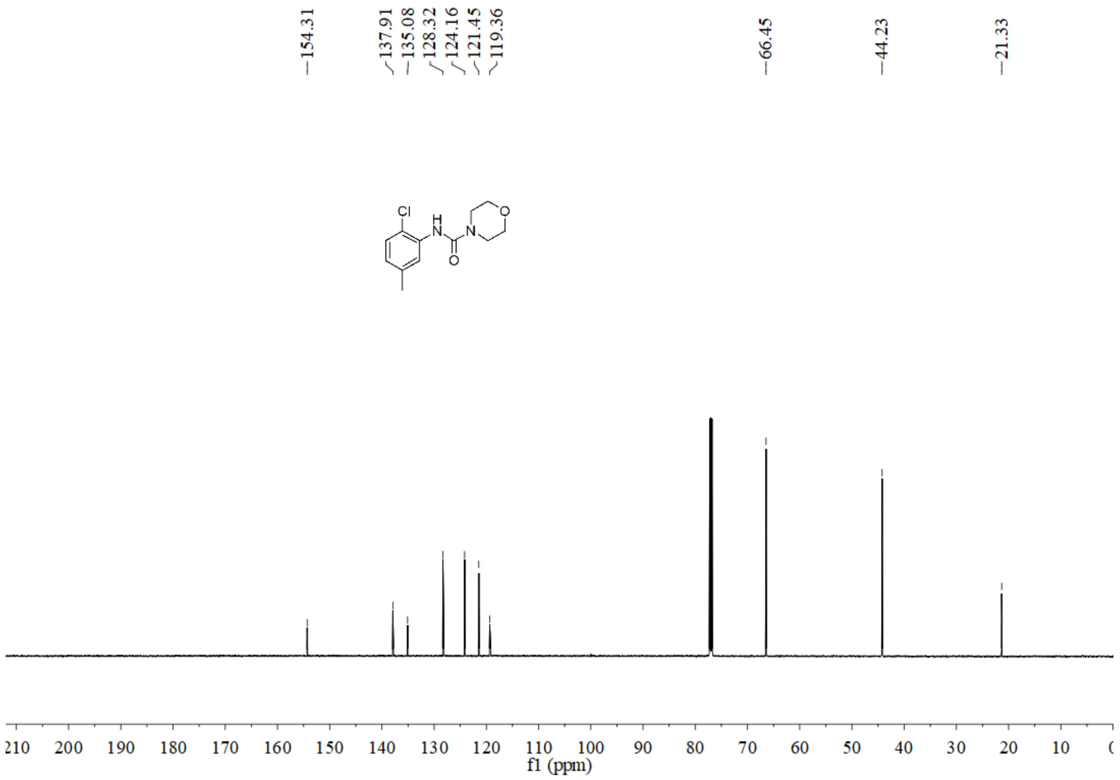

^{1}H NMR of 3l

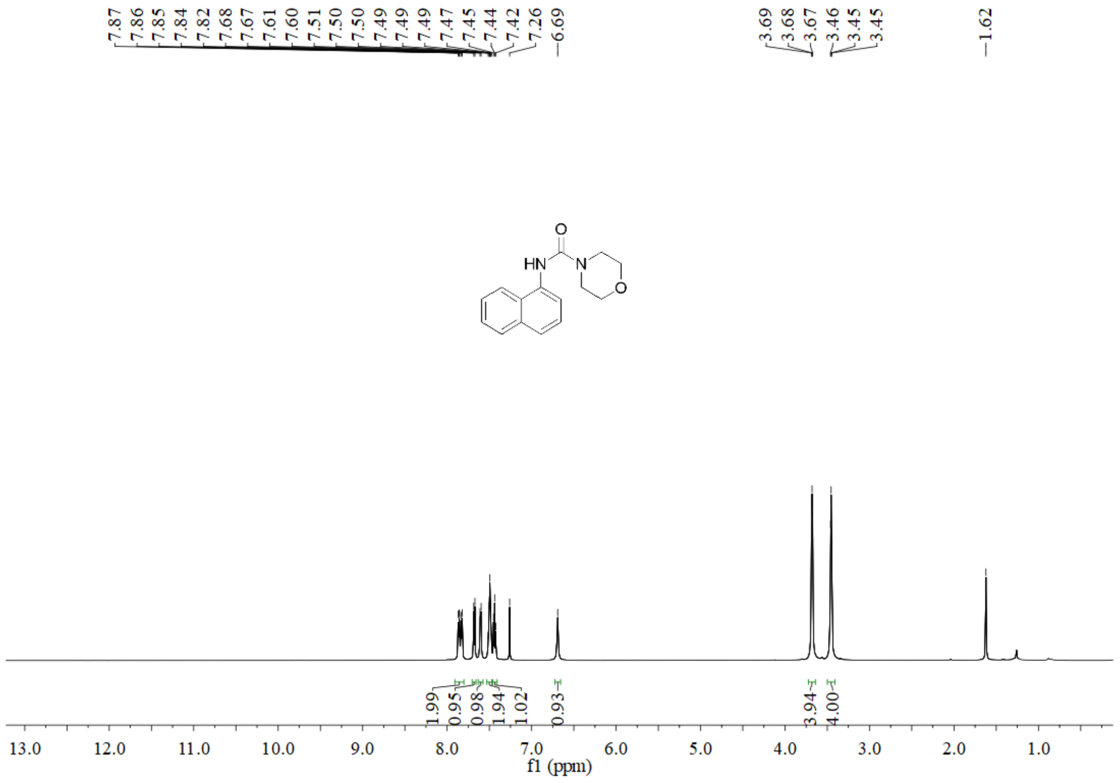

^{13}C NMR of 3l

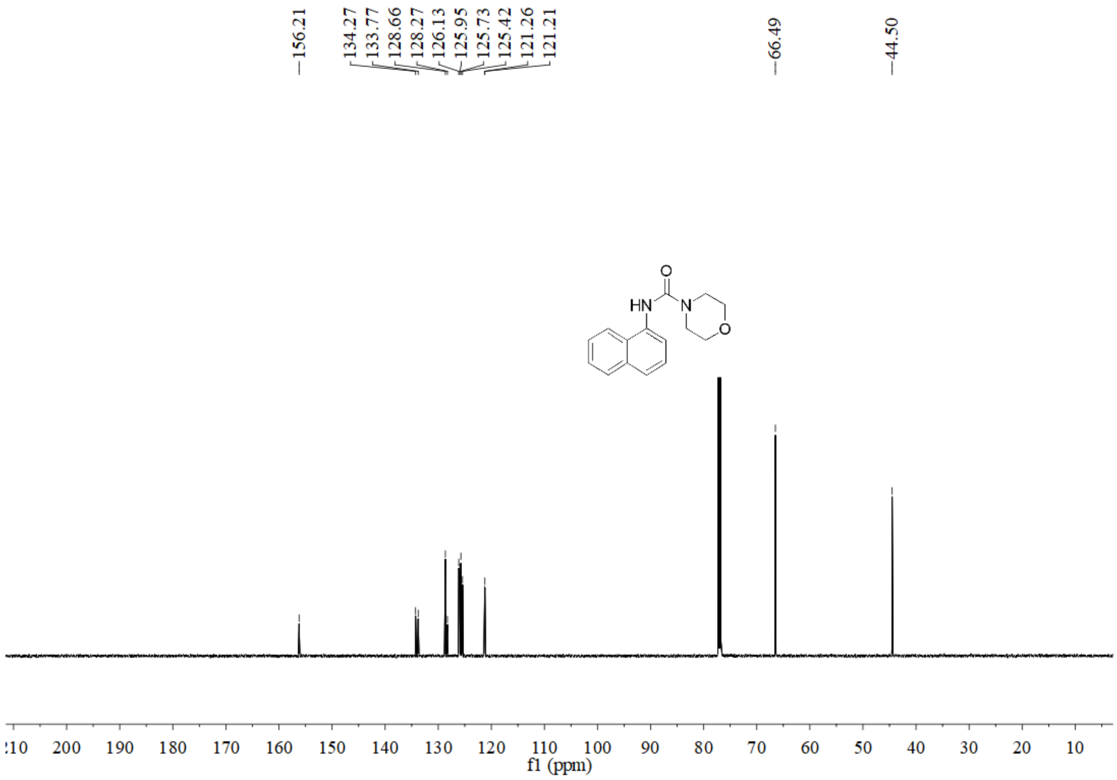

^{1}H NMR of 3m

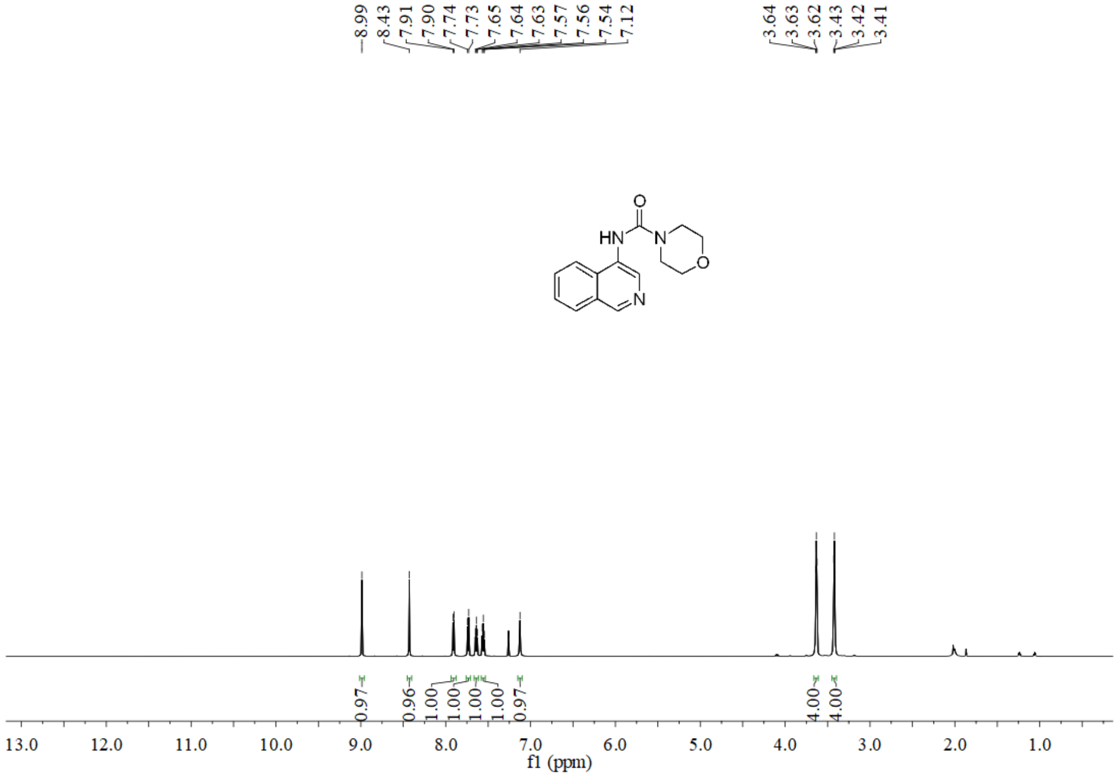

^{13}C NMR of 3m

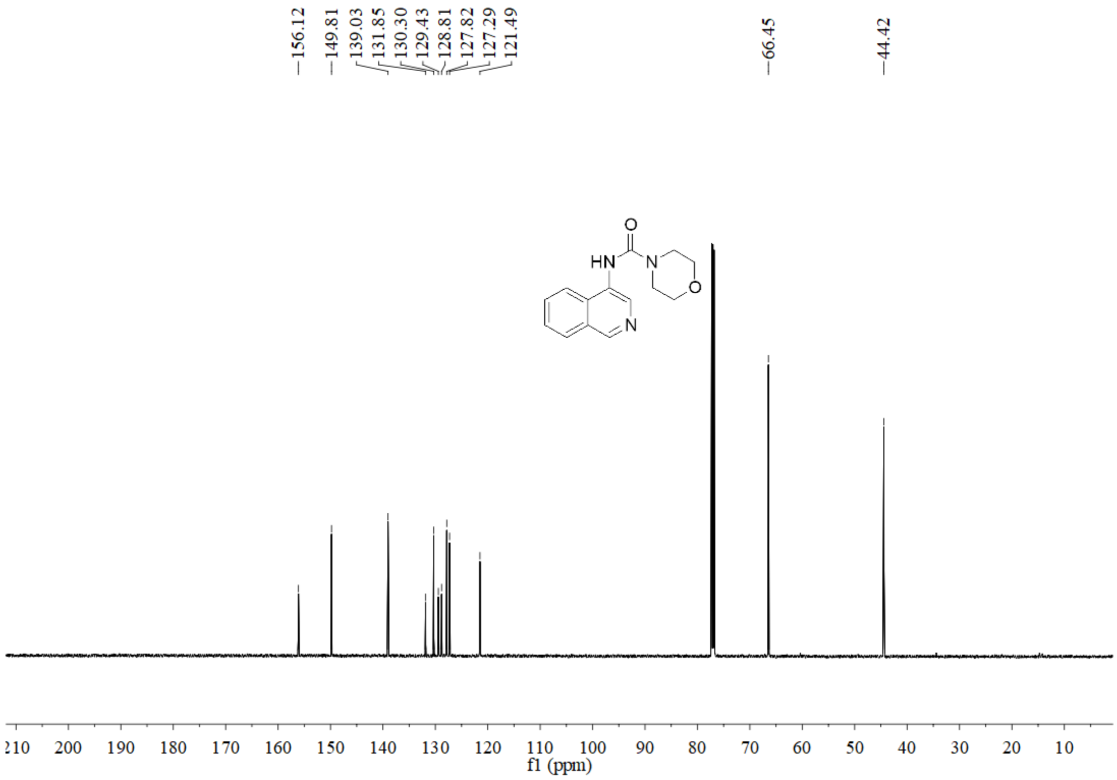

^{1}H NMR of 3n

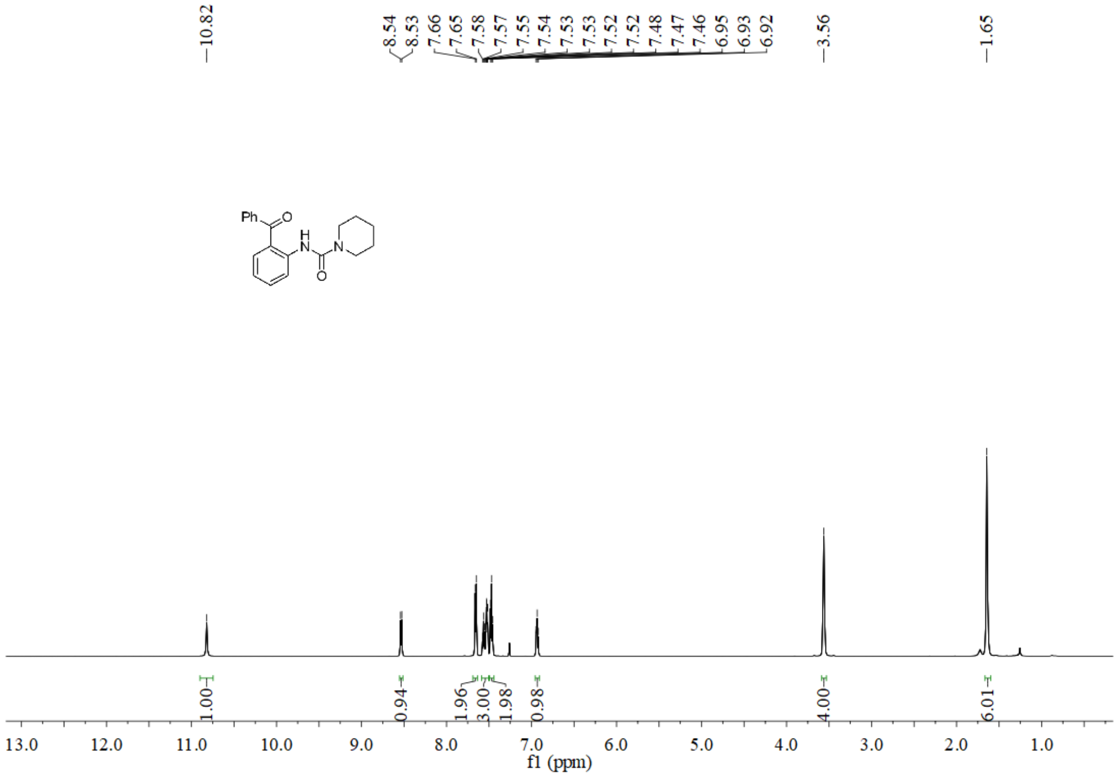

^{13}C NMR of 3n

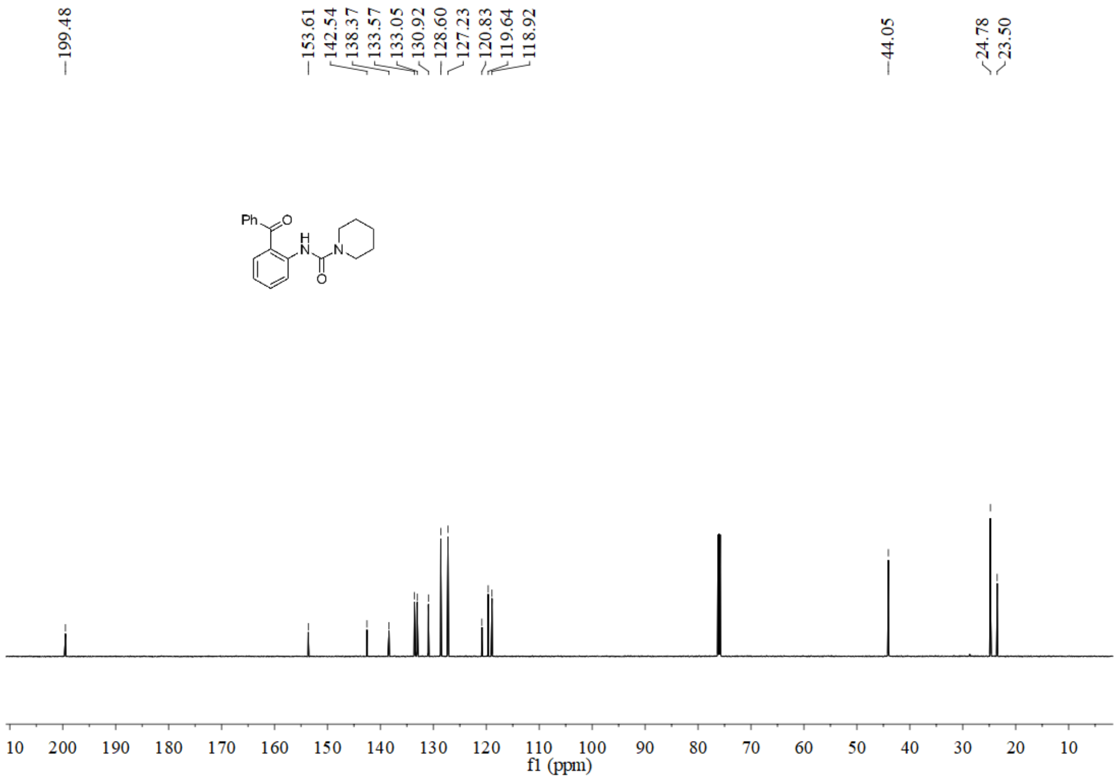

^{1}H NMR of 3o

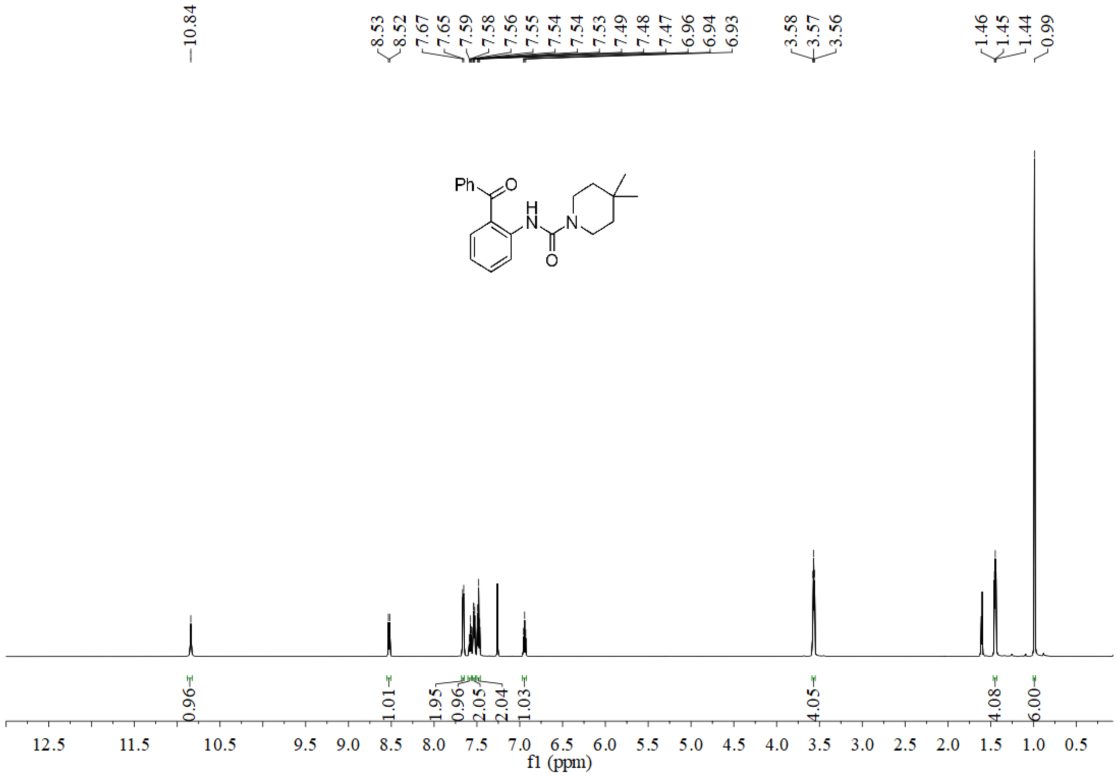

^{13}C NMR of 3o

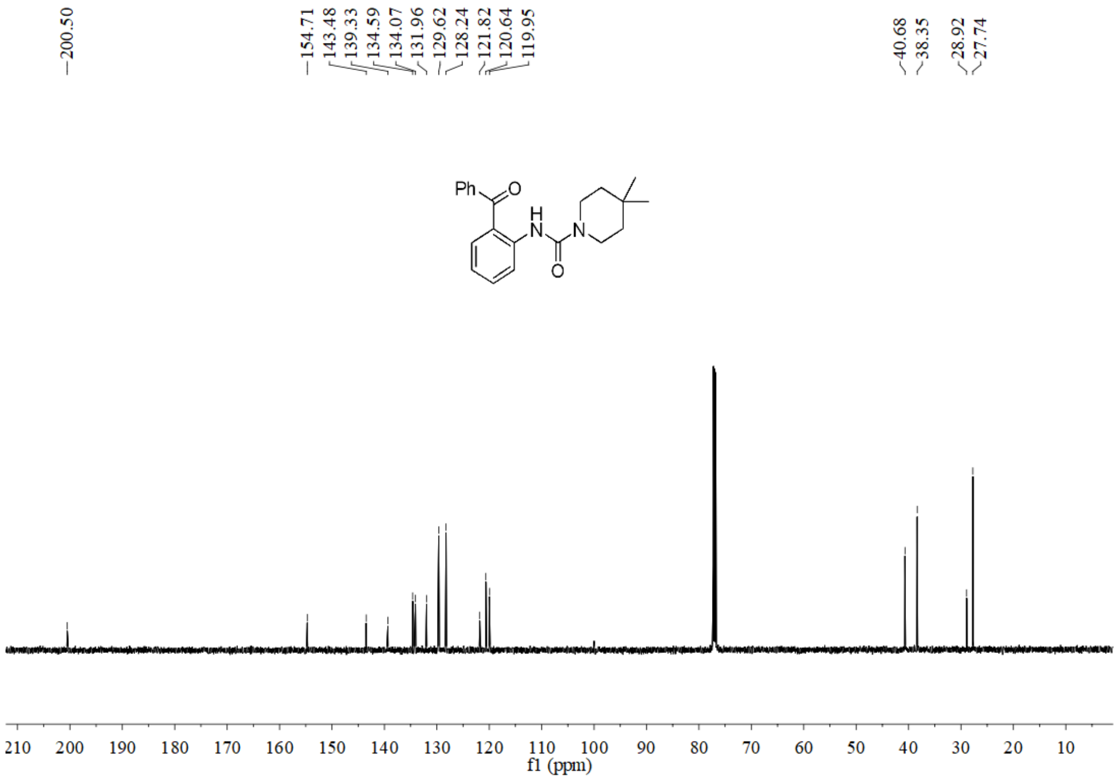

^{1}H NMR of 3p

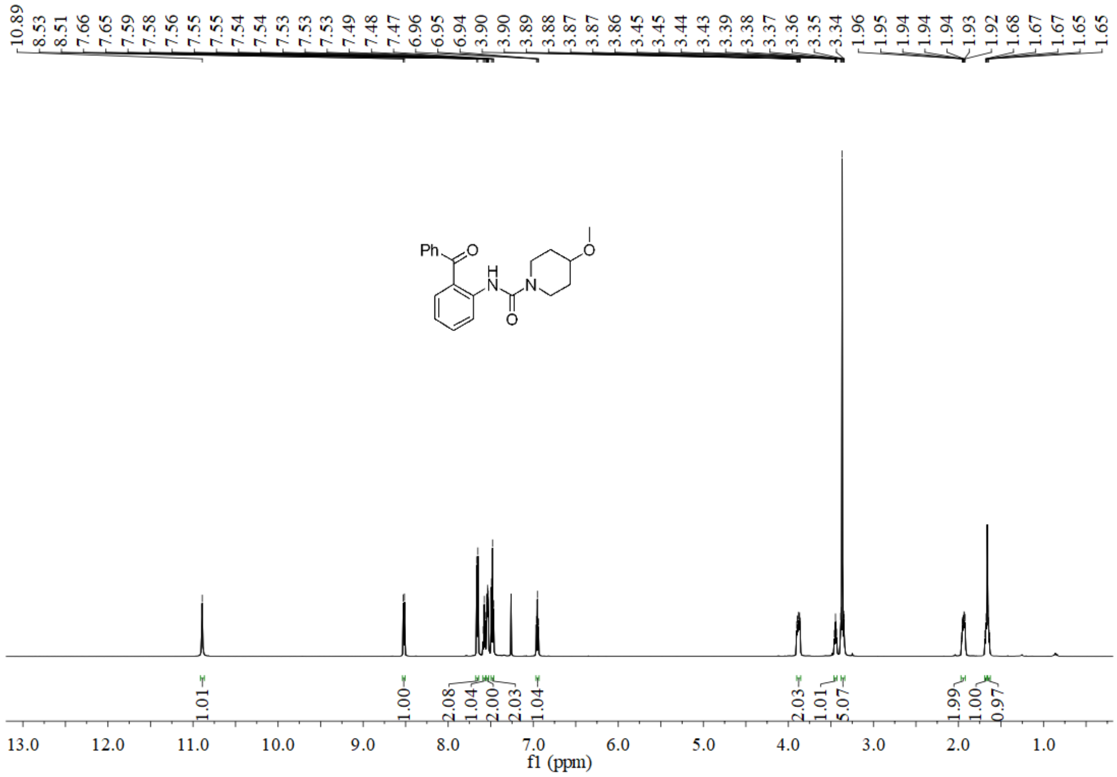

^{13}C NMR of 3p

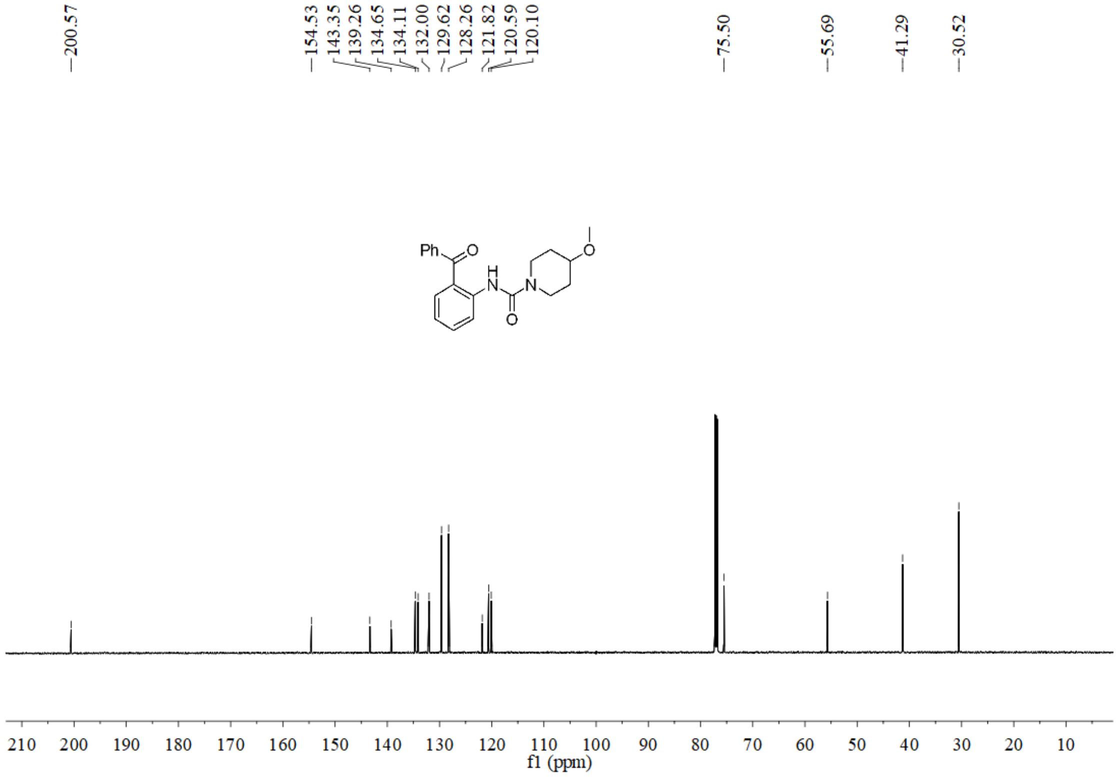

^{1}H NMR of 3q

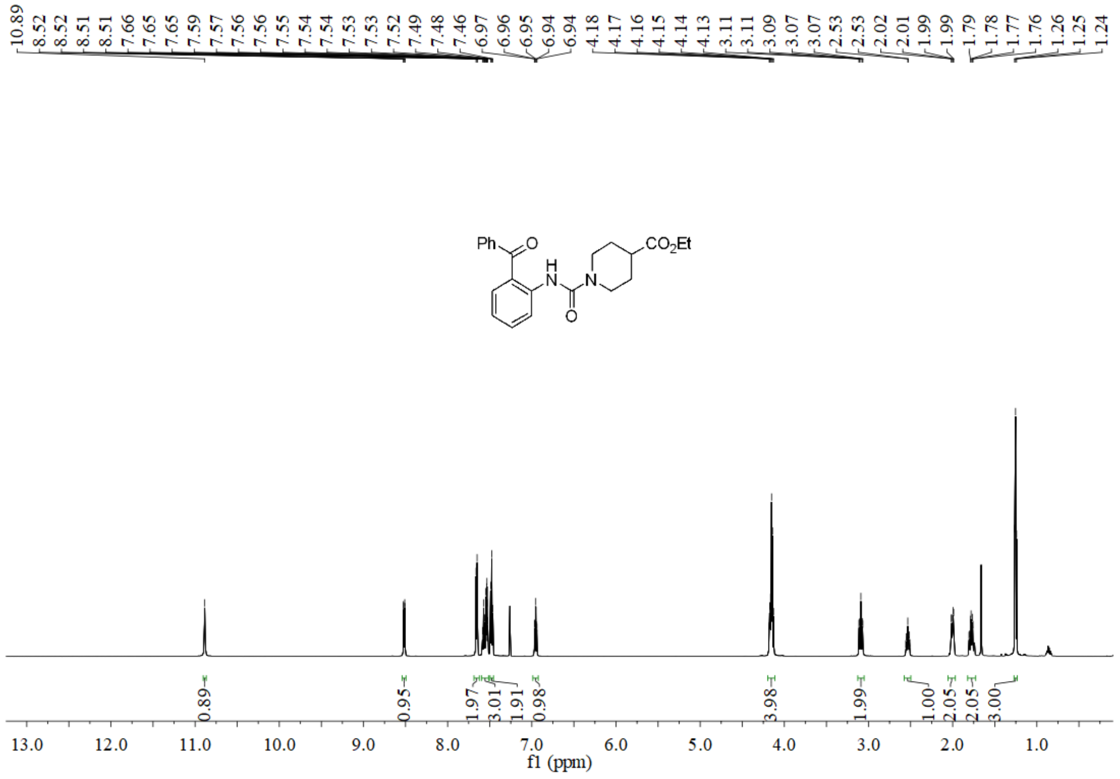

^{13}C NMR of 3q

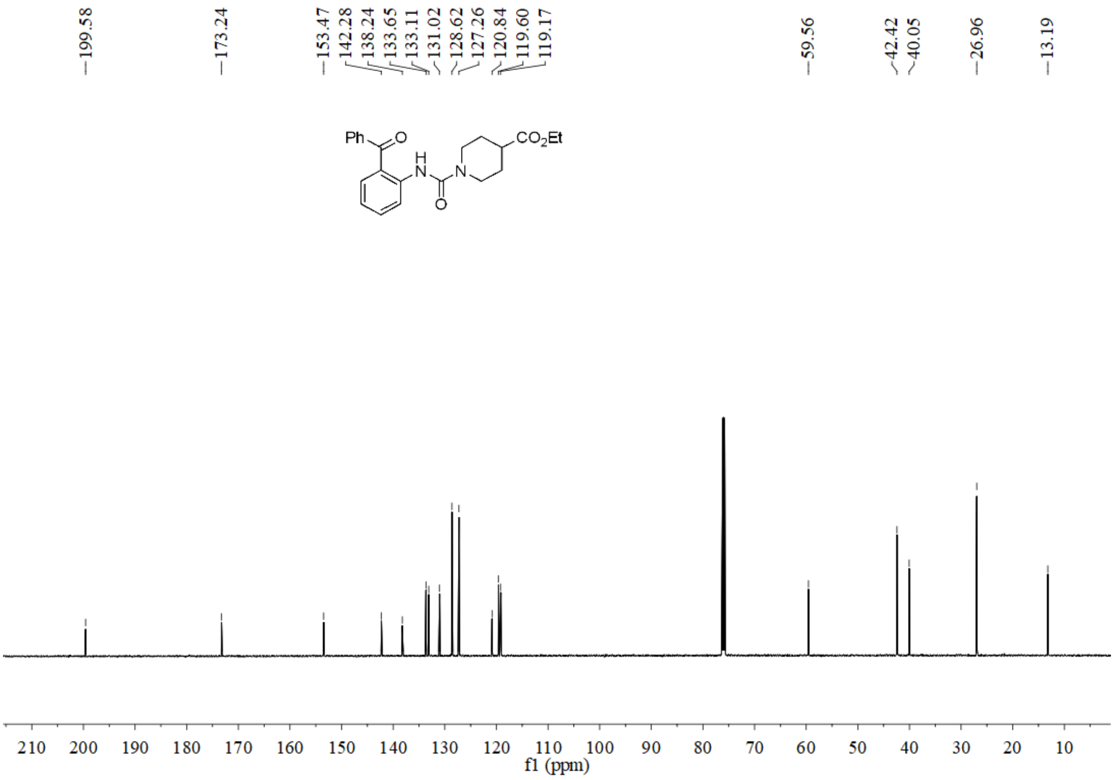

^{1}H NMR of 3r

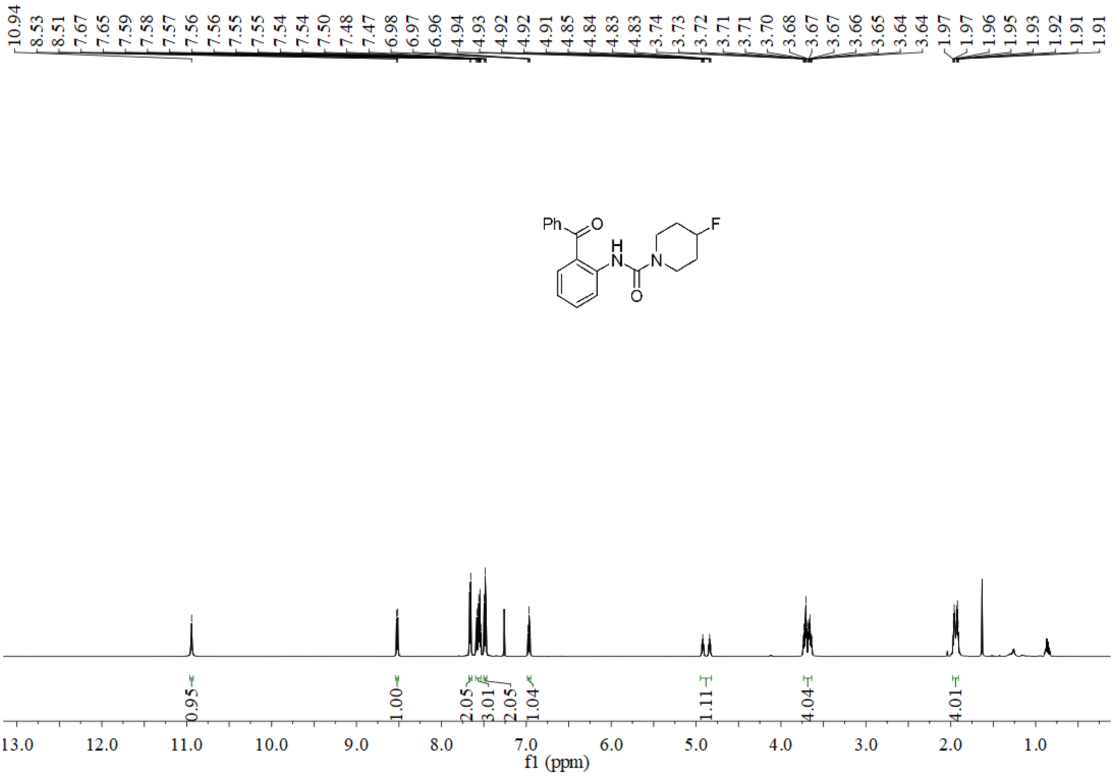

^{13}C NMR of 3r

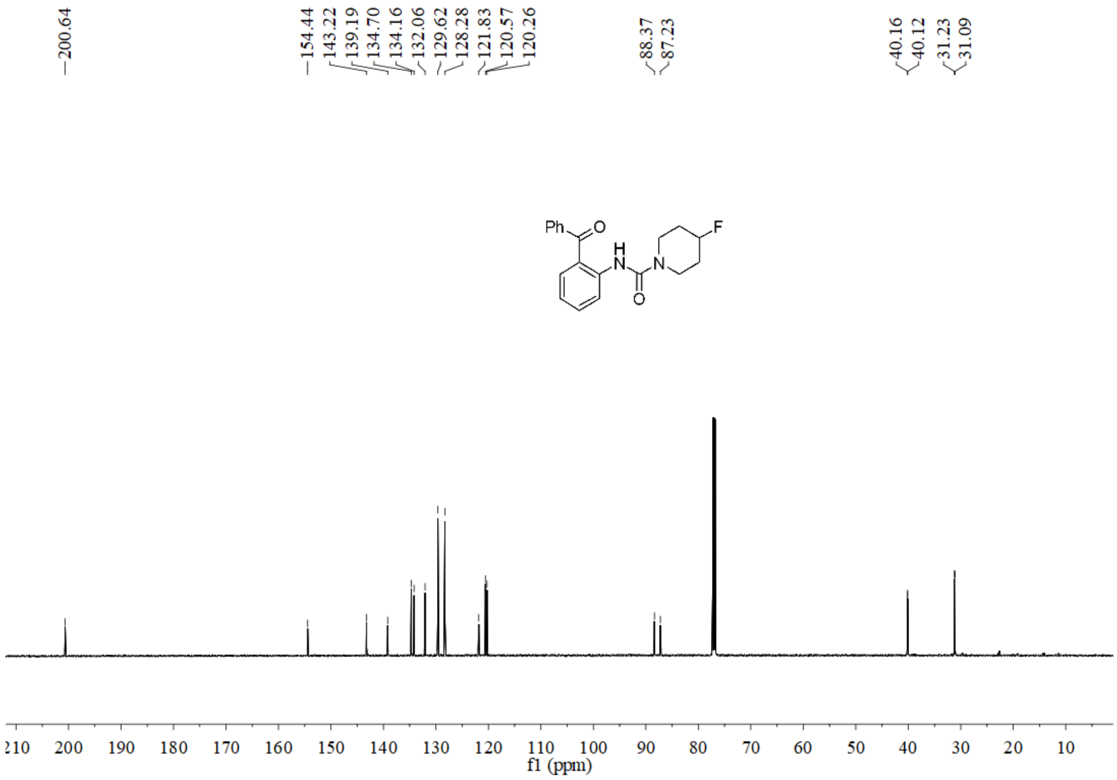

^{1}H NMR of 3s

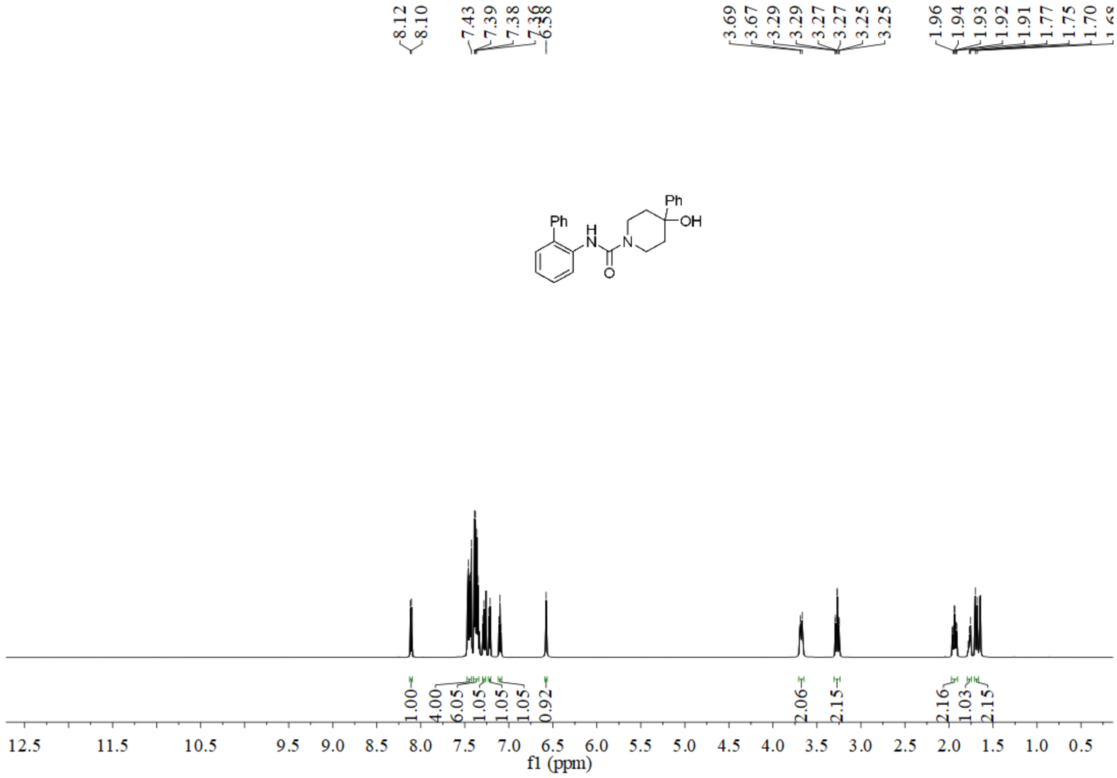

^{13}C NMR of 3s

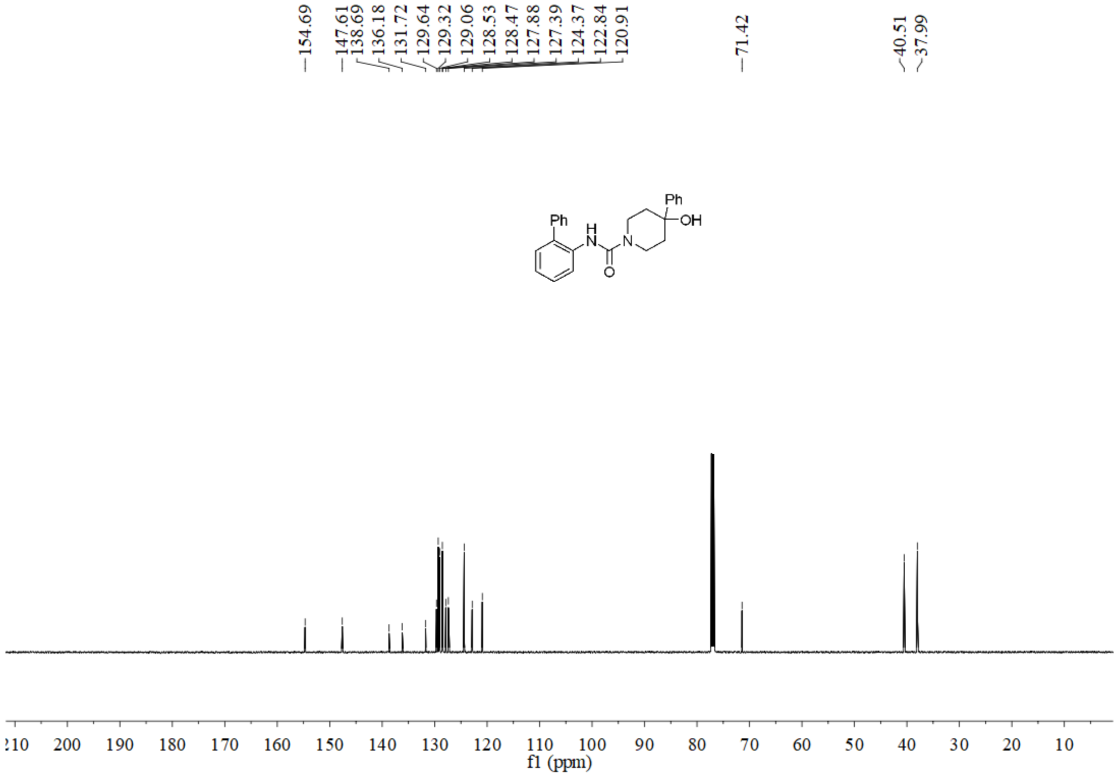

^{1}H NMR of 3t

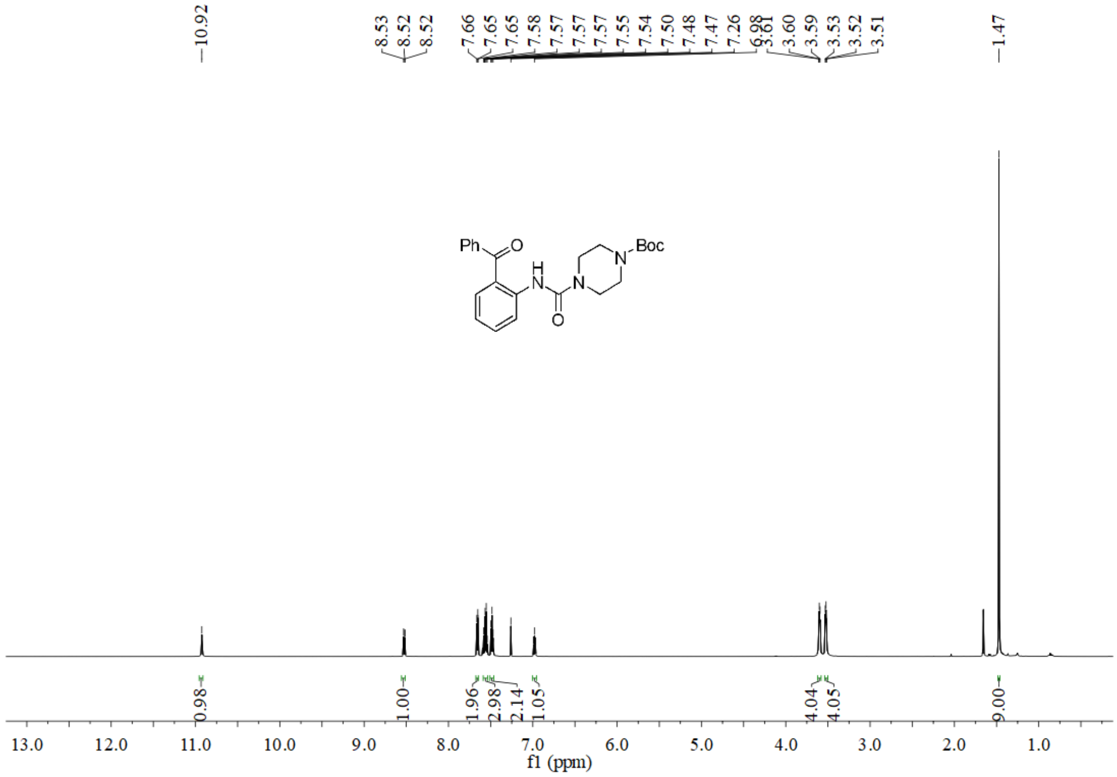

^{13}C NMR of 3t

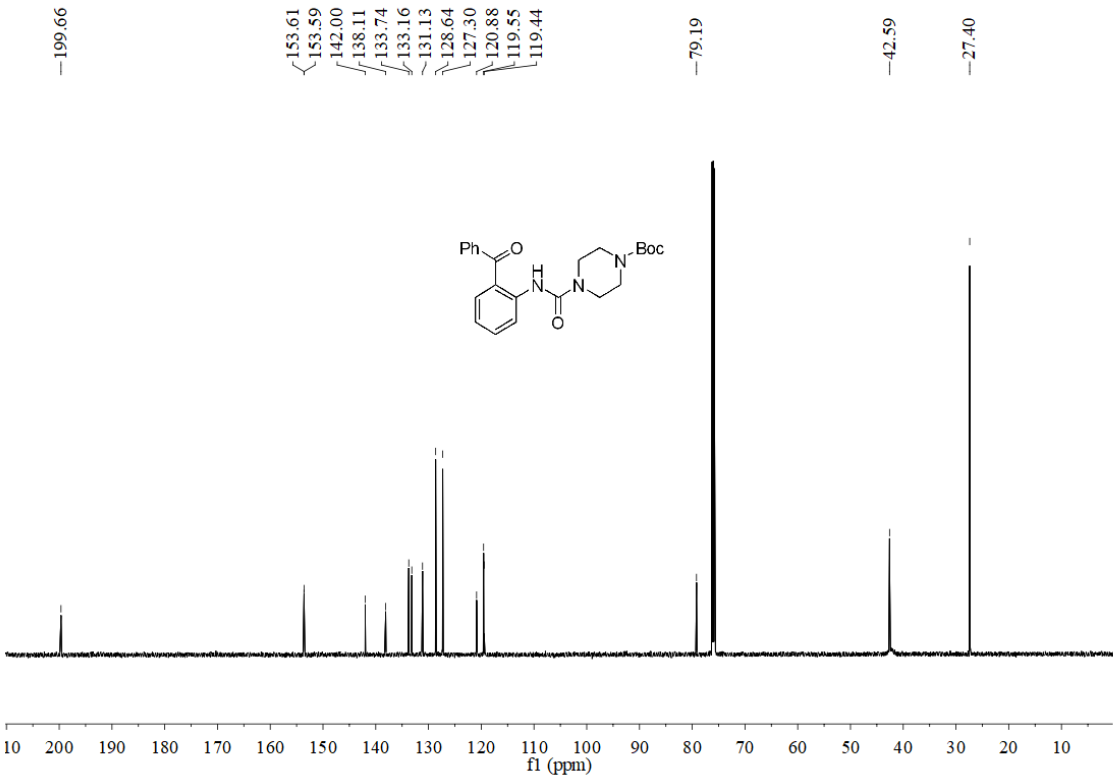

^{1}H NMR of 3u

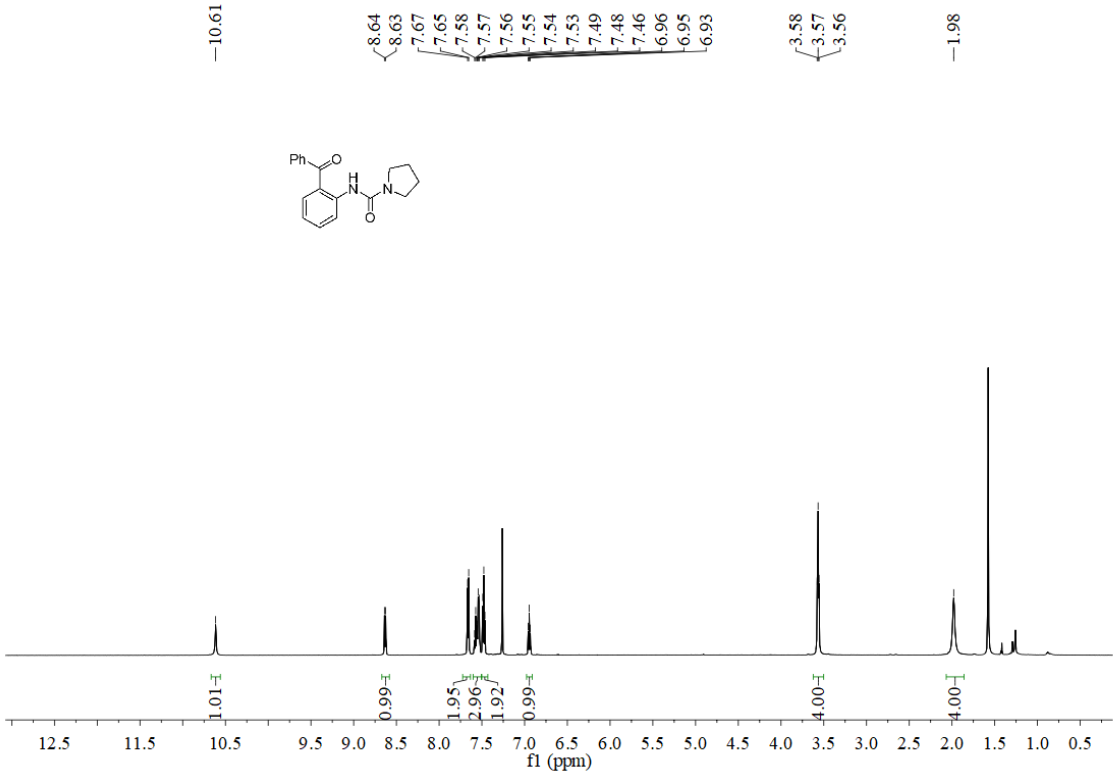

^{13}C NMR of 3u

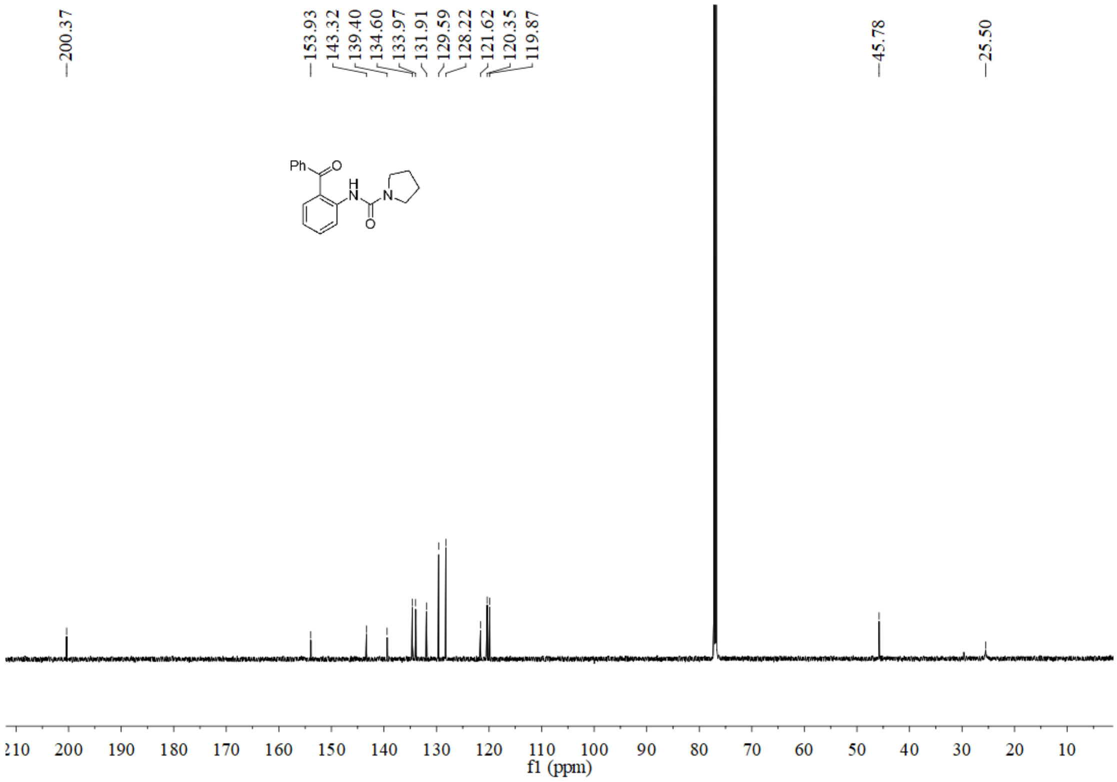

^{1}H NMR of 3v

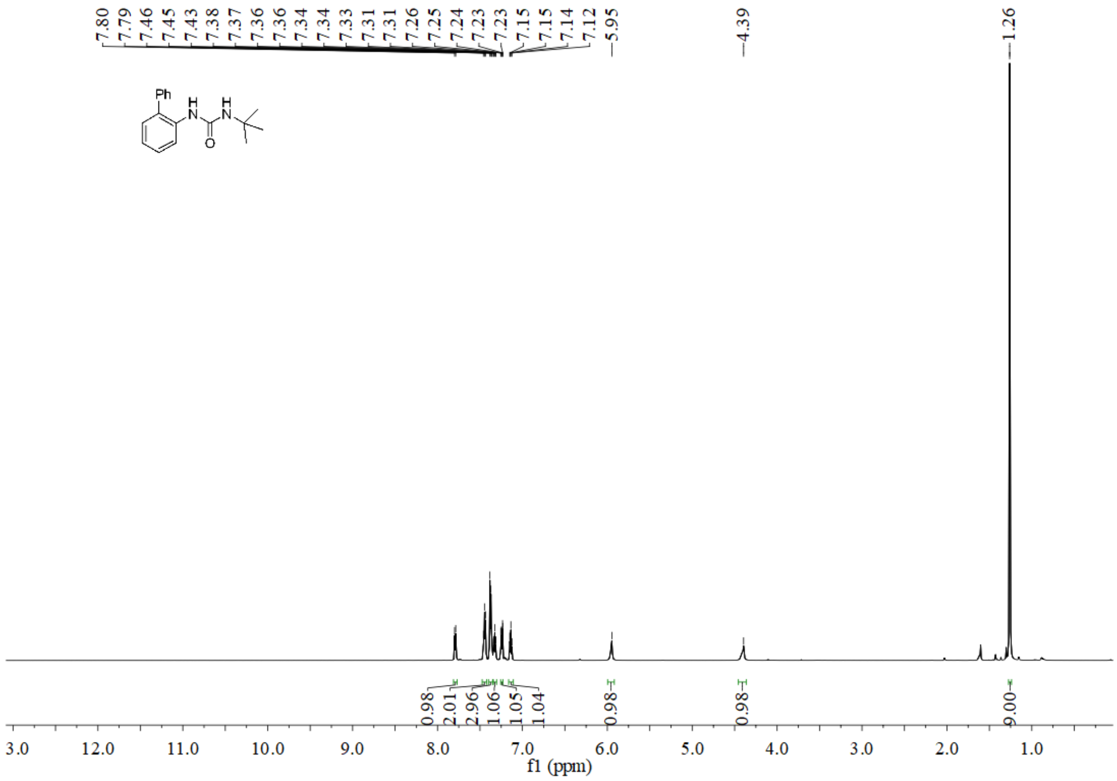

^{13}C NMR of 3v

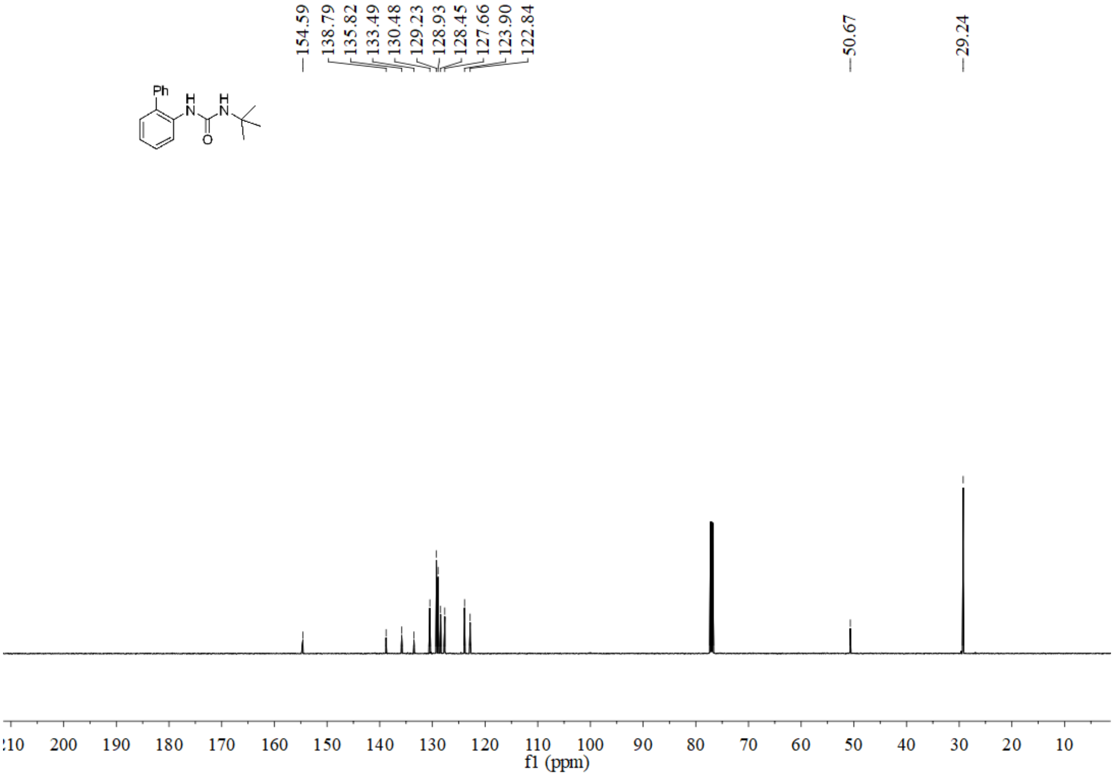

^{1}H NMR of 3w

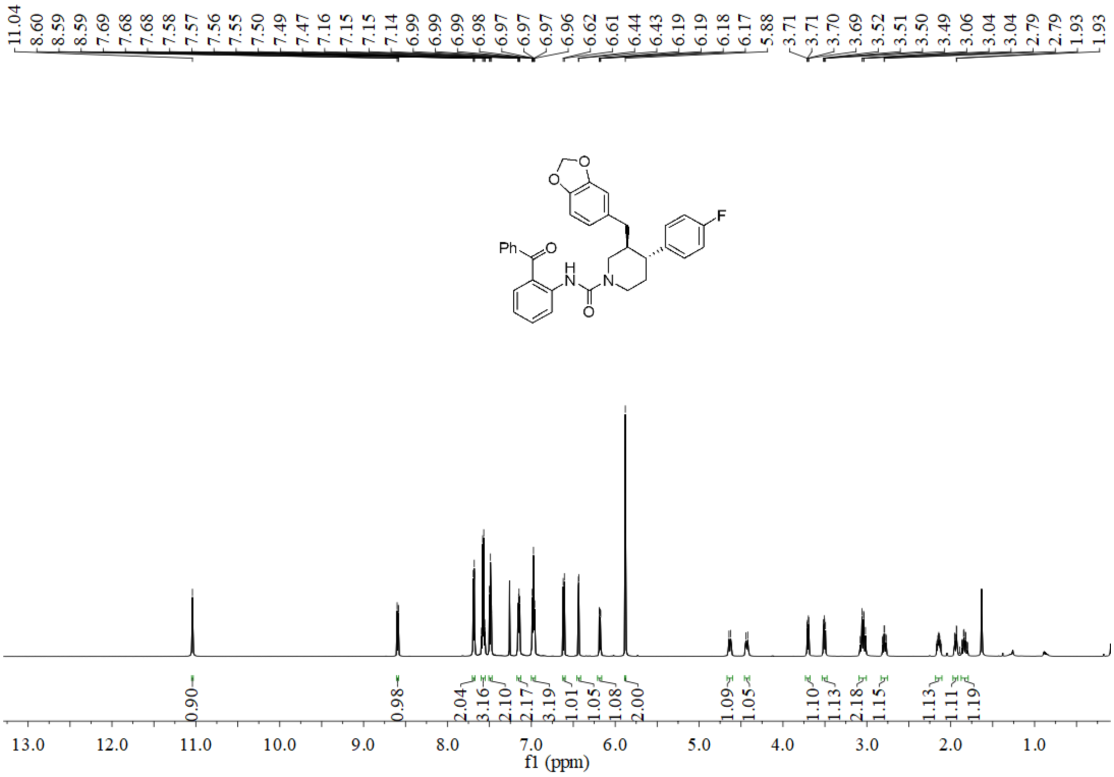

^{13}C NMR of 3w

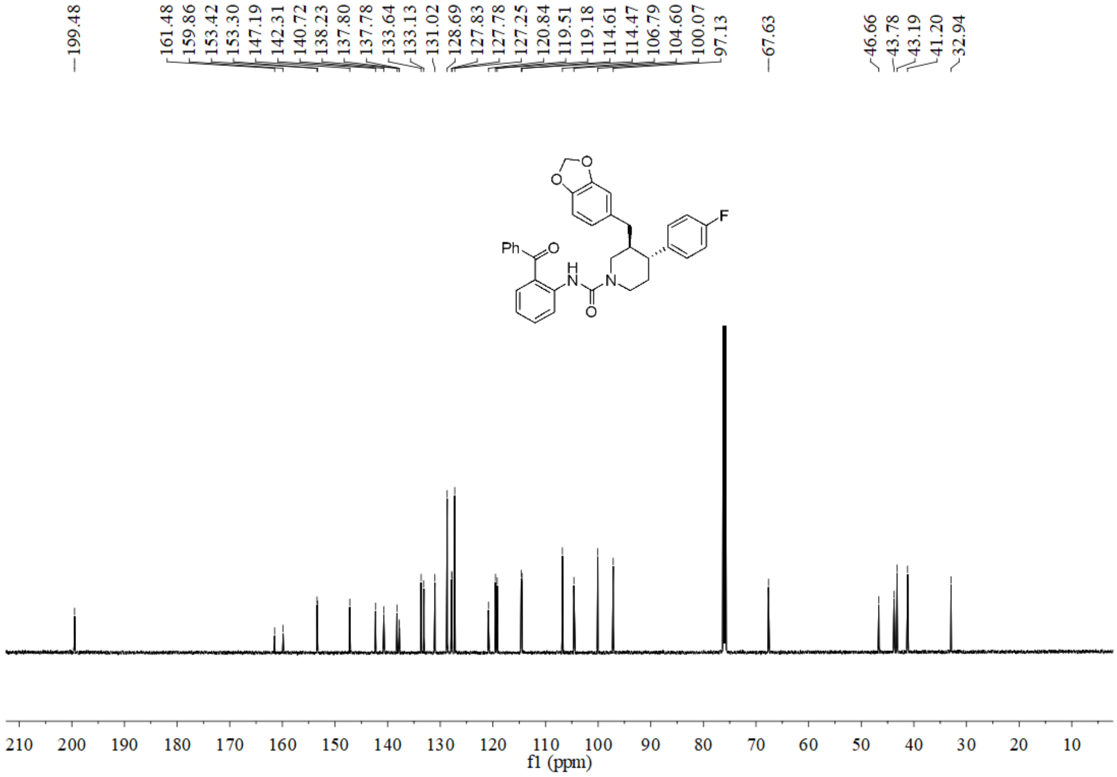

199.48
161.48
159.86
153.42
153.30
147.19
142.31
140.72
138.23
137.80
137.78
133.64
133.13
131.02
128.69
127.83
127.78
127.25
120.84
119.51
119.18
114.61
114.47
106.79
104.60
100.07
97.13
67.63
46.66
43.78
43.19
41.20
32.94
Ph
O
H
N
N
O
O
O
F
210
200
190
180
170
160
150
140
130
120
110
100
90
80
70
60
50
40
30
20
10
f1 (ppm)
